# Lambda Red Mediated Gap Repair Utilizes a Novel Replicative Intermediate in *Escherichia coli*

**DOI:** 10.1371/journal.pone.0120681

**Published:** 2015-03-24

**Authors:** Thimma R. Reddy, Léna M. S. Fevat, Sarah E. Munson, A. Francis Stewart, Shaun M. Cowley

**Affiliations:** 1 Department of Biochemistry, University of Leicester, Leicester, United Kingdom; 2 Center for Fisheries, Environment and Aquaculture Sciences, Lowestoft, United Kingdom; 3 ES Cell Facility, Centre for Core Biotechnology Services, University of Leicester, Leicester, United Kingdom; 4 Genomics, BioInnovationsZentrum, Technische Universitaet Dresden, Dresden, Germany; Saint Louis University, UNITED STATES

## Abstract

The lambda phage Red recombination system can mediate efficient homologous recombination in *Escherichia coli*, which is the basis of the DNA engineering technique termed recombineering. Red mediated insertion of DNA requires DNA replication, involves a single-stranded DNA intermediate and is more efficient on the lagging strand of the replication fork. Lagging strand recombination has also been postulated to explain the Red mediated repair of gapped plasmids by an Okazaki fragment gap filling model. Here, we demonstrate that gap repair involves a different strand independent mechanism. Gap repair assays examining the strand asymmetry of recombination did not show a lagging strand bias. Directly testing an ssDNA plasmid showed lagging strand recombination is possible but dsDNA plasmids did not employ this mechanism. Insertional recombination combined with gap repair also did not demonstrate preferential lagging strand bias, supporting a different gap repair mechanism. The predominant recombination route involved concerted insertion and subcloning though other routes also operated at lower frequencies. Simultaneous insertion of DNA resulted in modification of both strands and was unaffected by mutations to DNA polymerase I, responsible for Okazaki fragment maturation. The lower efficiency of an alternate Red mediated ends-in recombination pathway and the apparent lack of a Holliday junction intermediate suggested that gap repair does not involve a different Red recombination pathway. Our results may be explained by a novel replicative intermediate in gap repair that does not involve a replication fork. We exploited these observations by developing a new recombineering application based on concerted insertion and gap repair, termed SPI (subcloning plus insertion). SPI selected against empty vector background and selected for correct gap repair recombinants. We used SPI to simultaneously insert up to four different gene cassettes in a single recombineering reaction. Consequently, our findings have important implications for the understanding of *E*. *coli* replication and Red recombination.

## Introduction

**Fig 1:**
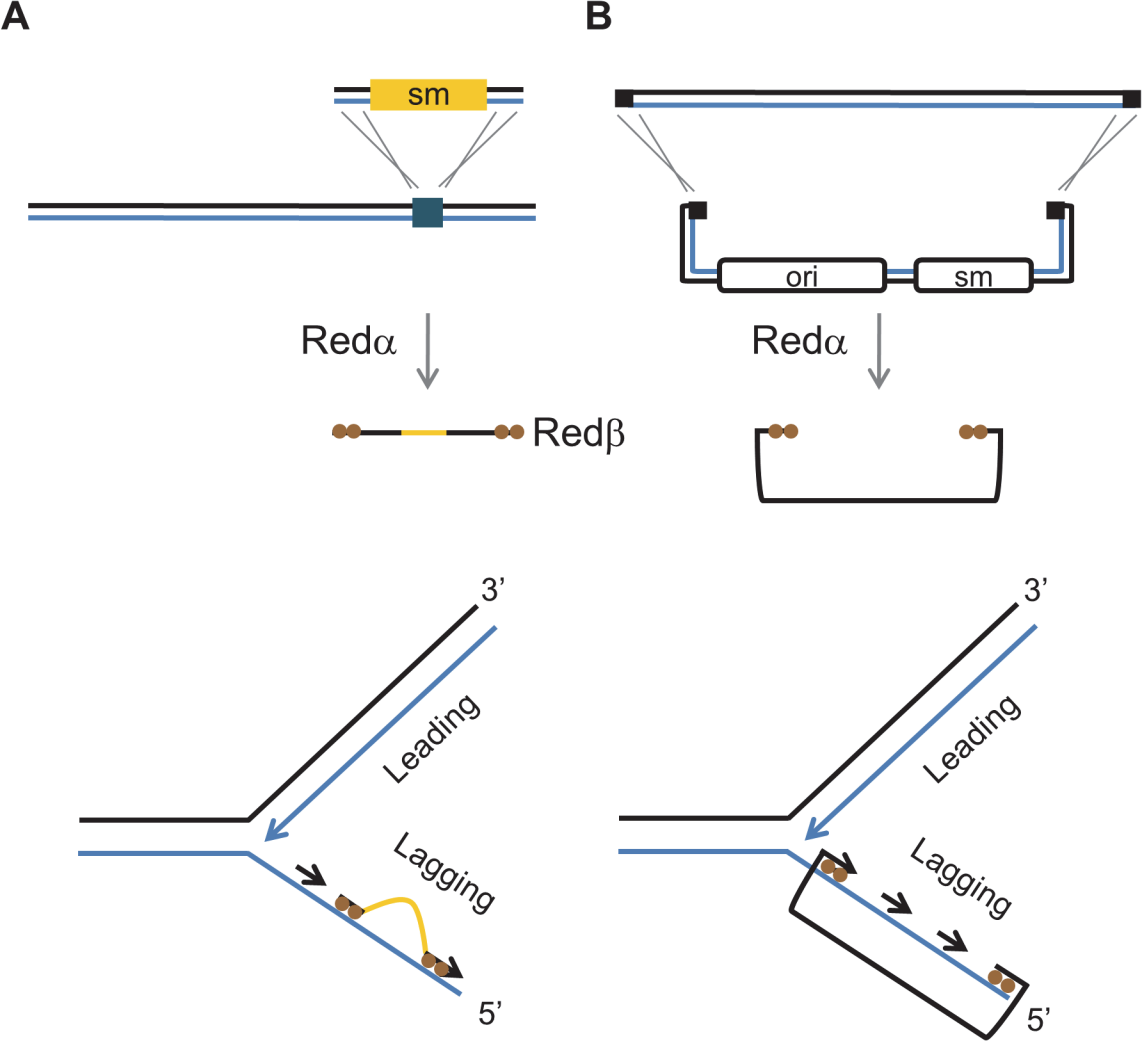
Model of Red mediated lagging strand recombination. (A) Lagging strand recombination. Redα digest of a double-stranded DNA cassette generates a single-stranded intermediate, which forms a heteroduplex (heterologous region in brown) on the lagging strand of the replication fork. (B) Lagging strand model of gap repair. A single-stranded plasmid is generated by Redα and anneals to the lagging strand of the replication fork. The intervening gap between the plasmid ends is filled through Okazaki fragment synthesis and the plasmid is recircularized. The circular ssDNA plasmid is then eventually converted to a dsDNA form by complementary DNA synthesis (not shown).

Recombineering (recombinogenic engineering) is a flexible and efficient genetic engineering technique that utilises bacteriophage recombination proteins to perform homologous recombination in the absence of host recombination functions [1–4]. Recombineering has become a central tool in mouse transgenics [5–7], bacterial metabolic engineering and synthetic biology [8–10], and is a cornerstone of recent whole genome recoding efforts [11]. Recombineering is primarily based on the bacteriophage λ Red operon, which encodes three proteins. Redα is a 5’-3’ exonuclease [12, 13] that acts cooperatively with Redβ, a single-strand annealing protein (SSAP) of the RAD52-RecT-Redβ family [14–18]. The third protein, Redγ is a DNA mimic that inhibits the *E*. *coli* RecBCD exonuclease and prevents degradation of linear double-stranded DNA (dsDNA) [19, 20]. Efforts to uncover the mechanism and explain the remarkable efficiency of recombineering led to the development of the beta recombination model [21–25]. According to this model, single-stranded oligos or linear dsDNA processed by Redα into single-stranded DNA (ssDNA) is bound by Redβ and annealed preferentially on the lagging strand of the replication fork (Fig. 1A). This replication dependent bias arises from the larger regions of ssDNA exposed by the replication fork on the lagging strand thereby allowing greater opportunity for annealing. Replication bias constitutes the central feature of the beta model and provides a mechanistic explanation for its high efficiency. Homologous pairs of exonuclease and single-strand annealing proteins from other phages show a similar strand directionality of recombination [25, 26], indicating that this mechanism of recombination is widely prevalent in prokaryotes [27–31].

Gap repair refers to the use of a linear plasmid containing terminal homology regions identical to a target to subclone almost any desired sequence [32, 33]. Recombineering mediated gap repair is a widely used process in the construction of gene targeting vectors and tagged plasmids [5, 6]. However, compared to insertional recombination (Fig. 1A) gap repair is not well understood [3, 24]. Gap repair in bacteria [34], yeast [35] and mammalian cells [36] is mediated through strand invasion of the plasmid ends and is consistent with the classical double-strand break repair (DSB) model of DNA recombination [37]. However, Red recombination in the absence of RecA and involving replicating molecules has been shown to primarily involve an annealing mechanism [23, 24, 38]. A lagging strand recombination model has been proposed to explain the mechanism of gap repair [24] (Fig. 1B), Here, we investigated the recombination mechanism of gap repair and compared it to lagging strand recombination. Consequently, we developed a novel multiplex dsDNA recombineering system. We found compelling evidence for the absence of lagging strand recombination in gap repair, which suggests that a novel replicative process leads to the closure of the gapped plasmid.

## Results

### Recombination assay system

We used a previously described system of asymmetrically modified dsDNA cassettes to investigate recombination events at the replication fork [23, 24, 31, 39]. Each cassette contained an antibiotic selection marker and additionally for subcloning plasmids, a p15A replication origin, flanked by ∼200 bp homology arms (HA) to the target site. Terminal 5’ phosphorothioate (PTO) bonds were used to protect one strand or the other against the Redα exonuclease. The complementary strand had a 5’ phosphate to promote its degradation by Redα and thereby release the ssDNA intermediate for annealing to DNA near the replication fork to either the lagging or leading strand. The PTO targeting constructs were verified *in-vitro* by Redα exonuclease digestion and showed the expected production of ssDNA (S1 Fig.).

### Gap repair lacks lagging strand bias

**Fig 2:**
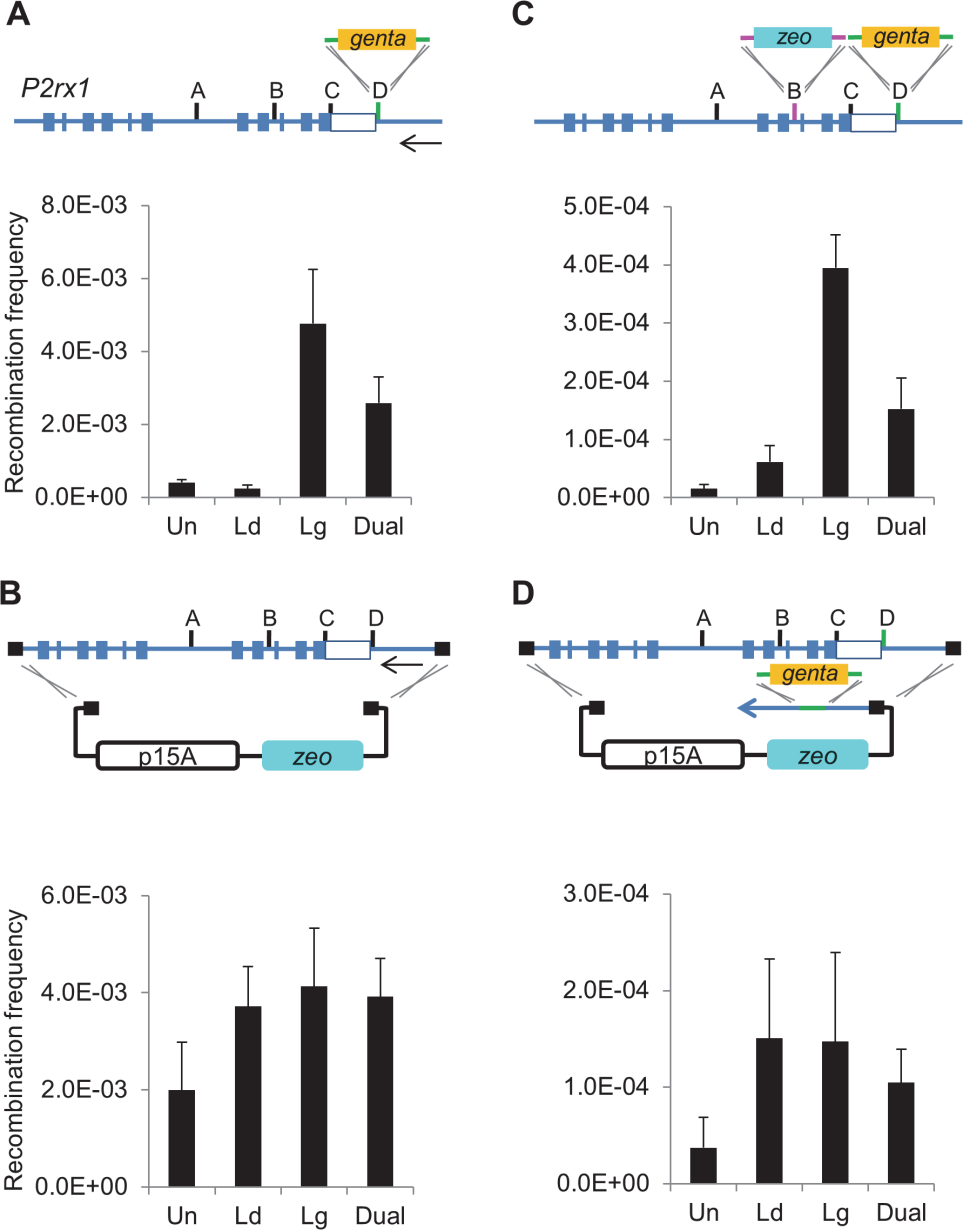
Gap repair does not exhibit a lagging strand recombination bias. (A) Insertion assay. The gbaA proteins were expressed and a differentially terminal modified Gentamicin cassette was transformed into the *P2rx1* BAC clone. Un, unmodified (hydroxylated); Ld, leading strand protected; Lg, lagging strand protected; Dual, both strands protected. The 12 kb segment of the *P2rx1* gene used in the insertion and gap repair assays is shown. Closed boxes represent exons (shown are exons 2–12) and the open box denotes the *P2rx1* 3’ UTR region. The different *P2rx1* targeting sites used in this study are shown as A-D. Arrow indicates the direction of replication fork movement. Histogram values represent averages; error bars indicate standard deviation (*n* = 3). The recombination frequencies plotted here and in the subsequent figures are included in S1 Table. (B) Gap repair assay. Gap repair was performed using p15A *zeo* asymmetric phosphorothioated plasmids. PCR genotyping and RE analysis revealed an average gap repair efficiency of 50% between replicate assays and between the different terminal modifications: Un, 41%; Ld, 63%; Lg, 63%; Dual, 50% (*n* = 72 for Un, Ld and Lg; *n* = 66 for Dual). Therefore, the total number of *zeo* resistant colonies were divided by 2 and reported as the gap repair frequency (*n* = 4). (C) Multiplex insertion. Two different Gentamicin and Zeocin cassettes were both differentially terminal modified in the same way and inserted at two different *P2rx1* sites with dual antibiotic selection (*n* = 3). (D) Subcloning plus insertion (SPI) assay. The p15A *zeo* subcloning plasmid and the Gentamicin cassette were both modified in the same way and co-selected in a combined recombination assay (*n* = 3).

Red mediated insertion is characterised by a bias towards lagging strand recombination. To evaluate the contribution of lagging strand recombination in gap repair, a gap repair assay with asymmetric PTO modifications was employed and compared to insertional recombination of a gentamicin cassette with asymmetric PTO modifications (Fig. 2A and 2B). Both assays employed the mouse *P2rx1* gene located on a bacterial artificial chromosome (BAC). As expected, insertion by lagging strand recombination was 20 fold greater than the leading strand (Fig. 2A and S1 Table). Colony PCR showed correct recombination in all cases (data not shown). In contrast, gap repair using asymmetric PTO modified plasmids showed no strand bias (Fig. 2B and S1 Table). Colony PCR and restriction digest (RE) analysis showed correct gap repair in half of the clones. The other were aberrant recombinants that contained an ∼800 bp fragment of the 3’ end of the *P2rx1* subcloned region (S9 Fig.). The efficiency of gap repair has been previously reported to vary at different loci (32, 33, 40). Consequently, gap repair assays were also performed at three other loci (*Chrm1*, *Dnttip1* and *Hdac1*) on different BAC clones (S2 Fig.). Gap repair at *Chrm1* (S2A Fig.) and *Hdac1* (S2C Fig.) showed slightly higher leading strand recombination (∼2 fold difference), while at *Dnttip1* the opposite was observed (S2B Fig.). The *Dnttip1* locus also yielded a much lower correct gap repair frequency (12–16%) and many aberrant recombinants. In contrast, the *Chrm1* and *Hdac1* loci yielded only correct inserts and no empty vectors. Overall, no consistent lagging strand bias was observed.

### Subcloning plus insertion (SPI)

Based on the evidence that gap repair employs a different recombination intermediate than insertional recombination, we speculated that the two processes could interact productively. To examine this idea, we compared double selection (zeocin and gentamicin) for a concerted gap repair plus insertion (subcloning plus insertion) with the same double selection for two insertion events. We call the first double selection assay ‘SPI’ for subcloning plus insertion. A great advantage of SPI is the selection against ‘empty vector’ background and selection for correctly gap repaired plasmids. This advantage applies even with sub-optimal homology lengths (compare S3A and S3B Fig.).

The two antibiotic resistance genes were terminally PTO modified on the same strand, either lagging or leading, and inserted into two different sites of the *P2rx1* gene resulting in a 6.4-fold bias in recombination towards the lagging strand (Fig. 2C). However, SPI produced no bias for the leading or lagging strands (Fig. 2D) again indicating a different mechanistic basis. PCR and restriction analysis confirmed correct recombination occurred in the majority of SPI cases and similar results were observed using a cassette targeting a different site of the *P2rx1* gene (S4 Fig.). However, PCR revealed that insertion of the two different selectable genes into the same BAC DNA was observed only with the lagging strand protected cassettes (S5A Fig.) whereas correct SPI products were observed with either strand protected (S5B Fig.). Plasmid mixtures in the same cell, characteristic of lagging strand recombination with circular multi-copy plasmids [41], were detected in only a few SPI clones. These dual recombination assays lend further support to the proposition that gap repair does not employ lagging strand recombination.

The efficiency of insertion has previously been shown to vary considerably across different genomic sites [21, 22, 24, 42]. Therefore, insertional recombination and SPI were tested at diverse sites across the *P2rx1* gene. As expected, insertion frequency varied greatly (> 7 fold) between the different sites (S6A Fig.). In contrast, SPI efficiency at the same sites was very similar (2 fold differences; S6B Fig.).

### SPI suppression of the lagging strand bias

**Fig 3:**
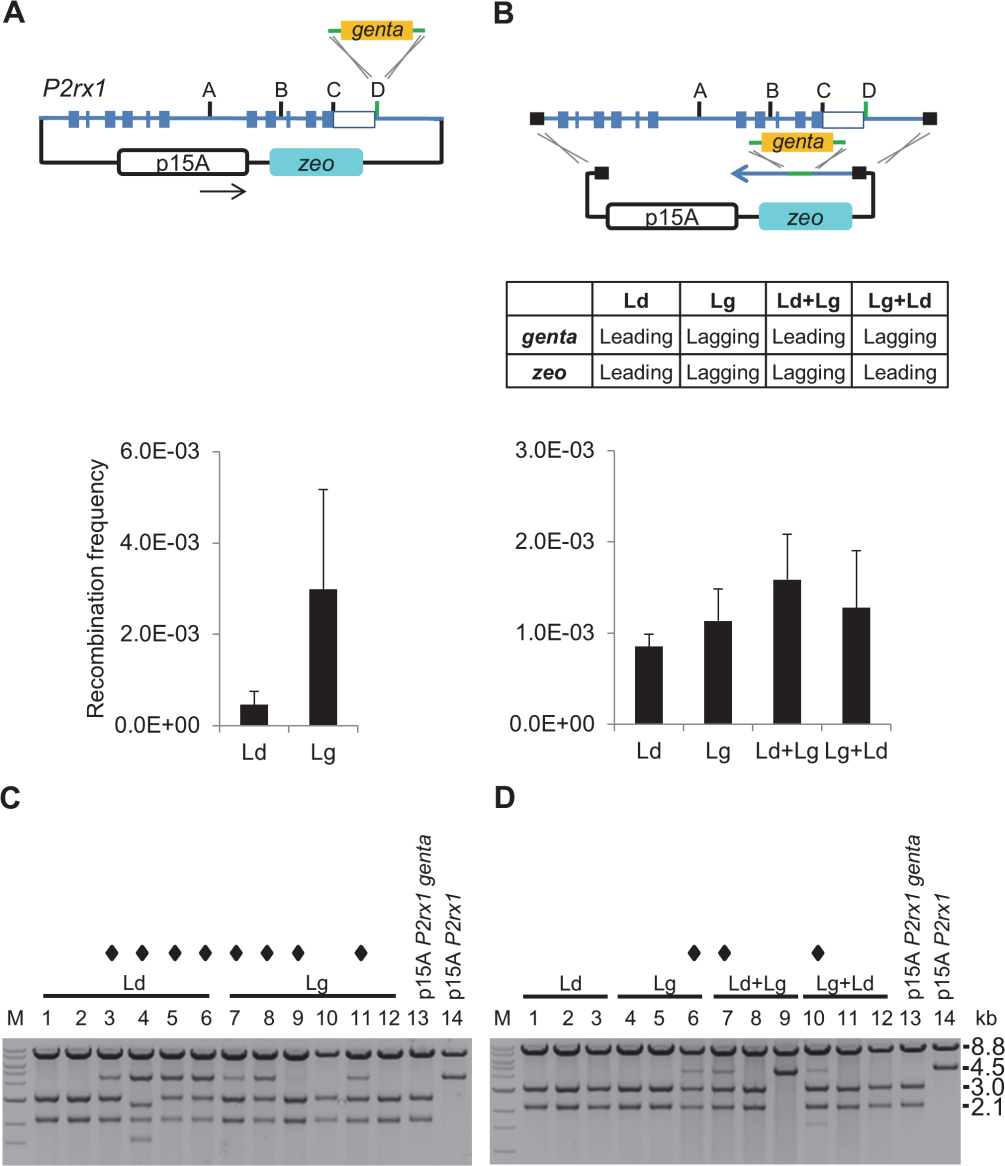
Effect of PTO positioning on SPI. (A) Co-transformation recombination assay. A p15A plasmid containing the 12 kb *P2rx1* gene segment shown in Fig. 2 was co-transformed with a Gentamicin insertion cassette into GB2005 cells induced for recombineering (gbaA) functions. Arrow indicates the direction of replication fork movement. Histogram values represent averages; error bars indicate standard deviation (*n* = 3). (B) SPI recombination assay. SPI assays were performed in parallel with the experiments shown in (A) and included different PTO combinations as shown in the table (*n* = 3). (C) Representative restriction digestion patterns of co-transformation recombinants. Plasmid DNA was digested with EcorV and SspI, and the resulting fragments were separated on a 1% agarose gel and visualizalised using ethidium bromide staining. M: 1kb ladder (NEB). Diamond symbol indicates clones containing targeted and non-targeted p15A plasmids. Restriction fragments sizes are (kb); p15A *P2rx1*, 8.8, 4.5; p15 *P2rx1 genta*, 8.8, 3.0, 2.1. (D) RE analysis of SPI recombinants. Restriction digests were performed as described in (C).

The SPI assay above was performed with the same PTO protected strand on both insertion cassette (gentamicin) and subcloning plasmid. To examine this issue in more detail, we repeated the experiment using PTO combinations (Fig. 3). First we verified that insertion of the gentamicin cassette into the subcloned region displayed the expected strand bias in this multicopy plasmid assay, as it did in the BAC assay (Fig. 3A). RE analysis showed most clones containing a mixture of targeted and unmodified plasmids in the same cell, as expected (Fig. 3C). In contrast, a SPI assay performed in parallel to subclone the same region from the *P2rx1* BAC using different PTO combinations produced similar recombination efficiencies (Fig. 3B). Notably, the insertional strand bias of the gentamicin cassette was diminished and RE analysis showed that the majority of the SPI recombinants contained a single, correctly recombined plasmid (Fig. 3D). The same SPI results were obtained using the plasmid replication origin in the other orientation (S7 Fig.).

### Reaction order and outcome of SPI

**Fig 4:**
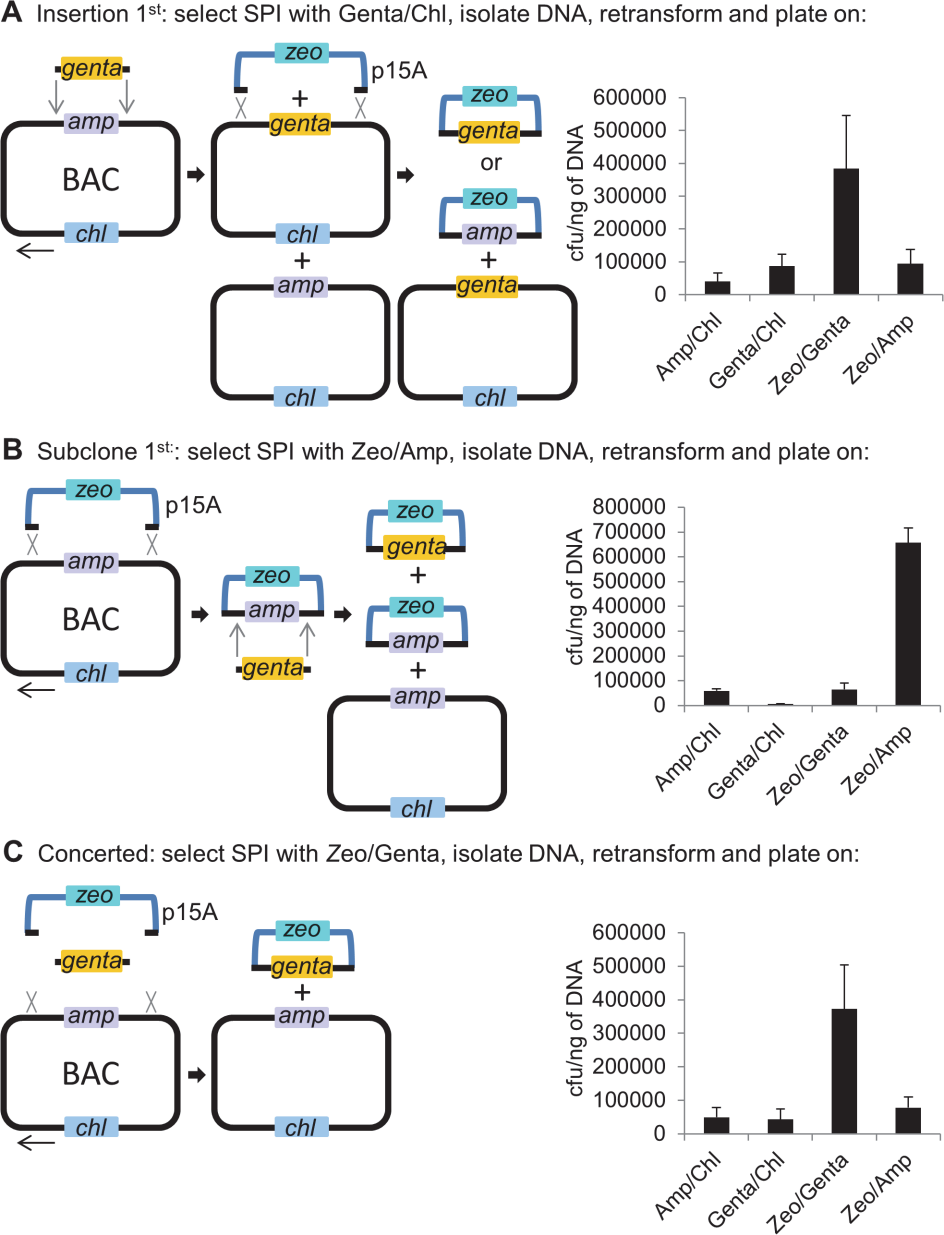
Scenarios of SPI recombination. The three possible routes of recombination in a SPI reaction are shown. (A) Consecutive route 1; first insertion of a cassette into the BAC followed by gap repair. (B) Consecutive route 2; first gap repair from the BAC followed by cassette insertion into the gap repaired plasmid. (C) Concerted gap repair and cassette insertion into the subcloning plasmid. The different recombination routes are not mutually exclusive. A pBeloBAC11 plasmid containing the 12 kb *P2rx1* region shown in Fig. 2 was modified with insertion of an *amp* marker atsite D to generate the 19.3 kb BAC plasmid for the SPI experiments. Arrow indicates the direction of replication fork movement. SPI recombination was performed for 15 mins with gbaA proteins and lagging strand cassettes. Equal amounts of the cells were then plated on different pairs of antibiotics to select for each recombination route. The colonies were washed off the plates and plasmid DNA was prepared using a miniprep kit. 1 ng of plasmid was transformed into HS996 cells and equal amounts of a serial dilution were then plated on different pairs of antibiotics. Low concentrations of antibiotics were used to allow comparison of the different selection schemes. The resulting colony counts are presented as histograms. Values represent averages; error bars indicate standard deviation (*n* = 3).

The loss of insertional strand bias in SPI raised questions regarding the possible order of recombination. Three cases are possible: A) insertion of the cassette onto the BAC first followed by subcloning, B) gap repair of the subcloning plasmid first followed by cassette insertion and, C) concerted subcloning and cassette insertion (Fig. 4). Each case will produce the same final plasmid, however the intermediates differ. To discriminate between the different cases, the SPI reaction was first plated on the three different pairs of antibiotics used for selection. From each of these plates, plasmid DNA was prepared, transformed again and plated onto four different combinations of antibiotics representing each of the different possible recombination intermediates.

If insertion precedes subcloning, then a gentamicin resistant BAC will be generated but not if subcloning precedes insertion or during a concerted reaction. Therefore Gentamicin/Chloramphenicol selection should favour the identification of insertion-first events. However Gentamicin/Chloramphenicol colonies after the second selection constituted only about 15% of total colonies whereas the intended Zeocin/Gentamicin product constituted about 2/3rds of total colonies (Fig. 4A). A very similar profile was obtained after first selection for the intended Zeocin/Gentamicin product (Fig. 4C). In contrast, first selection for Zeocin/Ampicillin revealed predominance of the subclone-first intermediate (Fig. 4B). The appearance of the other postulated recombination intermediates indicates that multiple recombination routes can operate in a SPI reaction. However in Fig. 4A and 4C, the approximately five-fold predominance of the Zeocin/Gentamicin SPI product, which depends on two recombination events, over the Zeocin/Ampicillin subclone product, which depends on only one of those events, again indicates the operation of a concerted mechanism.

### Directly testing ssDNA shows gap repair does not involve lagging strand recombination

**Fig 5:**
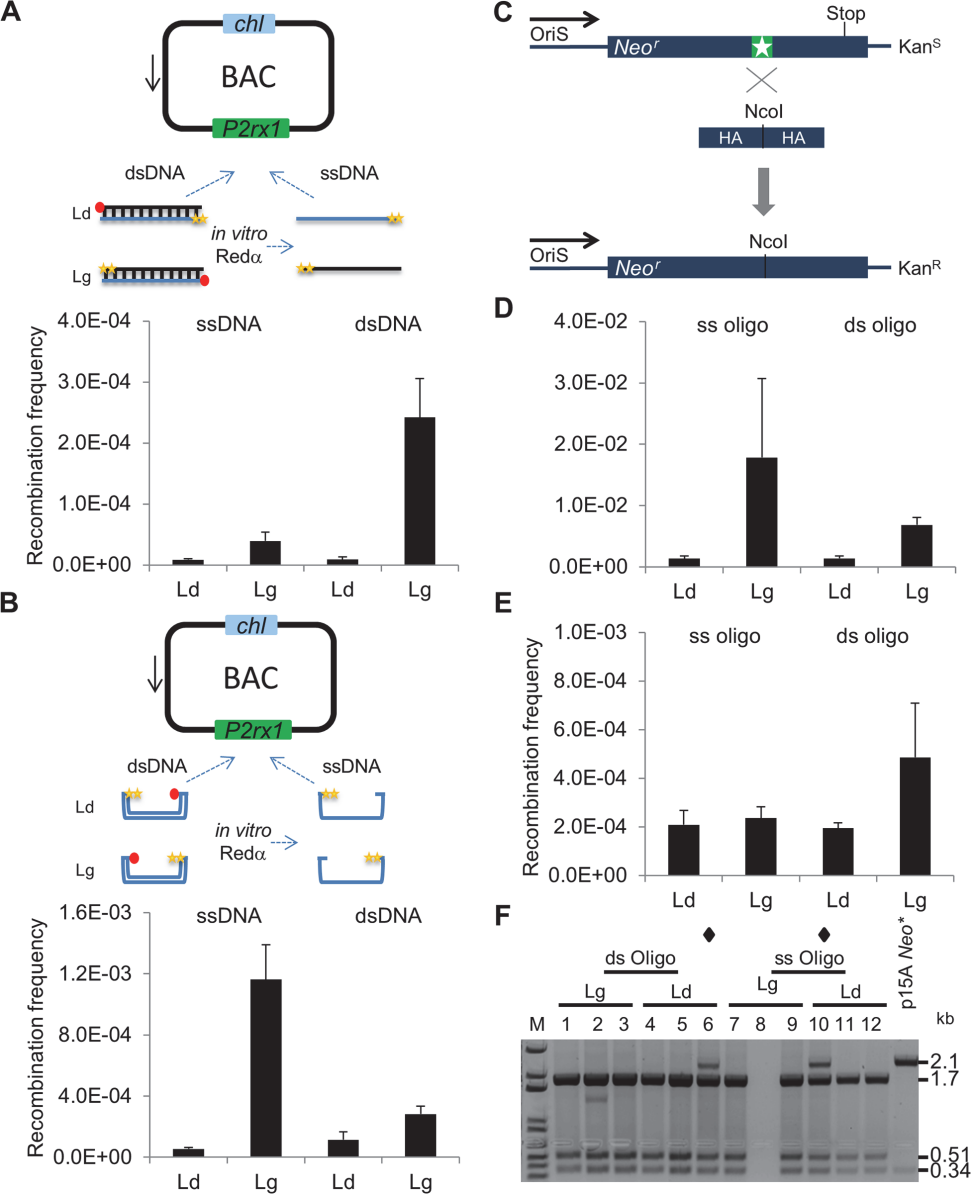
Redβ assays reveal the absence of lagging strand recombination during gap repair. (A) Redβ insertion assay. Terminal modified ssDNA and dsDNA Zeocin cassettes targeting *P2rx1* site D were transformed into cells expressing only Redβ. The yellow star symbol indictates a phosphorothioate modification and the red closed circle indicates a phosphate modification. Histogram values represent averages; error bars indicate standard deviation (*n* = 3). (B) Redβ gap repair assay. Terminal modified ssDNA and dsDNA p15A *zeo* plasmids were used with Redβ expression. Corrected gap repair frequency, as described in Fig. 2B, is shown (*n* = 4). (C) Scheme of oligo rescue of a BAC clone defective for the Tn5 Neomycin (*neo*) marker. The asterisk indicates a 4 bp insertion at the NcoI site of the *neo* gene that introduces a frameshift and thus a premature stop codon, leading to kanamycin sensitivity. Transformation of the correcting oligo (HA, homology arm) deletes the 4 bp insertion and restores kanamycin resistance. The direction of replication fork movement from OriS is shown. Oligo rescue experiments were performed with the gbaA proteins. (D) ssOR assay. Terminal modified ssDNA and dsDNA rescue oligos were used (*n* = 3). (E) SPI ssOR assay. SPI ssOR was performed using the p15A *zeo* dsDNA plasmid and terminal modified ssDNA and dsDNA rescue oligos (*n* = 3). (F) RE analysis of the SPI ssOR assay. Plasmid DNA was prepared from SPI ssOR recombinants and digested with NcoI and DraIII. The empty lane (lane 8) represents a lost sample. M: 1kb+ ladder (Invitrogen). Star symbol indicates clones containing targeted and non-targeted p15A plasmids. Restriction fragments sizes are (kb); p15A neo*, 2.12, 0.34; p15 *neo*, 1.76, 0.51, 0.34.

The Red mediated recombination of an ssDNA substrate with its target sequence requires only the presence of Redβ [21, 25]. Therefore, transformation of ssDNA into *E*. *coli* cells expressing only Redβ can be used to assess lagging strand recombination directly, albeit with reduced efficiencies compared to dsDNA substrates and Redαβγ [23, 24]. To test if gap repair can be mediated by lagging strand recombination, we performed recombination assays with both ssDNA and dsDNA in *P2rx1* BAC *E*. *coli* cells expressing only Redβ. PTO protected leading or lagging strand *zeo* insertion cassettes and p15A subcloning plasmids were digested *in-vitro* with Redα to generate single stranded products. Although secondary structures of the ssDNA insert possibly impede recombination [8, 24] (here about 6 fold lower than that of the corresponding dsDNA cassette), recombination with both ssDNA and dsDNA cassettes was clearly lagging strand biased (4.6 fold and 27.1 fold greater than the leading strand ssDNA and dsDNA, respectively; Fig. 5A). Gap repair with ssDNA and dsDNA p15A plasmids showed that subcloning with ssDNA is possible and displayed a lagging strand bias. The lagging strand ssDNA plasmid was 21.5 fold more recombinogenic than the equivalent leading strand plasmid (Fig. 5B). In contrast, gap repair with the corresponding dsDNA vectors exhibited only a small lagging strand bias, again indicating that Red mediated gap repair in *E*. *coli* utilises a dsDNA intermediate that is not explained by lagging strand recombination. Gap repair and SPI assays were also performed in Redγβα and RecA (gbaA) [43] expressing cells using ssDNA and dsDNA constructs and showed a similar lack of strand bias (S8A and S8B Fig.) supporting our claim of a different gap repair pathway.

Single-stranded oligo repair (ssOR) is another way to directly evaluate lagging strand recombination. (23). Using a previously described Neo* BAC system [25, 44], a defective kanamycin resistance gene is repaired by lagging or leading strand ssDNA or asymmetric PTO-protected dsDNA oligos (Fig. 5C). As expected, ssOR using both ssDNA and dsDNA oligos was more efficient on the lagging strand (ssDNA, 12.9 fold; dsDNA, 4.83 fold; Fig. 5D). In contrast, performing simultaneous ssOR in a SPI reaction showed little difference in ssOR efficiency between the lagging strand and leading strand (Fig. 5E). DNA sequencing (data not shown) and restriction digests (Fig. 5F) of SPI ssOR clones revealed restoration of the interrupted NcoI site and additionally showed most of the clones contained only a single plasmid. Comparison of ssOR to dsDNA insertion (compare Figs. 2A and 5D) revealed a 4 fold increase in recombination that was not not observed with SPI (compare Figs. 2D and 5E). However, the gap repair efficiency of the p15A Neo* subcloning plasmid was ∼10^–3^ and SPI produced a 10 fold decrease in recombination efficiency that was expected with multiplexing.

The completion of lagging strand DNA synthesis involves Okazaki fragment maturation, which in *E*. *coli* is performed by DNA polymerase I (PolI) [45]. Impaired PolI activity has been shown to affect lagging strand recombination directly [46, 47] and leading strand recombination indirectly through polymerase uncoupling [46, 48]. We examined the effect of deleting the PolI 5’-3’ exonuclease domain, which is responsible for Okazaki RNA primer replacement, on SPI ssOR compared to lagging strand recombination using the Neo* BAC system. In agreement with previous results [46, 47], ssOR with the lagging or leading strand ssDNA oligo was greatly reduced (> 25 fold) in the XTL85 PolI mutant strain, compared to the PolI wt HME 68 strain. In contrast, a slight increase (> 3 fold) in SPI ssOR frequency was observed in XTL85 relative to HME 68 (Table 1). The HME68 and XTL85 strains are deficient in the *mutS* methyl mismatch repair system (*mutS*::*cat*) that contributes to the differential recombination efficiency between the lagging strand and leading strand. In agreement with a previous study [23], we observed an absence of lagging strand ssOR bias at the neo* BAC locus in HME68 and XTL85 strains (Table 1). Overall, the data from ssDNA experiments supports the observation of a lack of preferential lagging strand recombination during gap repair.

**Table 1:** Oligo rescue experiments in DNA Polymerase I defective recombineering strains.

	ssOR assay^a^			
	ssOR		SPI ssOR^b^	
	ssOR frequency^c^			
Strains	HME 68	XTL85	HME 68	XTL85
Leading	4.55E-01	2.02E-02	1.31E-05	4.92E-05
Lagging	4.13E-01	1.80E-02	1.09E-05	4.30E-05

^a^The Neo* BAC was modified to express the R6K *pir*+ gene.

^b^SPI ssOR was performed with a dsDNA R6Kγ *zeo* subcloning plasmid and ssDNA oligos.

^c^The total number of antibiotic resistant colonies was divided by the total number of viable cells. Values represent averages of three experiments. Variation between assays was nearly 4 fold.

### Greater ends-out recombination during SPI

**Fig 6:**
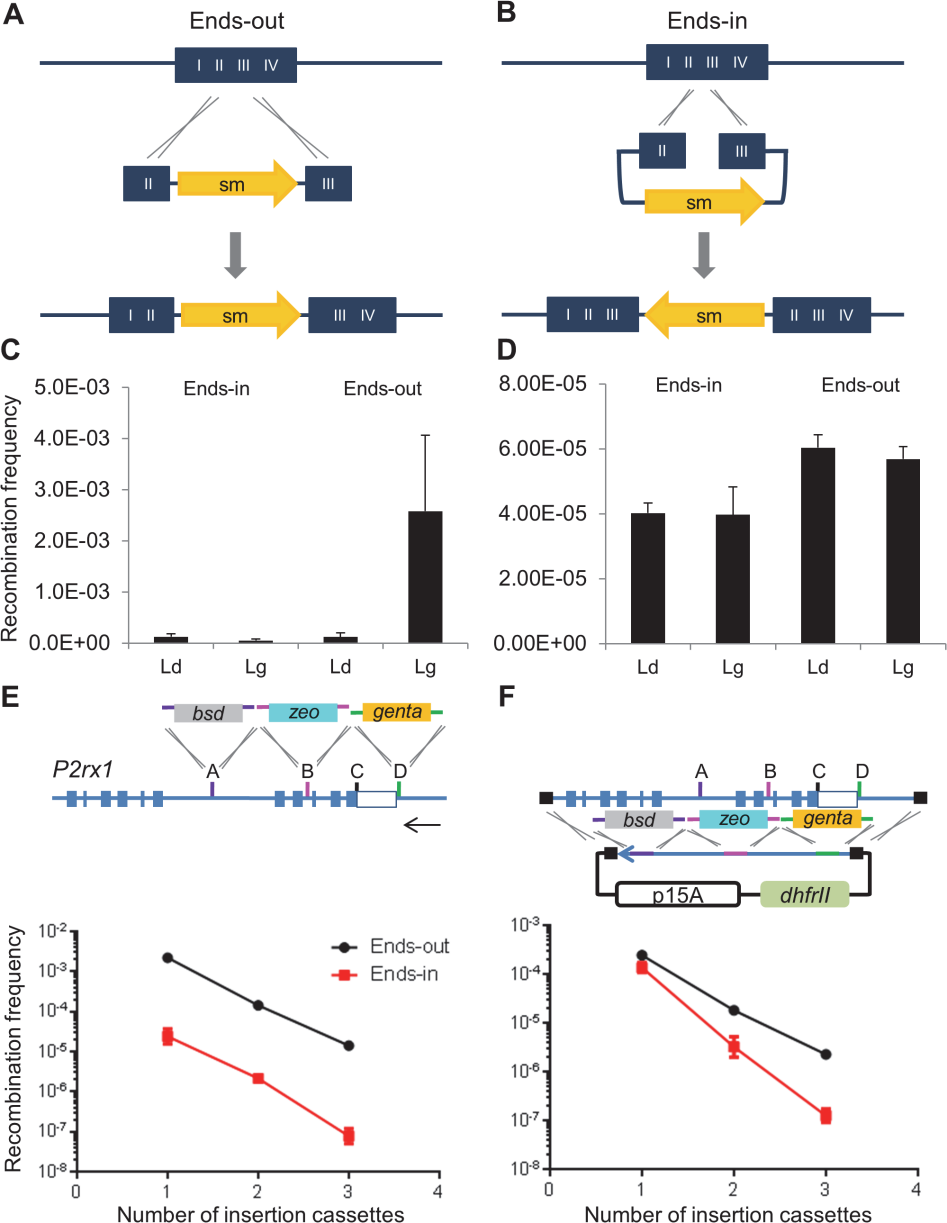
Both SPI and multiplex beta recombination show preferential ends-out recombination. (A) Ends-out recombination. (B) Ends-in recombination. HA, homology arm; sm, selection marker. (C) Insertion assay. The gbaA proteins were expressed and ends-out and ends-in Gentamicin cassettes were inserted at *P2rx1* site D. Histogram values represent averages; error bars indicate standard deviation (*n* = 3). Ld, Leading strand protected; Lg, Lagging strand protected. (D) SPI using ends out and ends-in Gentamicin cassettes (*n* = 3). (E) Multiplex insertion. Different antibiotic resistance cassettes with homology regions in an ends-out or an ends-in configuration were inserted across different *P2rx1* sites. Lagging strand cassettes were used in combination as follows. 1 cassette, Gentamicin; 2 cassettes, Gentamicin and Zeocin; 3 cassettes, Gentamicin, Zeocin and Blasticidin. Arrow indicates the direction of replication fork movement. The multiplex insertion frequency is shown (*n* = 3). (F) SPI recombination assay. SPI was performed using the ends-out or ends-in cassettes described in (E). The SPI recombination frequency is plotted below (*n* = 3).

The insertion of a dsDNA cassette involves annealing of the ssDNA intermediate to the lagging template strand, with the terminal homology regions in an ends-out configuration (Fig. 6A). When the same homologies are reverse complemented (ends-in configuration), the cassette recombines via a different pathway [23] that leads to the duplication of the homology regions (Fig. 6B). Ends-in recombination is less efficient than ends-out and also demonstrates no lagging strand bias (Fig. 6C). In contrast, ends-in and ends-out insertional recombination using SPI were similarly efficient without strand bias (Fig. 6D). To explore this result in greater detail, we co-selected for simultaneous ends-out or ends-in events at different *P2rx1* sites with and without SPI. As expected, fewer recombinants were produced with increasing cassette insertions from 1 to 3 in either insertional or SPI assays. Both sets of assays also showed more efficient ends-out recombination than ends-in recombination (Fig. 6E and 6F). PCR analysis of the BAC clones revealed that concurrent multiplex insertion on the same BAC DNA was observed only with the ends-out cassettes, while the ends-in cassettes produced BAC clone mixtures (data not shown). In contrast, SPI produced correct multiplex insertion of both the ends-out and ends-in cassettes in most of the clones analysed and plasmid mixtures were rarely detected (data not shown). SPI assays were also performed with each of the three ends-out and ends-in cassettes separately. Although, the ends-in cassettes showed similar SPI recombination frequencies between the different *P2rx1* sites, they were less efficient (4 fold) than the ends-out cassettes at each site (S10 Fig.)

Finally, recombination assays were performed in *ruvABC E*. *coli*, which is deficient in Holliday junction resolution. Insertion (S11A Fig.), gap repair (S11B Fig.) and SPI (S11C Fig.) assays showed similar recombination frequencies between WT and *ruvABC* strains (2 fold differences), suggesting that all three recombination reactions do not appear to invoke a Holliday junction [23]. While the recombination assays described here utilised transient expression of RecA to improve cell viability post electroporation, the absence of RecA did not affect recombination under similar experimental conditions in other experiments (Reddy *et al*., under review), supporting an annealing mode of recombination.

### SPI with multiple cassettes

To test the utility of SPI in vector construction strategies, up to 4 different inserts were recombined in a single SPI reaction (S12A Fig.). Larger BAC subcloning plasmids were also used to perform efficient SPI cloning (S12B Fig.).

## Discussion

Recombineering mediated gap repair is a primary DNA engineering tool because it permits efficient subcloning of a target region with nucleotide precision. Previous recombineering gap repair studies sought to improve the methodology and the mechanistic aspects were not investigated [40, 49, 50]. The DBSR model has been proposed for Red mediated gap repair [51–54], but is based on studies performed in RecA+ strains and using repair templates that were not actively replicating. The reported activity of Redβ to mediate strand invasion into a duplex DNA in vitro [55] is not corroborated by in vivo data from replication dependent experimental settings, which primarily support a single-strand annealing mechanism of Red recombination [38, 56]. Indeed, recent studies [22–24] have provided evidence for the view that lagging strand recombination is the major “recombinational repair transaction” [57] that Red system performs on the chromosome. Based on this model, Church and colleagues postulated a lagging strand mechanism of gap repair that could involve Okazaki fragment mediated gap filling [24]. In this study, we have shown that Red mediated gap repair is a novel homologous recombination pathway in *E*. *coli* distinct from lagging strand recombination.

Although we showed that an ssDNA version of a plasmid could subclone via gap repair and showed a lagging strand bias, double stranded plasmids do not employ this mechanism because lagging strand bias was not observed during gap repair. Furthermore, oligo recombination during SPI also did not show a lagging strand bias and did not require PolI activity, both hallmarks of lagging strand recombination [46, 47]. The annealing of an ssDNA to the lagging strand of the replication fork generates a heteroduplex intermediate [23, 24] that is subsequently resolved by a second round of replication into the targeted allele and a restored WT allele [23]. However, SPI preferentially generated only the targeted allele, even with ssDNA, which requires a different explanation. Maresca and colleagues demonstrated an alternate ends-in recombination pathway of Red recombination but the lower efficiency of ends-in recombination during SPI argued against this mechanism. The template switching model [56] proposes a different route of dsDNA integration but is unlikely to operate in our multiplex assay system. Red mediated gap repair is also distinct from plasmid gap repair involving linear non-replicating templates [10, 58, 59] because it depends on active replication [60]. Overall, these data suggest that gap repair could either utilize an alternate mode of DNA replication different from the known canonical replication fork or employ an unknown recombinational mechanism.

We exploited our finding of a different mechanism for gap repair to develop SPI, which is a novel recombineering application based on simultaneous gap repair and insertional mutagenesis. This multiplex application is surprisingly efficient, possibly because it employs the co-selection principle [61, 62] or a concerted mechanism. SPI is particularly useful because empty plasmid recircularization due to aberrant, intramolecular recombination [22] is eliminated. The concerted selection for insertion whilst subcloning simplifies the construction of multi-component vectors such as targeting constructs and tagged protein expression plasmids [63]. Practical improvements in recombineering have previously been achieved through investigation of the mechanistic aspects of lambda Red recombination [23, 24, 42]. Similarly, we identified Red mediated gap repair as an efficient recombination mechanism different from lagging strand recombination, which has practical utility. Exploration of the mechanistic basis of gap repair may provide further insights and improved recombineering strategies.

## Materials and Methods

### Genetic loci and insertion sites

Recombineering assays were performed primarily at the mouse genomic locus spanning the *P2rx1* and *Camkk* genes on the RP24-360O20 BAC clone. Gap repair assays were additionally performed at the mouse *Chrm1* (RP23-7I17), *Dnttip1* (bmq298-g11) and *Hdac1* (RP23-229F19) loci. The oligo rescue experiments were performed with the Neo* BAC clone (RP23-337H1), which was described before (25). All the BAC libraries were constructed in the RecA deficient *E*. *coli* DH10B strain (*E*. *coli* genotype: F– *mcr*A Δ(*mrr*-*hsd*RMS-*mcr*BC) Φ80*lac*ZΔM15 Δ*lac*X74 *rec*A1 *end*A1 *ara*D139 Δ(*ara leu*) 7697 *gal*U *gal*K *rps*L *nup*G λ).

Four different sites at the *P2rx1* gene were selected for the insertion of the antibiotic resistance markers. Two intronic sites (sites A and B) were compared with a site spanning the *P2rx1* 3’UTR (site C), and a site located in the intergenic space at the end of the 3’UTR (site D).

### Recombineering plasmids, strains and oligos

BAC clones were transformed with temperature sensitive pSC101 plasmids containing an arabinose inducible promoter for transient expression of the Redγβα proteins and the RecA protein [43, 64], or only the Redβ protein [65]. The recombineering proficient *E*. *coli* strains, XTL85, containing the *polA* 5’-3’ exonuclease deletion mutant, and HME68, the *polA* wild type (wt) strain [46] were obtained from Donald L. Court, NCI, U.S.A. The RuvABC deletion strain was constructed by replacing *ruvAB* with an *amp* marker and *ruvC* with an *rpsL-bsd* cassette using standard recombineering methodology [64]. Standard or modified oligos were purchased from Invitrogen and IDT as desalted except for the oligo rescue assays where they were polyacrylamide gel electrophoresis (PAGE) purified. Asymmetric phosphorothioated (PTO) double stranded oligos (IDT) were further purified with the minElute PCR purification kit (Qiagen) and quantified by measuring their 260 nm absorbance with a Nanodrop spectrophotometer (ND1000, Thermo Scientific).

### Insertion cassettes and subcloning plasmids

The homology regions of the insertion cassettes and subcloning plasmids were cloned into R6Kγ and p15A plasmids, respectively using Infusion cloning (Infusion HD clonit kit, Clontech) and recombineering. A TN3-*dhfrII* antibiotic resistance cassette [66] was used in the p15A plasmid, which offers a more robust selection than the wt *dhfrII* version. The subcloning plasmids were linearised at a unique NotI (NEB, UK) site introduced between the two homology regions. Insertion cassettes and subcloning plasmids were PCR amplified using the KOD PCR system (Millipore Merck). Each PCR reaction included 1 x KOD PCR buffer, 200 μm dNTPs, 1.5mM MgSO_4_, 1.3 M Betaine (Sigma), 1% DMSO (Sigma), 200 nM primers, 1U KOD DNA polymerase and 10–50 ng of template DNA. Thermal cycling was performed using the following conditions: 95°C, 2 mins; 35 cycles of 92°C for 10secs, 55°C for 30 secs, 72°C for 30 secs. PCR products were column purified using the MinElute PCR purification kit. Subcloning plasmids were additionally then treated with DpnI (NEB) overnight and again column purified. The sequences of the insertion cassettes and subcloning plasmids are shown in S2 Table. The plasmid and strain construction and genotyping oligo sequences are available on request.

### ssDNA constructs

ssDNA constructs were prepared by *in-vitro* Redα digest of the asymmetric phosphorothioated linear dsDNA cassettes. Each digest contained 10 μg of the dsDNA cassette and 50 units of λ-exonuclease (NEB) in a total volume of 200 μl. The reaction was incubated at 37°C for 30 minutes and then immediately purified using the minElute PCR purification kit. The ssDNA fraction was quantified by measuring the absorbance at 233 nm on a Nanodrop spectrophotomter. Equimolar amounts of ssDNA (600 ng) and dsDNA (1, 200 ng) were used in the Redβ and gbaA assays.

### Oligo rescue system

Single-stranded oligo rescue (ssOR) [25] was tested using a previously described system with a defective Neo marker (Neo* BAC). The sequence of the lagging and leading strand rescue oligos is shown in S2 Table. PTO modified and PAGE purified oligos were employed in the ssOR assays [67]. In each experiement, 600 ng of the single-stranded oligos and 1, 200 ng of the double-stranded oligos and 1, 200 ng of the dsDNA subcloning plasmid was used. Additionally, the Neo* BAC was retrofitted with a R6K *pir+* gene linked to a *genta* marker, which replaced the BAC *chl* marker, to support the replication of the R6Kγ origin subcloning plasmid in the PolI strains.

### Standard recombineering protocol

Recombineering was routinely performed with 10 ml cultures per sample using a previously described protocol [64] with the following modifications. Following an overnight growth, the BAC culture was diluted 50 fold in Lysogeny Broth (LB) pH 8 containing antibiotics and grown shaking at 30°C to an optical density (OD) OD_600_ of ∼0.3. Recombineering functions were induced by the addition of Arabinose (Sigma, UK) to 0.2% final concentration and the cultures were shifted to 37°C shaking for 45 minutes. The cells were washed 3 times in cold 10% glycerol and electroporated with DNA using a setting of 1.8 kv, 200 Ω and 25 μF with a Gene Pulser electroporation apparatus (Bio-Rad). The cells were recovered immediately in 950 μl of LB pH 8 and grown shaking at 37°C for 1 hour, except the recombination assay in Fig. 4, which was incubated for only 15 minutes. The pBeloBAC11 samples (S11B Fig.) were recovered in 10 ml of LB in a Erlenmeyer flask and grown for 4.5 hrs at 37°C. Serial dilutions of the cells were made in Tris-Magnesium Sulphate-Gelatin (TMG) buffer and plated on LB pH 8 agar plates containing either the selective antibiotics or lacking any antibiotics to obtain the total viable cell count.

Since the PolI strains contained the defective λ-prophage, a heat shock procedure was performed to induce Red expression from the *P*_*L*_ operon [68]. An overnight culture of the HME 68 strain was diluted 80 fold and the XTL85 strain was diluted 30 fold and grown to an OD_600_ of 0.5. The cultures were then transferred to a shaking water bath (200 rpm) at 42°C for 15 min. Heat shocked cells were immediately chilled in an ice slurry with shaking for 5 minutes followed by the washing and electroporation steps as above. The PolI cultures were recovered at 30°C for 120 minutes and plated on the appropriate selective agar plates.

The following antibiotic concentrations were used in plates: 50 μg ml^-1^ Ampicillin, 40 μg ml^-1^ Blasticidin, 12.5 μg ml^-1^ Chloramphenicol, 2 μg ml^-1^ Gentamicin (except in Fig. 4 where 1 μg ml^-1^Gentamicin was used), 30 μg ml^-1^ Hygromycin, 15 μg ml^-1^ Kanamycin (except 6.5 μg ml^-1^ when used in combination with Gentamicin in S12A Fig.), 4 μg ml^-1^ Tetracycline, 10 μg ml^-1^ Trimethoprim, 5 μg ml^-1^ Zeocin. The same antibiotic concentrations were used in liquid culture except Gentamicin, which was used at 1 μg ml^-1^.

### PCR and restriction analysis

A four-step validation strategy was employed to analyze the recombinants. To detect the insertion of the antibiotic cassettes and to check for gap repair cloning, a high throughput PCR screen was performed using one homology region flanking primer and one insert specific primer. Between 8 and 48 colonies were picked per sample per assay into 200 μl of LB+antibiotics in a 96-well plate and grown at 37°C overnight. Colony PCR genotyping was performed using 2 μl of the saturated culture, 1 μM of each primer and 0.97x ReddyMix (Thermo, UK) in 20 μl total volume. PCR was performed using the following conditions: 95°C, 15 mins; 35 cycles of 92°C for 10secs, 55°C for 30 secs, 72°C for 45 secs; 72°C for 10 mins. Plasmid DNA was prepared from 3 to 12 insert containing clones per sample using the QIAPrep spin miniprep kit or using a BAC miniprep protocol [69]. Long range PCR’s were performed using the KOD PCR system and insert (antibiotic cassette) flanking primers, located outside the homology regions. Thermal cycling conditions were: 95°C, 2 mins; 35 cycles of 92°C for 10secs, 55°C for 30 secs, 72°C for 30 secs. Gap repair and SPI assays were further analysed with RE analysis of at least 3 clones per sample per assay. DNA sequencing was performed across the Neomycin insert junction of 3 clones per sample to verify the correct oligo targeting event in the SPI ssOR experiments. Agarose gel images were processed in Adobe Photoshop to invert the image. All images were adjusted in Microsoft Powerpoint to obtain-40% brightness and +40% contrast.

### Colony Counts and statistical analysis

The total number of antibiotic resistant colonies was determined for each sample and divided by the total number of viable cells surviving each electroporation event to calculate the total recombination frequency. Mean and standard deviation values were calculated from multiple independent replicates (see individual figures for details). The colony counts of gap repair assays were adjusted for background recombination frequency (empty vector and aberrant recombinants) as described in the figure legends. Statistical analyses were performed in GraphPad Prism using a one-tailed *t*-test assuming unequal variances. A significant difference between samples was considered at *p* < 0.05.

## Supporting Information

S1 Fig *In-vitro* Redα digest of terminal modified linear DNA cassettes. (A) Expected patterns of Redα digest of terminal modified DNA cassettes. The different terminal modifications included, Un, unmodified (hydroxylated); Ld, leading strand protected; Lg, lagging strand protected; Dual, both strands protected. (B) Redα digest of the Gentamicin insertion cassette targeting *P2rx1* site D (1.0 kb). (C) Redα digest of the p15A *zeo P2rx1* subcloning plasmid (1.7 kb). Each digest contained 1 μg of the dsDNA cassette and 5 U of λ–exonuclease (NEB). Control samples contained 200 ng of DNA and did not include exonuclease. The samples were analysed by agarose gel electrophoresis and ethidium bromide staining. Images were inverted and the contrast was improved (see methods). M, 1 kb+ ladder (Invitrogen); λ, Lambda HindIII digest (NEB).
(TIF)

S2 Fig Gap repair at different genomic loci reveals the absence of lagging strand recombination. Gap repair assays were performed at three different mouse genomic loci on different BAC clones using gbaA expression and asymmetric phosphorothioated p15A *zeo* plasmids. (A) *Chrm1*. (B) *Dnttip1*. (C) *Hdac1*. Closed boxes represent exons and the open box represents the 3’UTR region. Arrow indicates the direction of replication fork movement. The *Chrm1*, *Dnttip1* and *Hdac1* gap repair insert sizes were 12 kb, 12.6 kb and 9.1 kb, respectively. Histogram values represent averages; error bars indicate standard deviation (*n* = 3). Ld, Leading strand protected; Lg, Lagging strand protected. Gap repair frequency at *Dnttip1* was calculated using colony PCR genotyping (*n* = 24). The total number of recombinants at *Chrm1* and *Hdac1* is directly reported as the actual gap repair frequency since correct gap repair was observed in all the clones analysed for both loci (*n* = 24 each for Ld and Lg). A *t*-test did not show any significant differences between leading and lagging strand recombination: *Chrm1*, *p* = 0.1000; *Dnttip1*, *p* = 0.1000; *Hdac1*, *p* = 0.0500.
(TIF)

S3 Fig SPI selects for correct gap repair. (A) Gap repair using short homologies. Gap repair was performed using gbaA proteins and a p15A lagging strand protected plasmid containing 50 bp homology regions. The recombinants were analysed by RE digests using KpnI. Arrow indicates the direction of replication fork movement. M, 1 kb+ ladder (Invitrogen). Restriction fragments sizes are (kb); p15A *P2rx1*, 6.9, 3.7, 2.6. (B) Subcloning plus insertion (SPI) using short homologies. SPI was performed using a p15A *zeo* subcloning plasmid and a Gentamicin lagging strand protected cassette, both containing 50 bp homology regions. Recombinants were analysed by KpnI digest. M, 1 kb+ ladder (Invitrogen). Diamond symbol indicates clones containing targeted and unmodified gap repaired plasmids. Restriction fragments sizes are (kb); p15A *P2rx1*, 6.9, 3.7, 2.6; p15 *P2rx1 genta* 6.9, 4.3, 2.6. Experiments shown in A and B were performed twice.
(TIF)

S4 Fig SPI at a different insertion site reproduces the lack of strand bias in recombination. SPI was performed at *P2rx1* site C using a terminal modified p15A *zeo* subcloning plasmid and Neomycin cassette with gbaA expression. Arrow indicates the direction of replication fork movement. Histogram values represent averages; error bars indicate standard deviation (*n* = 3). The different terminal modifications are described in Fig. 2A and S1A Fig.
(TIF)

S5 Fig Multiplex insertion but not SPI requires lagging strand recombination. (A) PCR analysis of multiplex insertion. Multiplex insertion was performed using two different antibiotic resistance cassettes both terminal modified in the same way as described in Fig. 2C. Arrow indicates the direction of replication fork movement. Recombinants were analysed using a two-step PCR screening strategy. First, the dual antibiotic resistant colonies were PCR genotyped with a homology region flanking primer and an insertion cassette specific primer. Clones positive for both insert PCRs were analysed by long range PCRs performed at each of the insertion sites using primers (dashed arrows) located outside the homology regions as shown in the schematic. The PCR products were separated by agarose gel electrophoresis and visualized with ethidium bromide staining. The insertion of both the Gentamicin and the Zeocin cassettes on the same BAC plasmid was analysed in 14 clones: Un, 6/14; Ld, 2/14; Lg, 13/14; Dual, 8/14. Key to symbols is described in Fig. 2A and S1A Fig. Shown here are representative PCR results of one such assay. The failure to detect the presence of the antibiotic cassette in some samples is possibly due to the BAC preparation or PCR conditions. Star symbol denotes clones containing BAC plasmid mixtures as determined by the presence of the wt PCR band at one site and an insert band at the other site. M, 1kb+ ladder (Invitrogen). (B) RE analysis of SPI assay. SPI recombinants were digested with EcorV and SspI and analysed by agarose gel electrophoresis. Diamond symbol indicates clones containing targeted and unmodified gap repaired plasmids. M, 1 kb ladder (NEB). Restriction fragments sizes are (kb); p15A *P2rx1*, 8.8, 4.5; p15 *P2rx1 genta* 8.8, 3.0, 2.1.
(TIF)

S6 Fig SPI efficiency does not vary across different genomic sites. (A) Insertion assay. Gentamicin cassettes were inserted at the different *P2rx1* sites using gbaA expression. Arrow indicates the direction of replication fork movement. Histogram values represent averages; error bars indicate standard deviation (*n* = 3). (B) SPI assay. SPI was performed using a p15A *zeo* subcloning plasmid and site-specific Gentamicin cassettes (*n* = 4).
(TIF)

S7 Fig Orientation of the subcloning plasmid origin has no effect on SPI. The p15A *zeo* lagging strand protected subcloning plasmid was used in SPI and contained the origin of replication in both directions. SPI was performed using a Gentamicin cassette and gbaA proteins. Arrow indicates the direction of replication fork movement. inv, Inverse orientation. Histogram values represent averages; error bars indicate standard deviation (*n* = 3).
(TIF)

S8 Fig Gap repair does not involve a single-stranded recombination intermediate. (A) Gap repair assay. (B) SPI assay. The p15A and Gentamicin ssDNA and dsDNA cassettes were recombined in gbaA expressing cells. Gap repair frequency was corrected for background abeerant recombinants (see S9 Fig.). Arrow indicates the direction of replication fork movement. Histogram values represent averages; error bars indicate standard deviation (*n* = 3).
(TIF)

S9 Fig Identification of correct gap repair recombinants. The gap repair assay shown in S8A Fig. was analysed using PCR and restriction enzyme analysis. (A) PCR analysis. Colony PCR was performed on 24 recombinants for each p15A leading and lagging strand protected ssDNA and dsDNA subcloning plasmids from different replicate experiments. A PCR assay was performed to amplify the junction of the 3’ end of the *P2rx1* subcloned insert and the p15A plasmid. The expected correct PCR amplicon size of 353 bp is indicated. The efficiency of gap repair was: p15A Ld and Lg ssDNA and p15A Ld dsDNA, 83%; p15A Lg dsDNA, 71%. M, 1kb+ ladder (Invitrogen). (B) Restriction enzyme analysis. Representative clones from panel A were analysed by KpnI digest. The numbering is consistent between the two panels. The aberrant *P2rx1* gap repair recombinants lack the full-length subcloned insert, which contains three KpnI sites and produces three fragments of sizes 6.9, 3.7 and 2.6 kb.
(TIF)

S10 Fig Greater ends-out recombination with SPI is partly site specific. Site-specific ends-out vs. ends-in SPI assays. SPI assays were performed using gbaA proteins and the ends-out or ends-in insertion cassettes described in Fig. 6F. Histogram values represent averages; error bars indicate standard deviation (*n* = 3).
(TIF)

S11 Fig Red mediated insertion and gap repair both do not require RuvABC proteins. (A) Insertion assay. (B) Gap repair. (C) SPI. Recombination assays were performed in wild type and RuvABC knockout *E*.*coli* strains using gbaA proteins and lagging strand cassettes. Arrow indicates the direction of replication fork movement. Histogram values represent averages; error bars indicate standard deviation (*n* = 2 for A and *n* = 3 for B and C). Gap repair frequency was calculated using colony PCR genotyping (*n* = 32). The RuvABC deletion strain showed lower correct gap repair frequency than wt cells (15% vs. 65%). Recombination in wild-type and *ruv* deletion strains were compared using a *t*-test: insertion, *p* = 0.3333; gap repair, 0.0005; SPI, 0.3500. However, the significant difference in gap repair frequency between wt and *ruv* strains was not reproducible using a p15A *dhfrII P2rx1* subcloning vector, which showed a modest increase in gap repair in the *ruv* strain (data not shown).
(TIF)

S12 Fig SPI cloning using multiple cassettes and large subcloning plasmids. (A) SPI was performed with gbaA proteins and different lagging strand cassettes shown in the schematic. The combination of insertion cassettes used was: 1 cassette, Neomycin; 2 cassettes, Neomycin and Gentamicin; 3 cassettes, Neomycin, Gentamicin and Hygromycin; 4 cassettes, Neomycin, Gentamicin, Hygromycin and Blasticidin. Arrow indicates the direction of replication fork movement. Histogram values represent averages; error bars, s.d. (*n* = 9). Plasmid DNA was prepared from 6 colonies for each sample and digested with SpeI and KpnI. Shown is the agarose gel electrophoresis visualized with ethidium bromide staining. Restriction fragments sizes are (kb); p15A *zeo P2rx1* gap repaired plasmid, 6.9, 2.6, 1.9, 1.8; SPI x 1, 6.9, 2.8, 2.6, 1.9; SPI x 2, 6.9, 2.8, 2.6, 2.4; SPI x 3, 6.9 3.8, 2.6, 2.4; SPI x 4, 6.9, 3.8, 3.0, 2.4. Diamond symbol indicates clones that contain targeted and unmodified gap repaired plasmids. Lanes 23 and 25 show aberrant targeting of the insertion cassettes. (B) SPI cloning using BAC subcloning plasmid. SPI was performed using a pBeloBAC11 *P2rx1* lagging strand protected plasmid (6.3 kb) and different lagging strand cassettes (*n* = 5). The combination of the insertion cassettes used was: 1 cassette, Gentamicin; 2 cassettes, Gentamicin and Neomycin. PCR genotyping was performed with a homology region flanking primer (located in the subcloning plasmid) and an insert specific primer. The 5’ and 3’ ends represent PCRs across the ends of the *P2rx1* gap repaired region. The site-specific PCRs were performed using two homology region flanking primers to amplify the full length of the inserted cassette.
(TIF)

S1 Table Numerical data from the recombination assays. (DOCX)

S2 Table DNA cassettes, subcloning plasmids and oligos used in this study. (DOCX)
