# Supplementary figures and images for: Lambda Red Mediated Gap Repair Utilizes a Novel Replicative Intermediate in Escherichia coli
Source: PLoS One. 2015 Mar 24;10(3):e0120681. doi: 10.1371/journal.pone.0120681 (PMC4372340; doi:10.1371/journal.pone.0120681)

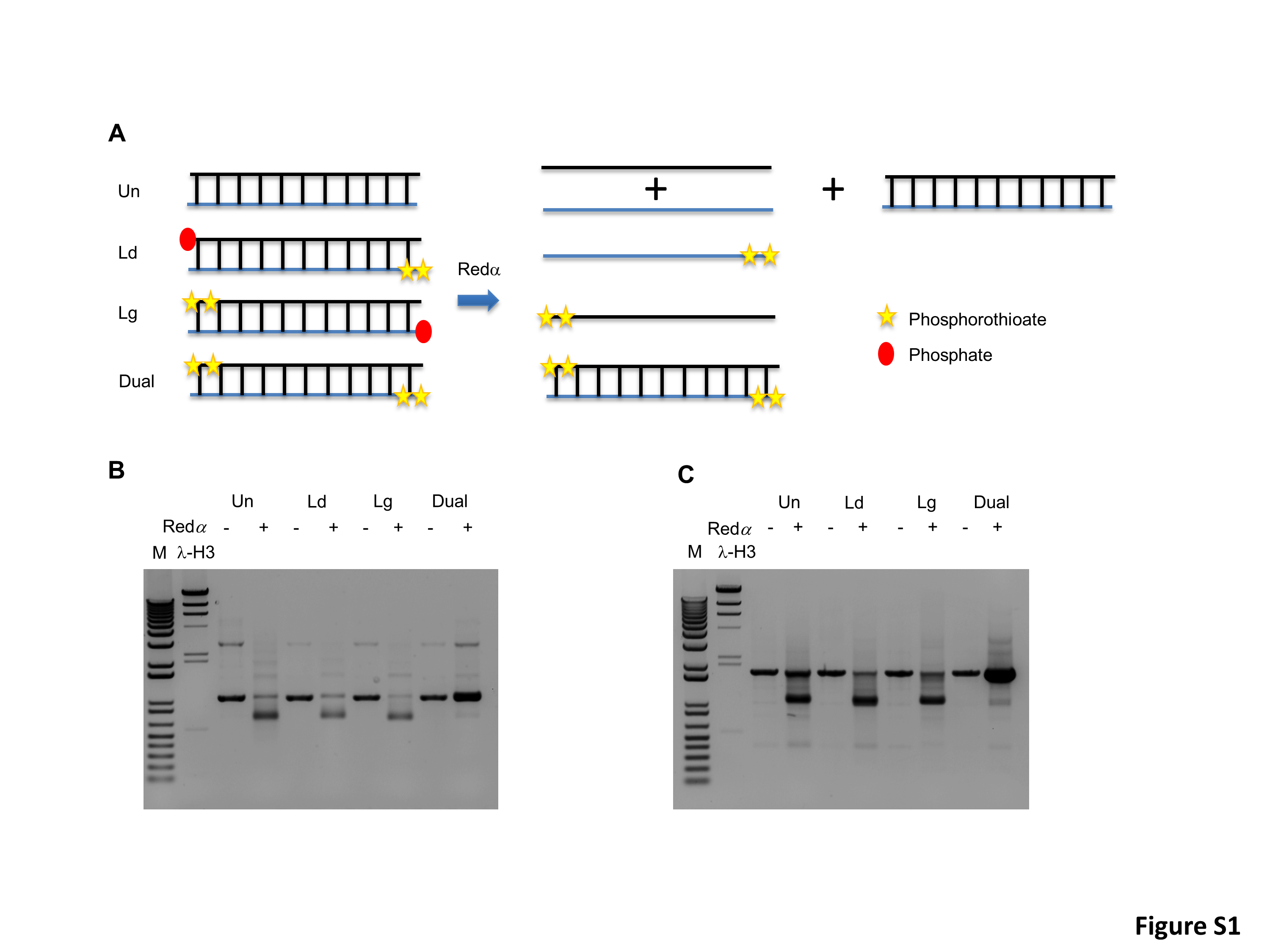

Supplement: S1 Fig — (A) Expected patterns of Redα digest of terminal modified DNA cassettes. The different terminal modifications included, Un, unmodified (hydroxylated); Ld, leading strand protected; Lg, lagging strand protected; Dual, both strands protected. (B) Redα digest of the Gentamicin insertion cassette targeting P2rx1 site D (1.0 kb). (C) Redα digest of the p15A zeo P2rx1 subcloning plasmid (1.7 kb). Each digest contained 1 μg of the dsDNA cassette and 5 U of λ–exonuclease (NEB). Control samples contained 200 ng of DNA and did not include exonuclease. The samples were analysed by agarose gel electrophoresis and ethidium bromide staining. Images were inverted and the contrast was improved (see methods). M, 1 kb+ ladder (Invitrogen); λ, Lambda HindIII digest (NEB). (TIF) [file pone.0120681.s001.TIF]

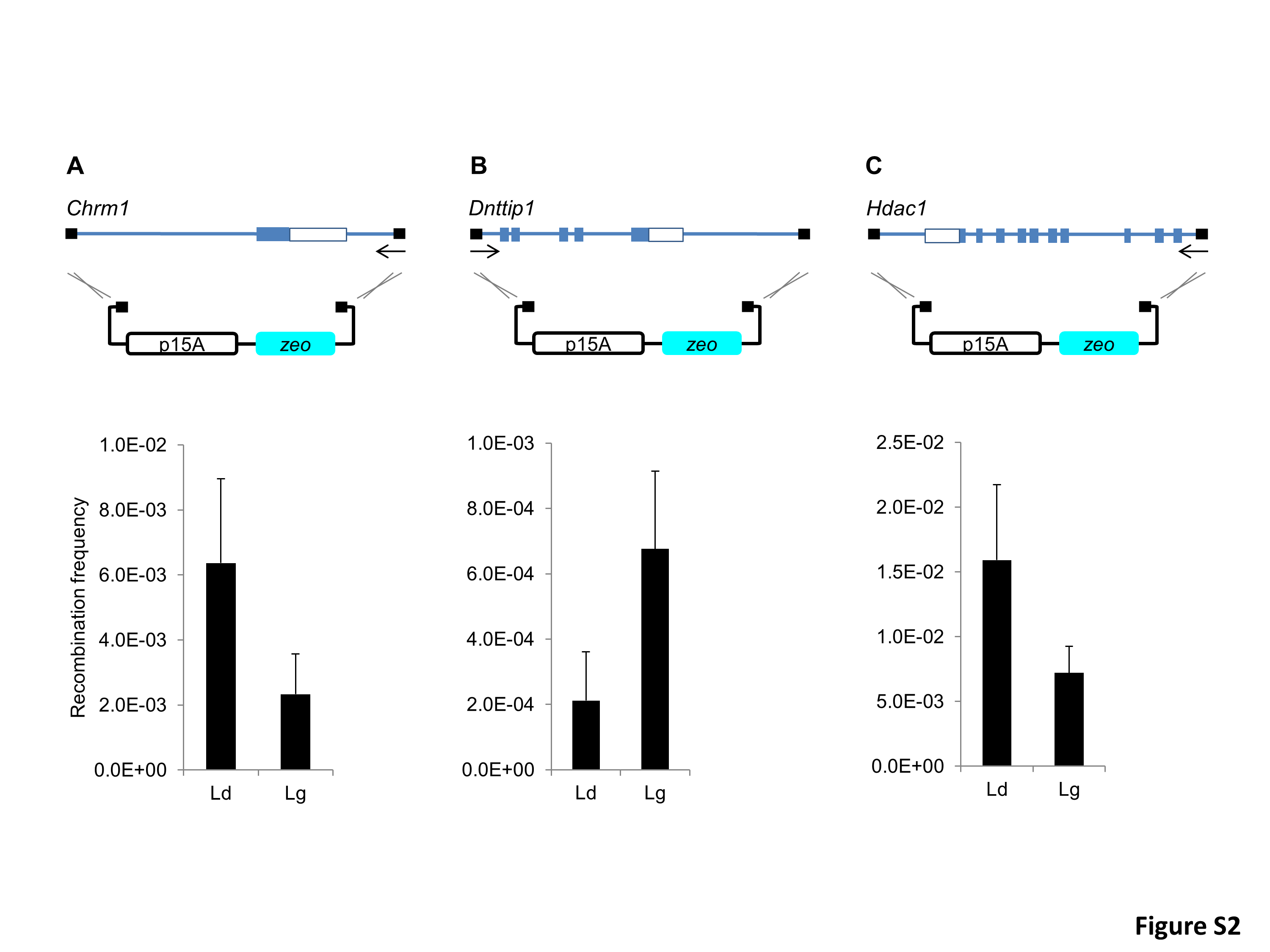

Supplement: S2 Fig — Gap repair assays were performed at three different mouse genomic loci on different BAC clones using gbaA expression and asymmetric phosphorothioated p15A zeo plasmids. (A) Chrm1. (B) Dnttip1. (C) Hdac1. Closed boxes represent exons and the open box represents the 3’UTR region. Arrow indicates the direction of replication fork movement. The Chrm1, Dnttip1 and Hdac1 gap repair insert sizes were 12 kb, 12.6 kb and 9.1 kb, respectively. Histogram values represent averages; error bars indicate standard deviation (n = 3). Ld, Leading strand protected; Lg, Lagging strand protected. Gap repair frequency at Dnttip1 was calculated using colony PCR genotyping (n = 24). The total number of recombinants at Chrm1 and Hdac1 is directly reported as the actual gap repair frequency since correct gap repair was observed in all the clones analysed for both loci (n = 24 each for Ld and Lg). A t-test did not show any significant differences between leading and lagging strand recombination: Chrm1, p = 0.1000; Dnttip1, p = 0.1000; Hdac1, p = 0.0500. (TIF) [file pone.0120681.s002.TIF]

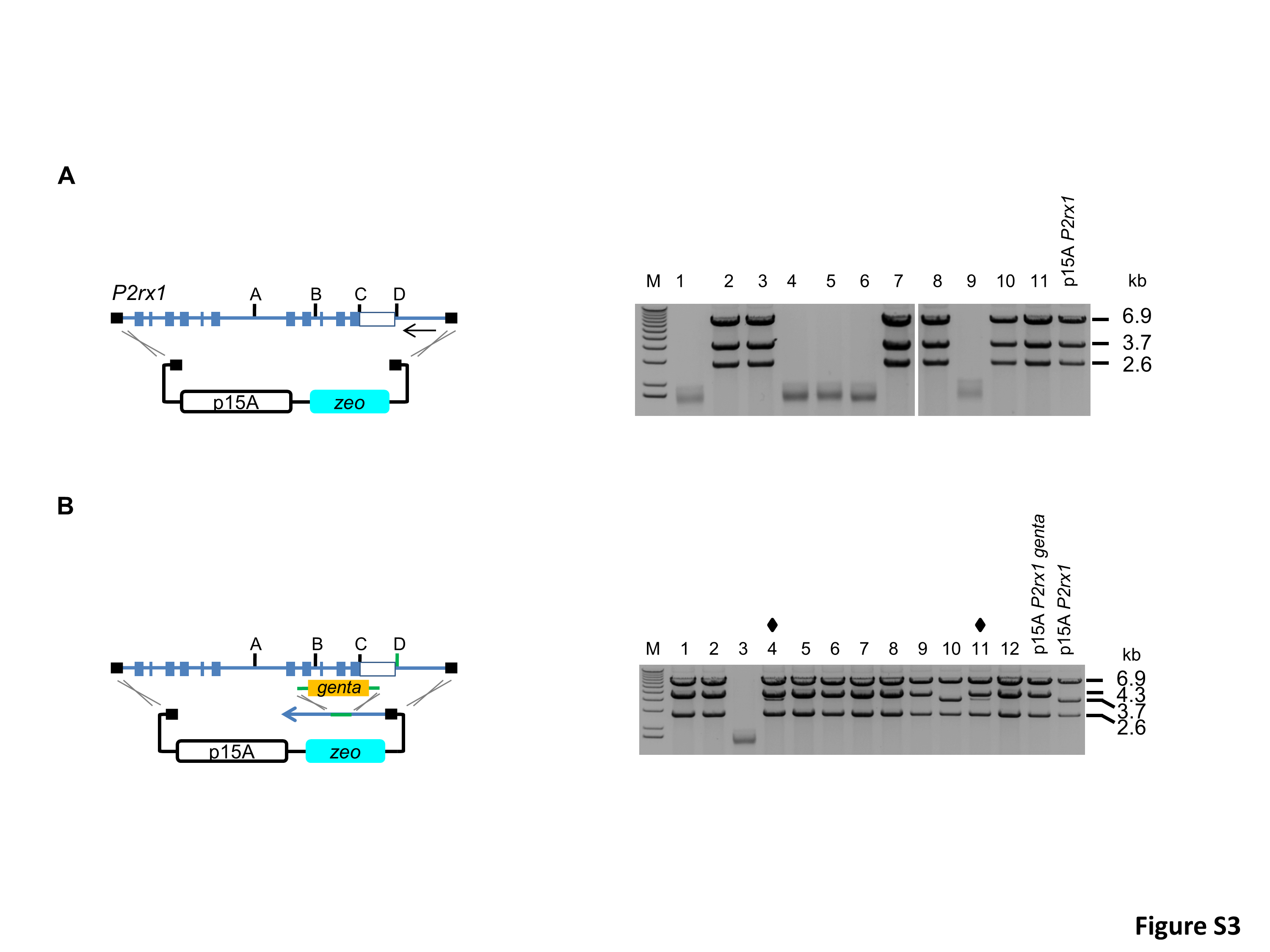

Supplement: S3 Fig — (A) Gap repair using short homologies. Gap repair was performed using gbaA proteins and a p15A lagging strand protected plasmid containing 50 bp homology regions. The recombinants were analysed by RE digests using KpnI. Arrow indicates the direction of replication fork movement. M, 1 kb+ ladder (Invitrogen). Restriction fragments sizes are (kb); p15A P2rx1, 6.9, 3.7, 2.6. (B) Subcloning plus insertion (SPI) using short homologies. SPI was performed using a p15A zeo subcloning plasmid and a Gentamicin lagging strand protected cassette, both containing 50 bp homology regions. Recombinants were analysed by KpnI digest. M, 1 kb+ ladder (Invitrogen). Diamond symbol indicates clones containing targeted and unmodified gap repaired plasmids. Restriction fragments sizes are (kb); p15A P2rx1, 6.9, 3.7, 2.6; p15 P2rx1 genta 6.9, 4.3, 2.6. Experiments shown in A and B were performed twice. (TIF) [file pone.0120681.s003.TIF]

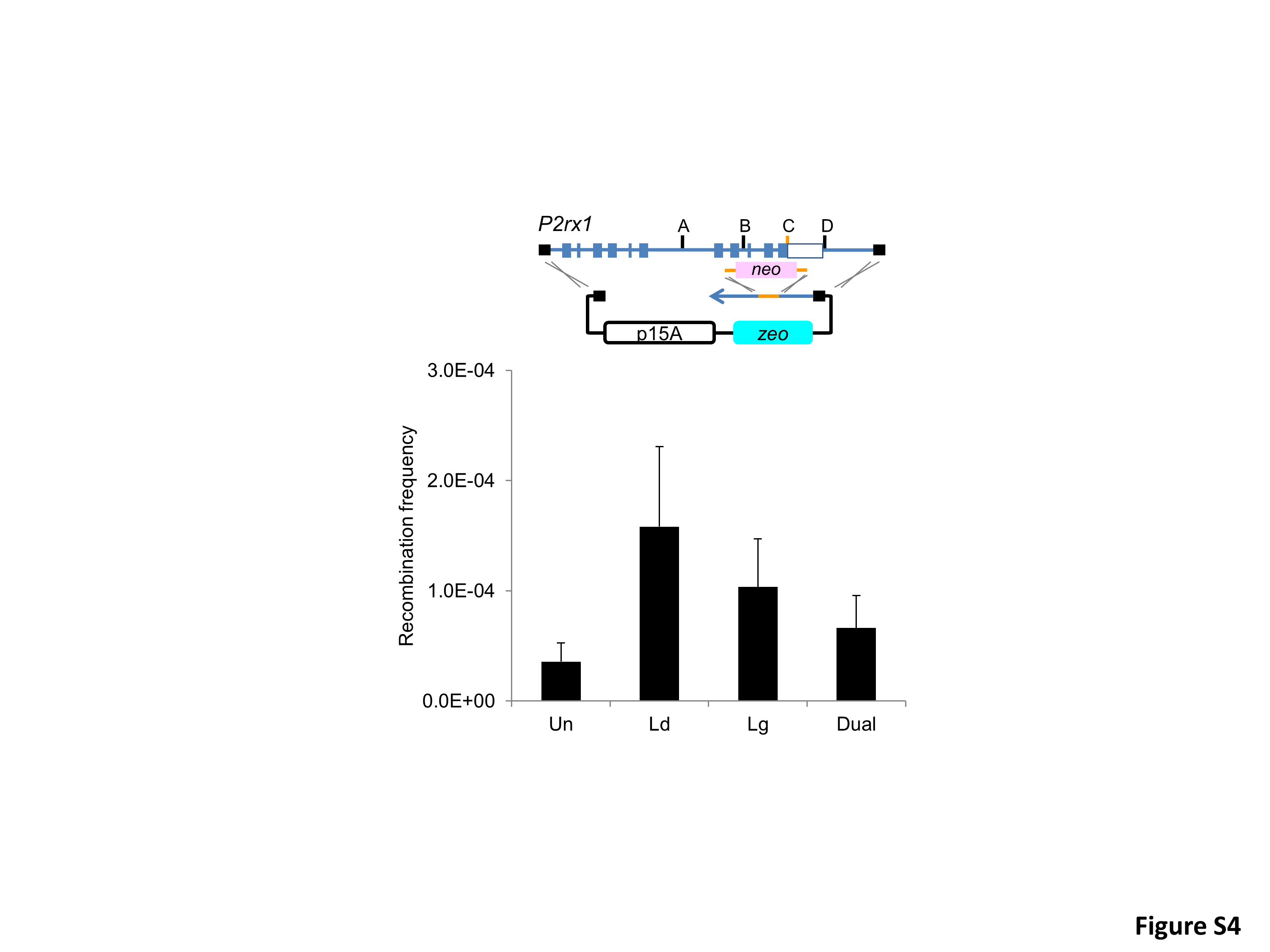

Supplement: S4 Fig — SPI was performed at P2rx1 site C using a terminal modified p15A zeo subcloning plasmid and Neomycin cassette with gbaA expression. Arrow indicates the direction of replication fork movement. Histogram values represent averages; error bars indicate standard deviation (n = 3). The different terminal modifications are described in Fig. 2A and S1A Fig. (TIF) [file pone.0120681.s004.TIF]

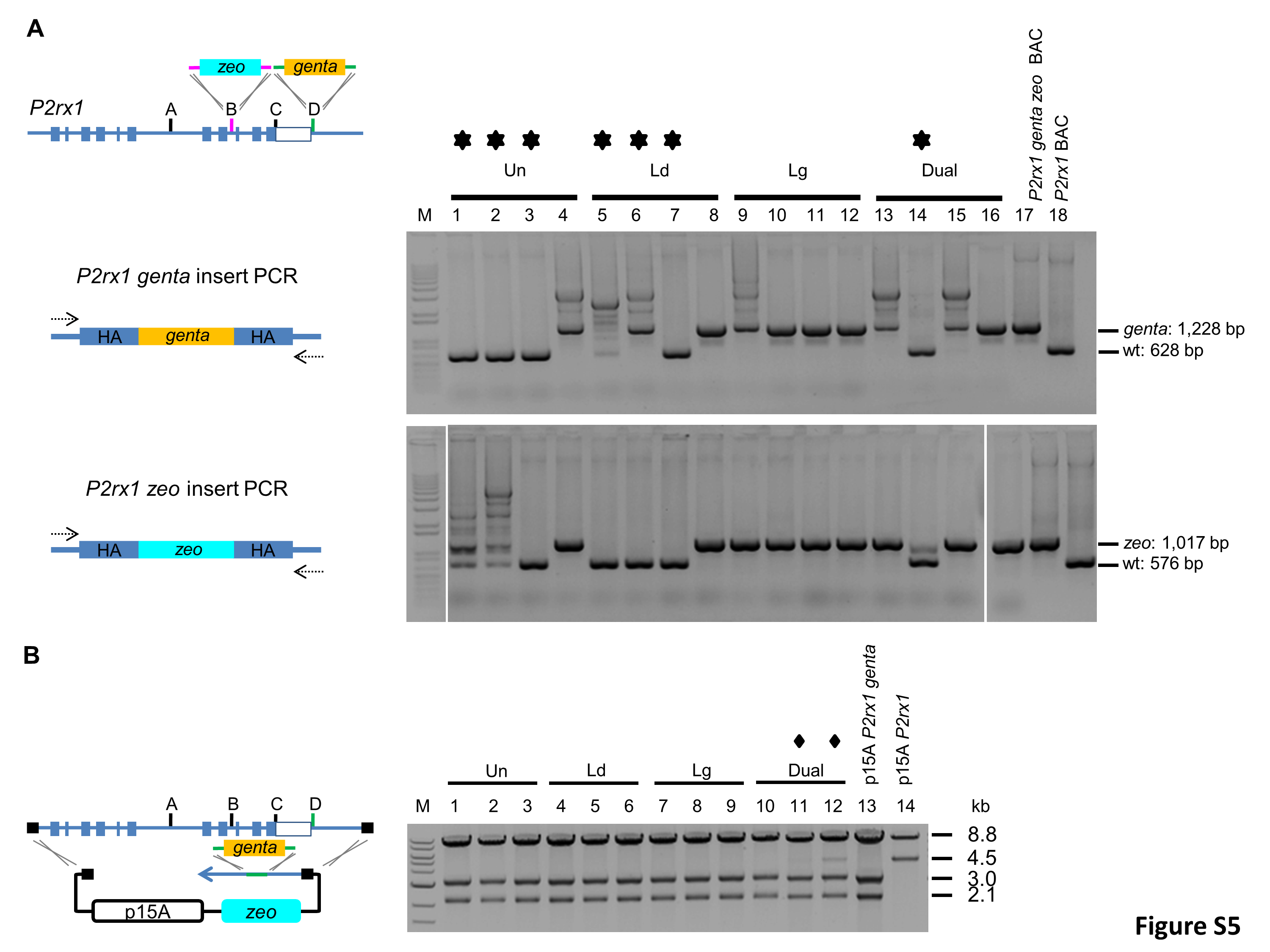

Supplement: S5 Fig — (A) PCR analysis of multiplex insertion. Multiplex insertion was performed using two different antibiotic resistance cassettes both terminal modified in the same way as described in Fig. 2C. Arrow indicates the direction of replication fork movement. Recombinants were analysed using a two-step PCR screening strategy. First, the dual antibiotic resistant colonies were PCR genotyped with a homology region flanking primer and an insertion cassette specific primer. Clones positive for both insert PCRs were analysed by long range PCRs performed at each of the insertion sites using primers (dashed arrows) located outside the homology regions as shown in the schematic. The PCR products were separated by agarose gel electrophoresis and visualized with ethidium bromide staining. The insertion of both the Gentamicin and the Zeocin cassettes on the same BAC plasmid was analysed in 14 clones: Un, 6/14; Ld, 2/14; Lg, 13/14; Dual, 8/14. Key to symbols is described in Fig. 2A and S1A Fig. Shown here are representative PCR results of one such assay. The failure to detect the presence of the antibiotic cassette in some samples is possibly due to the BAC preparation or PCR conditions. Star symbol denotes clones containing BAC plasmid mixtures as determined by the presence of the wt PCR band at one site and an insert band at the other site. M, 1kb+ ladder (Invitrogen). (B) RE analysis of SPI assay. SPI recombinants were digested with EcorV and SspI and analysed by agarose gel electrophoresis. Diamond symbol indicates clones containing targeted and unmodified gap repaired plasmids. M, 1 kb ladder (NEB). Restriction fragments sizes are (kb); p15A P2rx1, 8.8, 4.5; p15 P2rx1 genta 8.8, 3.0, 2.1. (TIF) [file pone.0120681.s005.TIF]

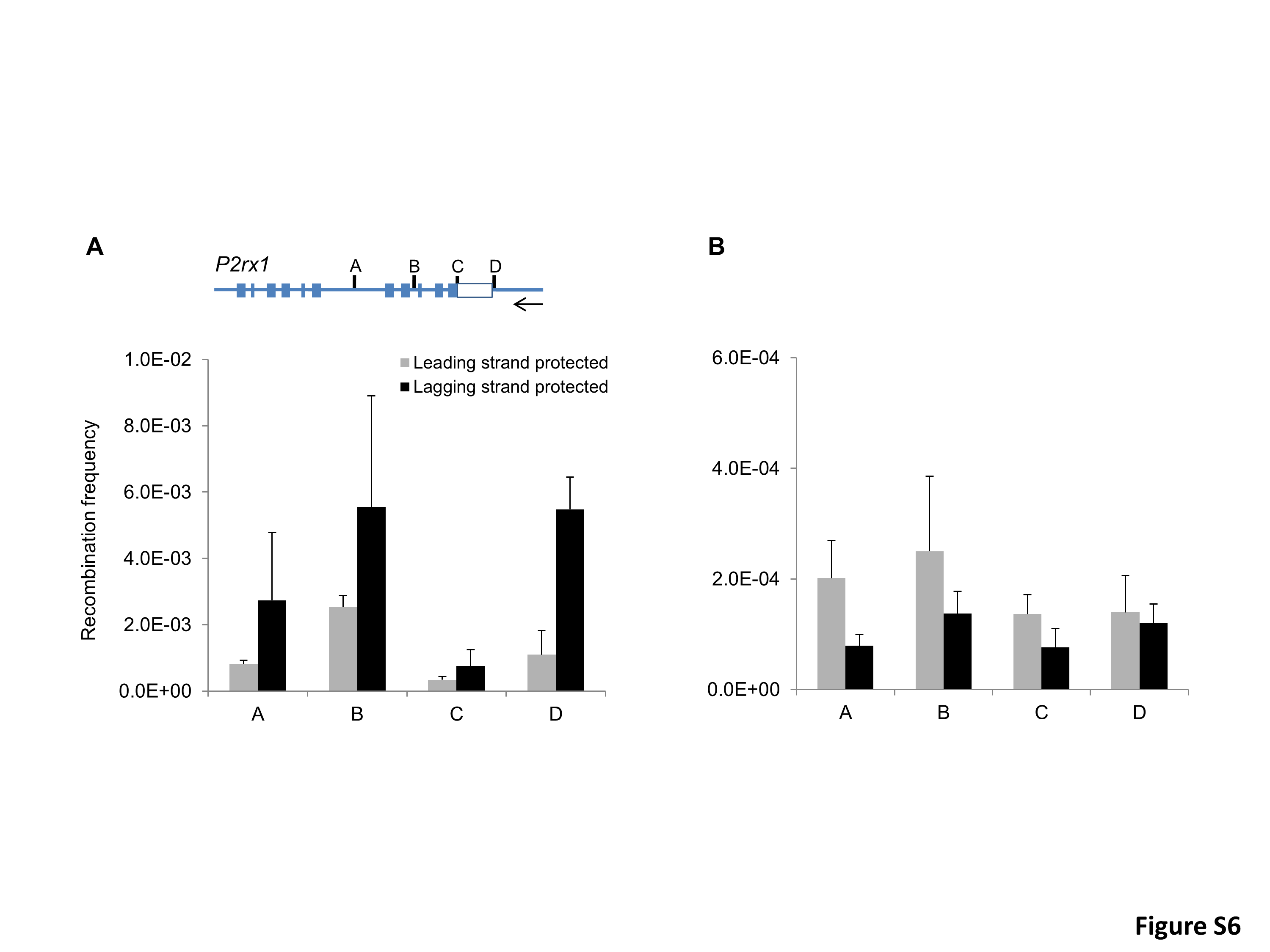

Supplement: S6 Fig — (A) Insertion assay. Gentamicin cassettes were inserted at the different P2rx1 sites using gbaA expression. Arrow indicates the direction of replication fork movement. Histogram values represent averages; error bars indicate standard deviation (n = 3). (B) SPI assay. SPI was performed using a p15A zeo subcloning plasmid and site-specific Gentamicin cassettes (n = 4). (TIF) [file pone.0120681.s006.TIF]

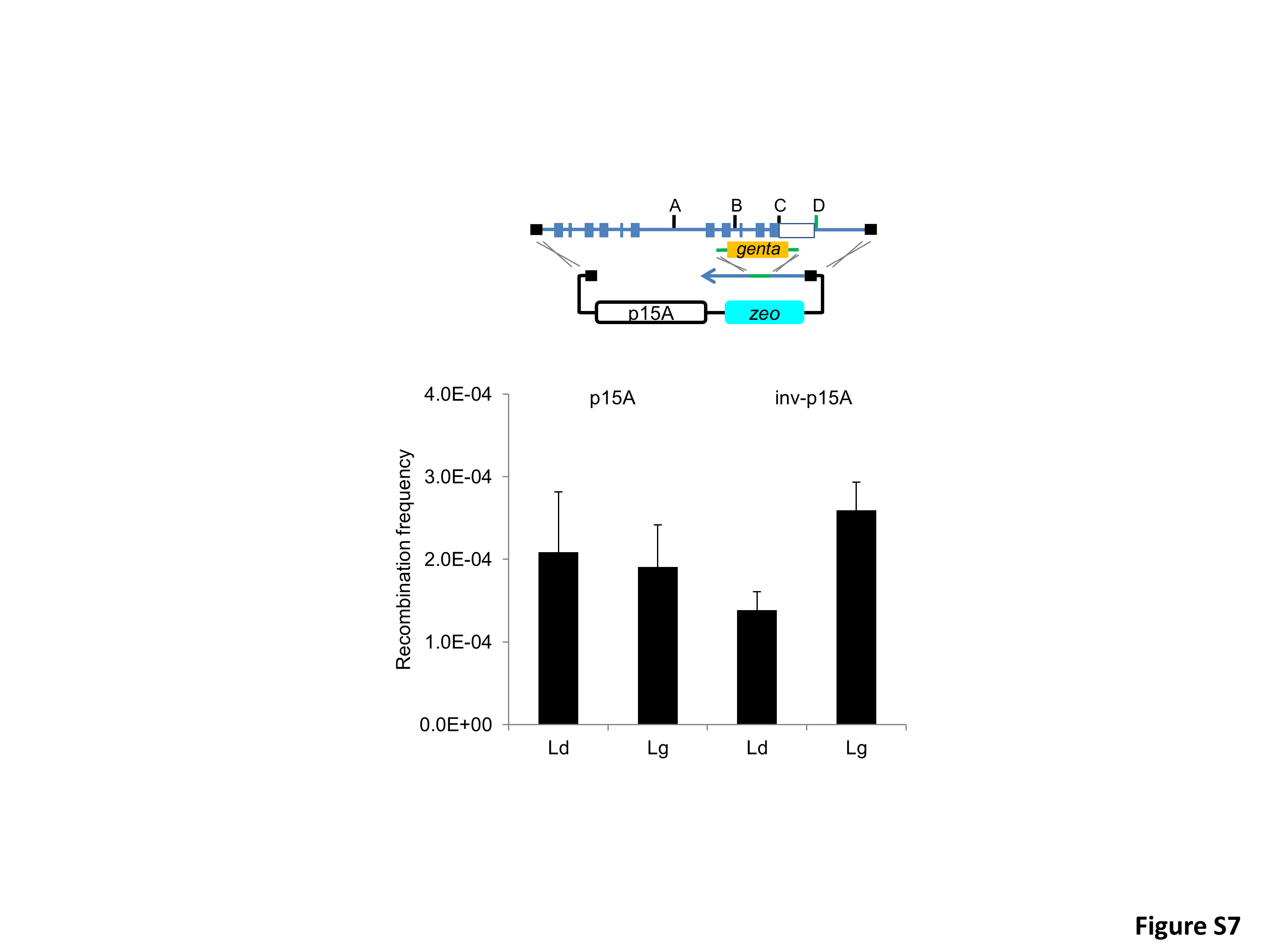

Supplement: S7 Fig — The p15A zeo lagging strand protected subcloning plasmid was used in SPI and contained the origin of replication in both directions. SPI was performed using a Gentamicin cassette and gbaA proteins. Arrow indicates the direction of replication fork movement. inv, Inverse orientation. Histogram values represent averages; error bars indicate standard deviation (n = 3). (TIF) [file pone.0120681.s007.TIF]

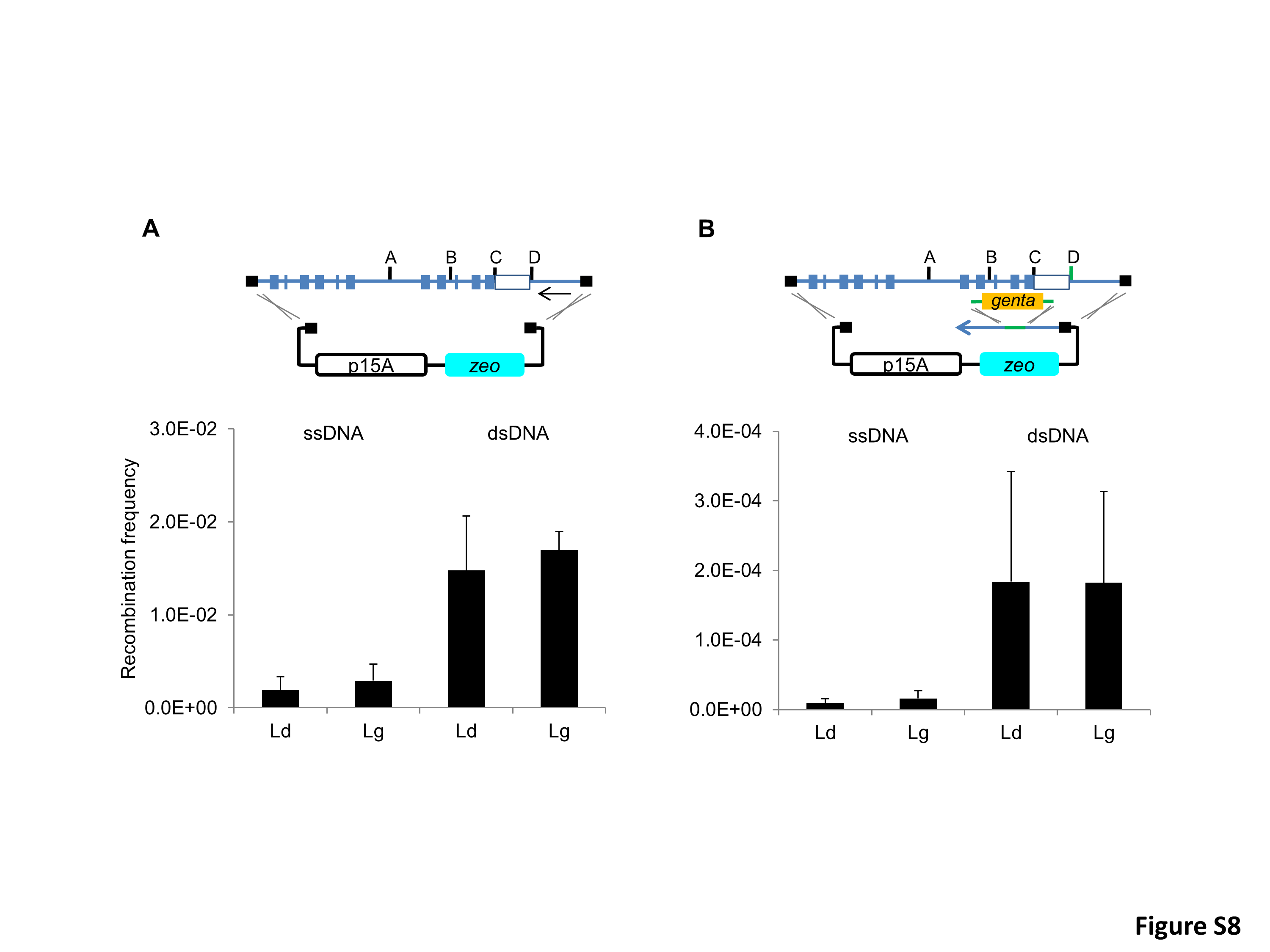

Supplement: S8 Fig — (A) Gap repair assay. (B) SPI assay. The p15A and Gentamicin ssDNA and dsDNA cassettes were recombined in gbaA expressing cells. Gap repair frequency was corrected for background abeerant recombinants (see S9 Fig.). Arrow indicates the direction of replication fork movement. Histogram values represent averages; error bars indicate standard deviation (n = 3). (TIF) [file pone.0120681.s008.TIF]

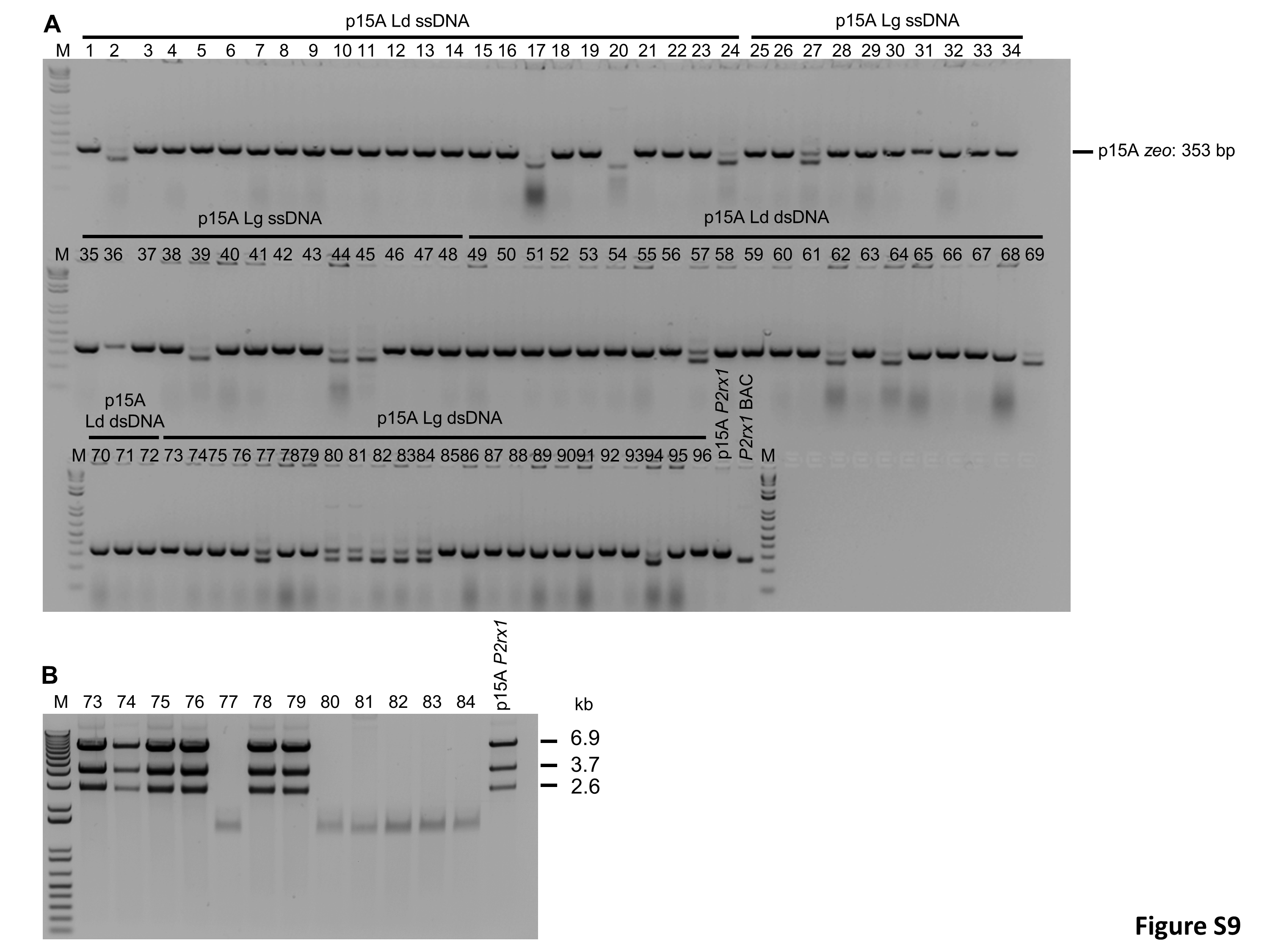

Supplement: S9 Fig — The gap repair assay shown in S8A Fig. was analysed using PCR and restriction enzyme analysis. (A) PCR analysis. Colony PCR was performed on 24 recombinants for each p15A leading and lagging strand protected ssDNA and dsDNA subcloning plasmids from different replicate experiments. A PCR assay was performed to amplify the junction of the 3’ end of the P2rx1 subcloned insert and the p15A plasmid. The expected correct PCR amplicon size of 353 bp is indicated. The efficiency of gap repair was: p15A Ld and Lg ssDNA and p15A Ld dsDNA, 83%; p15A Lg dsDNA, 71%. M, 1kb+ ladder (Invitrogen). (B) Restriction enzyme analysis. Representative clones from panel A were analysed by KpnI digest. The numbering is consistent between the two panels. The aberrant P2rx1 gap repair recombinants lack the full-length subcloned insert, which contains three KpnI sites and produces three fragments of sizes 6.9, 3.7 and 2.6 kb. (TIF) [file pone.0120681.s009.TIF]

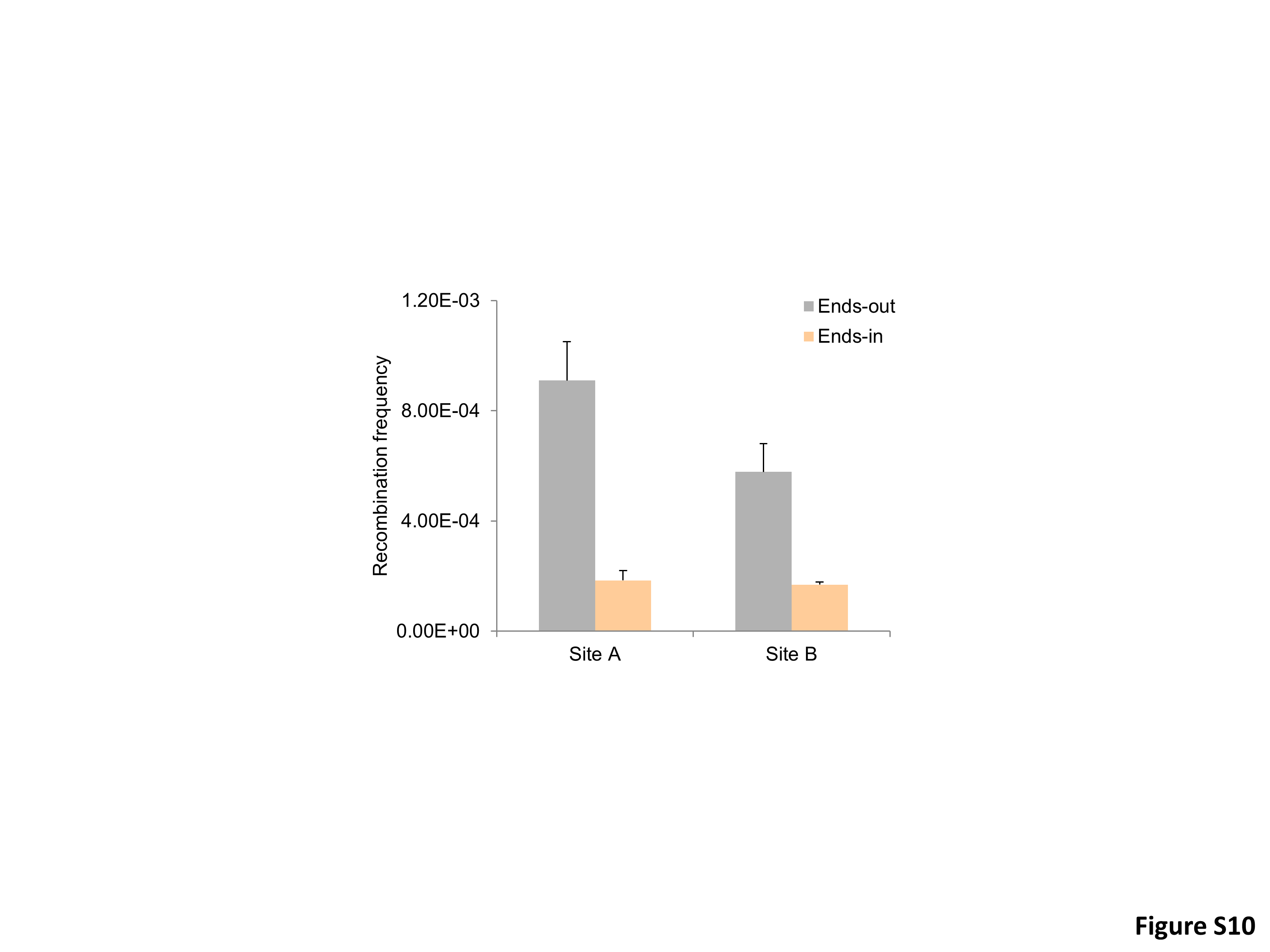

Supplement: S10 Fig — Site-specific ends-out vs. ends-in SPI assays. SPI assays were performed using gbaA proteins and the ends-out or ends-in insertion cassettes described in Fig. 6F. Histogram values represent averages; error bars indicate standard deviation (n = 3). (TIF) [file pone.0120681.s010.TIF]

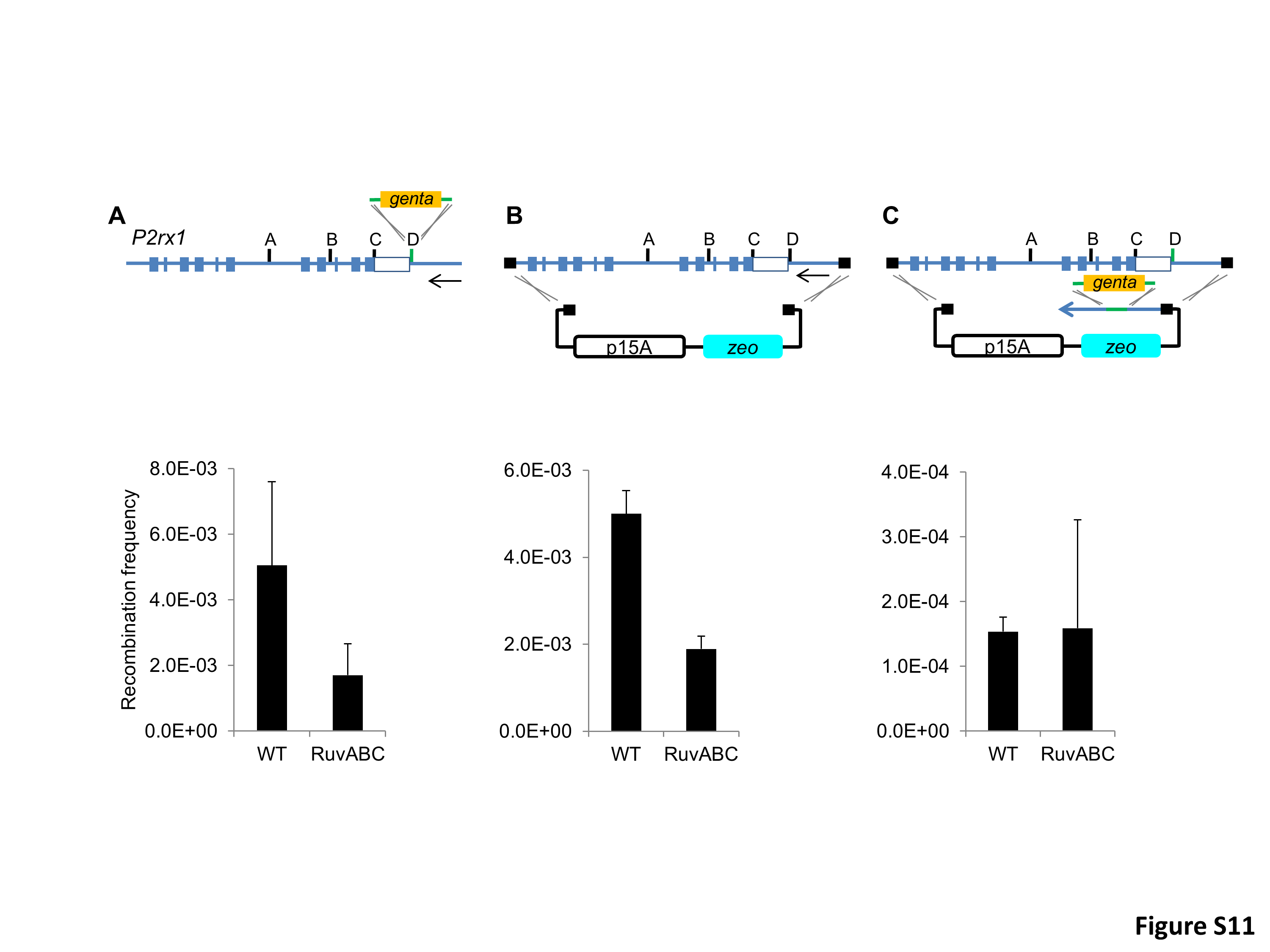

Supplement: S11 Fig — (A) Insertion assay. (B) Gap repair. (C) SPI. Recombination assays were performed in wild type and RuvABC knockout E.coli strains using gbaA proteins and lagging strand cassettes. Arrow indicates the direction of replication fork movement. Histogram values represent averages; error bars indicate standard deviation (n = 2 for A and n = 3 for B and C). Gap repair frequency was calculated using colony PCR genotyping (n = 32). The RuvABC deletion strain showed lower correct gap repair frequency than wt cells (15% vs. 65%). Recombination in wild-type and ruv deletion strains were compared using a t-test: insertion, p = 0.3333; gap repair, 0.0005; SPI, 0.3500. However, the significant difference in gap repair frequency between wt and ruv strains was not reproducible using a p15A dhfrII P2rx1 subcloning vector, which showed a modest increase in gap repair in the ruv strain (data not shown). (TIF) [file pone.0120681.s011.TIF]

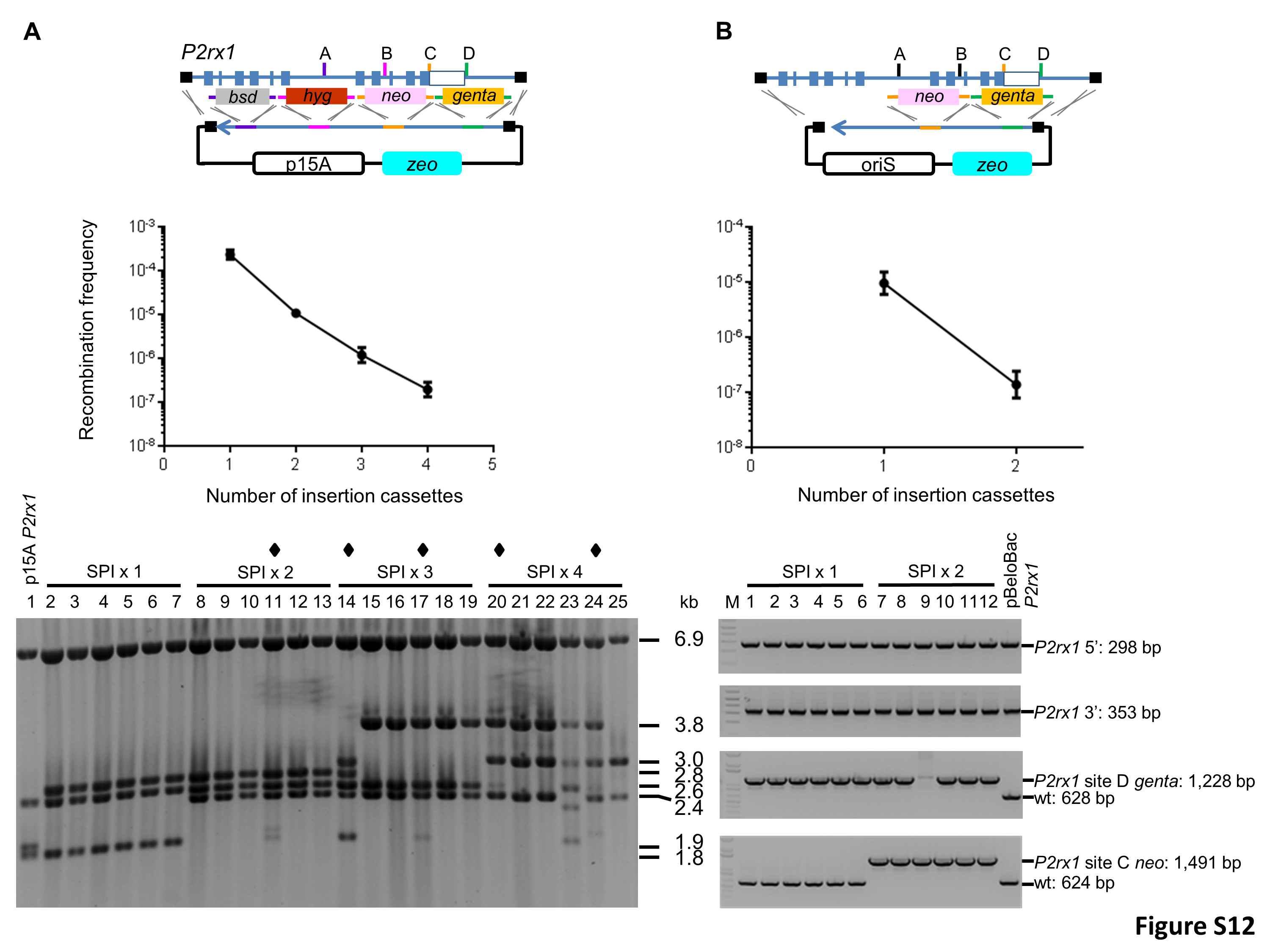

Supplement: S12 Fig — (A) SPI was performed with gbaA proteins and different lagging strand cassettes shown in the schematic. The combination of insertion cassettes used was: 1 cassette, Neomycin; 2 cassettes, Neomycin and Gentamicin; 3 cassettes, Neomycin, Gentamicin and Hygromycin; 4 cassettes, Neomycin, Gentamicin, Hygromycin and Blasticidin. Arrow indicates the direction of replication fork movement. Histogram values represent averages; error bars, s.d. (n = 9). Plasmid DNA was prepared from 6 colonies for each sample and digested with SpeI and KpnI. Shown is the agarose gel electrophoresis visualized with ethidium bromide staining. Restriction fragments sizes are (kb); p15A zeo P2rx1 gap repaired plasmid, 6.9, 2.6, 1.9, 1.8; SPI x 1, 6.9, 2.8, 2.6, 1.9; SPI x 2, 6.9, 2.8, 2.6, 2.4; SPI x 3, 6.9 3.8, 2.6, 2.4; SPI x 4, 6.9, 3.8, 3.0, 2.4. Diamond symbol indicates clones that contain targeted and unmodified gap repaired plasmids. Lanes 23 and 25 show aberrant targeting of the insertion cassettes. (B) SPI cloning using BAC subcloning plasmid. SPI was performed using a pBeloBAC11 P2rx1 lagging strand protected plasmid (6.3 kb) and different lagging strand cassettes (n = 5). The combination of the insertion cassettes used was: 1 cassette, Gentamicin; 2 cassettes, Gentamicin and Neomycin. PCR genotyping was performed with a homology region flanking primer (located in the subcloning plasmid) and an insert specific primer. The 5’ and 3’ ends represent PCRs across the ends of the P2rx1 gap repaired region. The site-specific PCRs were performed using two homology region flanking primers to amplify the full length of the inserted cassette. (TIF) [file pone.0120681.s012.tif]
