# Supplementary material for: Lambda Red Mediated Gap Repair Utilizes a Novel Replicative Intermediate in Escherichia coli
Source: PLoS One. 2015 Mar 24;10(3):e0120681. doi: 10.1371/journal.pone.0120681 (PMC4372340; doi:10.1371/journal.pone.0120681)
Supplement: S1 Table — (DOCX) [file pone.0120681.s013.docx]

**Table S1 Numerical data from the recombination assays**

| **Fig. 2A** |  |  |  |  |  |  |  |  |
| --- | --- | --- | --- | --- | --- | --- | --- | --- |
|  | Un | Ld | Lg | Dual |  |  |  |  |
| Average^a^ | 4.1E-04 | 2.4E-04 | 4.8E-03 | 2.6E-03 |  |  |  |  |
| SD^b^ | 1.4E-04 | 1.7E-04 | 2.6E-03 | 1.2E-03 |  |  |  |  |
|  |  |  |  |  |  |  |  |  |
| **Fig. 2B** |  |  |  |  |  |  |  |  |
| Average | 2.0E-03 | 3.7E-03 | 4.1E-03 | 3.9E-03 |  |  |  |  |
| SD | 9.9E-04 | 8.2E-04 | 1.2E-03 | 7.8E-04 |  |  |  |  |
|  |  |  |  |  |  |  |  |  |
| **Fig. 2C** |  |  |  |  |  |  |  |  |
| Average | 1.5E-05 | 6.1E-05 | 4.0E-04 | 1.5E-04 |  |  |  |  |
| SD | 7.3E-06 | 2.9E-05 | 5.6E-05 | 5.3E-05 |  |  |  |  |
|  |  |  |  |  |  |  |  |  |
| **Fig. 2D** |  |  |  |  |  |  |  |  |
| Average | 3.7E-05 | 1.5E-04 | 1.5E-04 | 1.0E-04 |  |  |  |  |
| SD | 5.8E-06 | 2.3E-05 | 2.4E-04 | 7.1E-05 |  |  |  |  |
|  |  |  |  |  |  |  |  |  |
| **Fig. 3A** |  |  |  |  |  |  |  |  |
|  | Ld | Lg |  |  |  |  |  |  |
| Average | 4.6E-04 | 3.0E-03 |  |  |  |  |  |  |
| SD | 3.0E-04 | 2.2E-03 |  |  |  |  |  |  |
|  |  |  |  |  |  |  |  |  |
| **Fig. 3B** |  |  |  |  |  |  |  |  |
|  | Ld | Lg | Ld+Lg | Lg+Ld |  |  |  |  |
| Average | 8.5E-04 | 1.1E-03 | 1.6E-03 | 1.3E-03 |  |  |  |  |
| SD | 1.4E-04 | 3.5E-04 | 5.0E-04 | 6.3E-04 |  |  |  |  |
|  |  |  |  |  |  |  |  |  |
| **Fig. 4A** |  |  |  |  |  |  |  |  |
|  | Amp/Chl | Genta/Chl | Zeo/Genta | Zeo/Amp |  |  |  |  |
| Average | 4.0E+04 | 8.7E+04 | 3.8E+05 | 9.4E+04 |  |  |  |  |
| SD | 2.6E+04 | 3.7E+04 | 1.6E+05 | 4.3E+04 |  |  |  |  |
|  |  |  |  |  |  |  |  |  |
| **Fig. 4B** |  |  |  |  |  |  |  |  |
| Average | 5.9E+04 | 5.5E+03 | 6.5E+04 | 6.6E+05 |  |  |  |  |
| SD | 8.2E+03 | 1.6E+03 | 2.7E+04 | 6.0E+04 |  |  |  |  |
|  |  |  |  |  |  |  |  |  |
| **Fig. 4C** |  |  |  |  |  |  |  |  |
| Average | 5.0E+04 | 4.3E+04 | 3.7E+05 | 7.8E+04 |  |  |  |  |
| SD | 2.9E+04 | 3.2E+04 | 1.3E+05 | 3.2E+04 |  |  |  |  |
|  |  |  |  |  |  |  |  |  |
| **Fig. 5A** |  |  |  |  |  |  |  |  |
|  | Ld | Lg | Ld | Lg |  |  |  |  |
| Average | 8.6E-06 | 4.0E-05 | 9.0E-06 | 2.4E-04 |  |  |  |  |
| SD | 1.9E-06 | 1.4E-05 | 4.3E-06 | 6.3E-05 |  |  |  |  |
|  |  |  |  |  |  |  |  |  |
| **Fig. 5B** |  |  |  |  |  |  |  |  |
| Average | 5.4E-05 | 1.2E-03 | 1.2E-04 | 2.8E-04 |  |  |  |  |
| SD | 1.0E-05 | 2.3E-04 | 5.2E-05 | 5.1E-05 |  |  |  |  |
|  |  |  |  |  |  |  |  |  |
| **Fig. 5D** |  |  |  |  |  |  |  |  |
|  | Ld | Lg | Ld | Lg |  |  |  |  |
|  | ssDNA | ssDNA | dsDNA | dsDNA |  |  |  |  |
| Average | 1.4E-03 | 1.8E-02 | 1.4E-03 | 6.9E-03 |  |  |  |  |
| SD | 4.4E-04 | 1.3E-02 | 4.0E-04 | 1.2E-03 |  |  |  |  |
|  |  |  |  |  |  |  |  |  |
| **Fig. 5F** |  |  |  |  |  |  |  |  |
| Average | 2.1E-04 | 2.4E-04 | 1.9E-04 | 4.9E-04 |  |  |  |  |
| SD | 5.8E-05 | 4.6E-05 | 2.3E-05 | 2.2E-04 |  |  |  |  |
|  |  |  |  |  |  |  |  |  |
| **Fig. 6C** |  |  |  |  |  |  |  |  |
|  | Ld | Lg | Ld | Lg |  |  |  |  |
|  | Ends-in | Ends-in | Ends-out | Ends-out |  |  |  |  |
| Average | 1.3E-04 | 5.1E-05 | 1.2E-04 | 2.6E-03 |  |  |  |  |
| SD | 5.5E-05 | 3.0E-05 | 7.9E-05 | 1.5E-03 |  |  |  |  |
|  |  |  |  |  |  |  |  |  |
| **Fig. 6D** |  |  |  |  |  |  |  |  |
| Average | 4.0E-05 | 4.0E-05 | 6.0E-05 | 5.7E-05 |  |  |  |  |
| SD | 5.0E-05 | 1.5E-05 | 2.9E-05 | 7.8E-04 |  |  |  |  |
|  |  |  |  |  |  |  |  |  |
| **Fig. 6E** |  |  |  |  |  |  |  |  |
|  | Insertion cassettes | |  |  |  |  |  |  |
|  | Ends-out | Ends-out | Ends-out | Ends-in | Ends-in | Ends-in |  |  |
|  | 1 | 2 | 3 | 1 | 2 | 3 |  |  |
| Average | 2.2E-03 | 1.4E-04 | 1.4E-05 | 2.4E-05 | 2.1E-06 | 7.7E-08 |  |  |
| SD | 5.0E-04 | 6.4E-05 | 7.4E-06 | 1.3E-05 | 3.1E-08 | 4.2E-08 |  |  |
|  |  |  |  |  |  |  |  |  |
| **Fig. 6F** |  |  |  |  |  |  |  |  |
| Average | 2.4E-04 | 1.8E-05 | 2.3E-06 | 1.4E-04 | 3.2E-06 | 1.2E-07 |  |  |
| SD | 1.0E-04 | 7.9E-06 | 8.0E-07 | 3.5E-05 | 2.0E-06 | 4.7E-08 |  |  |
|  |  |  |  |  |  |  |  |  |
| **Fig. S2A** |  |  |  |  |  |  |  |  |
|  | Ld | Lg |  |  |  |  |  |  |
| Average | 6.4E-03 | 2.3E-03 |  |  |  |  |  |  |
| SD | 2.6E-03 | 1.2E-03 |  |  |  |  |  |  |
|  |  |  |  |  |  |  |  |  |
| **Fig. S2B** |  |  |  |  |  |  |  |  |
| Average | 2.1E-04 | 6.8E-04 |  |  |  |  |  |  |
| SD | 1.5E-04 | 2.4E-04 |  |  |  |  |  |  |
|  |  |  |  |  |  |  |  |  |
| **Fig. S2C** |  |  |  |  |  |  |  |  |
| Average | 1.6E-02 | 7.2E-03 |  |  |  |  |  |  |
| SD | 5.8E-03 | 2.0E-03 |  |  |  |  |  |  |
|  |  |  |  |  |  |  |  |  |
| **Fig. S4** |  |  |  |  |  |  |  |  |
|  | Un | Ld | Lg | Dual |  |  |  |  |
| Average | 3.6E-05 | 1.6E-04 | 1.0E-04 | 6.6E-05 |  |  |  |  |
| SD | 1.7E-05 | 7.3E-05 | 4.4E-05 | 2.9E-05 |  |  |  |  |
|  |  |  |  |  |  |  |  |  |
| **Fig. S6A** |  |  |  |  |  |  |  |  |
|  | A | A | B | B | C | C | D | D |
|  | Ld | Lg | Ld | Lg | Ld | Lg | Ld | Lg |
| Average | 8.1E-04 | 2.7E-03 | 2.5E-03 | 5.6E-03 | 3.4E-04 | 7.5E-04 | 1.1E-03 | 5.5E-03 |
| SD | 1.2E-04 | 2.0E-03 | 3.5E-04 | 3.3E-03 | 1.1E-04 | 4.9E-04 | 7.2E-04 | 9.8E-04 |
|  |  |  |  |  |  |  |  |  |
| **Fig. S6B** |  |  |  |  |  |  |  |  |
| Average | 2.0E-04 | 7.9E-05 | 2.5E-04 | 1.4E-04 | 1.4E-04 | 7.6E-05 | 1.4E-04 | 1.2E-04 |
| SD | 6.8E-05 | 2.0E-05 | 1.4E-04 | 4.0E-05 | 3.5E-05 | 3.5E-05 | 6.6E-05 | 3.5E-05 |
|  |  |  |  |  |  |  |  |  |
| **Fig. S7** |  |  |  |  |  |  |  |  |
|  | p15A | p15A | p15A Inv | p15A Inv |  |  |  |  |
|  | Ld | Lg | Ld | Lg |  |  |  |  |
| Average | 2.1E-04 | 1.9E-04 | 1.4E-04 | 2.6E-04 |  |  |  |  |
| SD | 7.3E-05 | 5.1E-05 | 2.3E-05 | 3.4E-05 |  |  |  |  |
|  |  |  |  |  |  |  |  |  |
| **Fig. S8A** |  |  |  |  |  |  |  |  |
|  | ssDNA | ssDNA | dsDNA | dsDNA |  |  |  |  |
|  | Ld | Lg | Ld | Lg |  |  |  |  |
| Average | 1.9E-03 | 2.9E-03 | 1.5E-02 | 1.7E-02 |  |  |  |  |
| SD | 1.4E-03 | 1.8E-03 | 5.9E-03 | 2.0E-03 |  |  |  |  |
|  |  |  |  |  |  |  |  |  |
| **Fig. S8B** |  |  |  |  |  |  |  |  |
|  | ssDNA | ssDNA | dsDNA | dsDNA |  |  |  |  |
|  | Ld | Lg | Ld | Lg |  |  |  |  |
| Average | 9.4E-06 | 1.6E-05 | 1.8E-04 | 1.8E-04 |  |  |  |  |
| SD | 6.0E-06 | 1.1E-05 | 1.6E-04 | 1.3E-04 |  |  |  |  |
|  |  |  |  |  |  |  |  |  |
| **Fig. S10** |  |  |  |  |  |  |  |  |
|  | A | A | B | B |  |  |  |  |
|  | Ends-out | Ends-in | Ends-out | Ends-in |  |  |  |  |
| Average | 9.1E-04 | 1.8E-04 | 5.8E-04 | 1.7E-04 |  |  |  |  |
| SD | 1.4E-04 | 3.7E-05 | 1.0E-04 | 9.5E-06 |  |  |  |  |
|  |  |  |  |  |  |  |  |  |
| **Fig. S11A** |  |  |  |  |  |  |  |  |
|  | WT | RuvABC |  |  |  |  |  |  |
| Average | 5.1E-03 | 1.7E-03 |  |  |  |  |  |  |
| SD | 3.6E-03 | 1.4E-03 |  |  |  |  |  |  |
|  |  |  |  |  |  |  |  |  |
| **Fig. S11B** |  |  |  |  |  |  |  |  |
| Average | 5.0E-03 | 1.9E-03 |  |  |  |  |  |  |
| **SD** | **5.3E-04** | **3.0E-04** |  |  |  |  |  |  |
|  |  |  |  |  |  |  |  |  |
| **Fig. S11C** |  |  |  |  |  |  |  |  |
| Average | 1.5E-04 | 1.6E-04 |  |  |  |  |  |  |
| SD | 2.2E-05 | 1.7E-04 |  |  |  |  |  |  |
|  |  |  |  |  |  |  |  |  |
| **Fig. S12A** |  |  |  |  |  |  |  |  |
|  | Insertion cassettes | |  |  |  |  |  |  |
|  | 1 | 2 | 2 | 4 |  |  |  |  |
| Average | 2.3E-04 | 1.7E-05 | 1.1E-06 | 1.9E-07 |  |  |  |  |
| SD | 1.6E-04 | 7.6E-06 | 1.5E-06 | 2.7E-07 |  |  |  |  |
|  |  |  |  |  |  |  |  |  |
| **Fig. S12B** |  |  |  |  |  |  |  |  |
| Average | 9.5E-06 | 1.4E-07 |  |  |  |  |  |  |
| SD | 1.3E-05 | 2.3E-07 |  |  |  |  |  |  |

^a^Recombination frequency was determined by dividing the total number of antibiotic resistant colonies by the total number of colonies surviving after electroporation. Average recombination frequency was determined from multiple independent experiments (see figure legends for the number of replicates). Gap repair values were corrected for aberrant recombinants.

^b^Standard deviation
