# Supplementary material for: Lambda Red Mediated Gap Repair Utilizes a Novel Replicative Intermediate in Escherichia coli
Source: PLoS One. 2015 Mar 24;10(3):e0120681. doi: 10.1371/journal.pone.0120681 (PMC4372340; doi:10.1371/journal.pone.0120681)
Supplement: S2 Table — (DOCX) [file pone.0120681.s014.docx]

**Table S2 DNA cassettes, subcloning plasmids and oligos used in this study**

|  |
| --- |

Insertion cassettes

Homology regions

EM7 promoter

Antibiotic resistance marker

*P2rx1* site A Blasticidin cassette:

CACCTGCTAGTTAGCATTGCAGCTCTGCCCTGCTGGGACAATCCTATACCCTGTTCTTGCTGGATCCTTCTTGTGCCTGCTTCTGTAGCTGCCTATGTGGGAATGGTAACCAGTCCTTTTTTTCTTTTTTTATTTCCTGTGAGATCACCAACACACCCCACTTAGGACACTTGTGTATGGCAGAGGCCTGCAGATGTTTGCTTTCACGACCCACATGGAGGTGGAAGCAGCCAGACAGTGGTTAA

GTTGACAATTAATCATCGGCATAGTATATCGGCATAGTATAATACGACAAGGTGAGGAACTAAACC

ATGGCCAAGCCTTTGTCTCAAGAAGAATCCACCCTCATTGAAAGAGCAACGGCTACAATCAACAGCATCCCCATCTCTGAAGACTACAGCGTCGCCAGCGCAGCTCTCTCTAGCGACGGCCGCATCTTCACTGGTGTCAATGTATATCATTTTACTGGGGGACCTTGTGCAGAACTCGTGGTGCTGGGCACTGCTGCTGCTGCGGCAGCTGGCAACCTGCTTGTATCGTCGCGATCGGAAATGAGAACAGGGGCATCTTGAGCCCCTGCGGACGGTGTCGACAGGTGCTTCTCGATCTGCATCCTGGGATCAAAGCGATAGTGAAGGACAGTGATGGACAGCCGACGGCAGTTGGGATTCGTGAATTGCTGCCCTCTGGTTATGTGTGGGAGGGCTAA

CTGTGGATGTTCAAGCATCAGTTTGCCTGATCCTATAGGGCTGTCATGCTTTTACAATCT

GCCTGTTTCTAGAACCCACGCCTGCCCTCACCCACTGAGAGGTCTGGATCTCTGTTCCCA

CCTCCCCACTGAGTGGCTCAGCTCCCTGCCCACTTCTCTAATCACCTCCCCACTCTTGGC

CCGAGCCACCCACCTGCCCTGTTCCTTGCCTCCCTTCACTTTCTGAAGTATTTTCTGCTC

AGACTTCACAGG

*P2rx1* site A Gentamicin cassette:

CACCTGCTAGTTAGCATTGCAGCTCTGCCCTGCTGGGACAATCCTATACCCTGTTCTTGCTGGATCCTTCTTGTGCCTGCTTCTGTAGCTGCCTATGTGGGAATGGTAACCAGTCCTTTTTTTCTTTTTTTATTTCCTGTGAGATCACCAACACACCCCACTTAGGACACTTGTGTATGGCAGAGGCCTGCAGATGTTTGCTTTCACGACCCACATGGAGGTGGAAGCAGCCAGACAGTGGTTAA

GTTGACAATTAATCATCGGCATAGTATATCGGCATAGTATAATACGACAAGGTGAGGAACTAAACC

ATGTTACGCAGCAGCAACGATGTTACGCAGCAGGGCAGTCGCCCTAAAACAAAGTTAGGTGGCTCAAGTATGGGCATCATTCGCACATGTAGGCTCGGCCCTGACCAAGTCAAATCCATGCGGGCTGCTCTTGATCTTTTCGGTCGTGAGTTCGGAGACGTAGCCACCTACTCCCAACATCAGCCGGACTCCGATTACCTCGGGAACTTGCTCCGTAGTAAGACATTCATCGCGCTTGCTGCCTTCGACCAAGAAGCGGTTGTTGGCGCTCTCGCGGCTTACGTTCTGCCCAGGTTTGAGCAGCCGCGTAGTGAGATCTATATCTATGATCTCGCAGTCTCCGGCGAGCACCGGAGGCAGGGCATTGCCACCGCGCTCATCAATCTCCTCAAGCATGAGGCCAACGCGCTTGGTGCTTATGTGATCTACGTGCAAGCAGATTACGGTGACGATCCCGCAGTGGCTCTCTATACAAAGTTGGGCATACGGGAAGAAGTGATGCACTTTGATATCGACCCAAGTACCGCCACCTAA

CTGTGGATGTTCAAGCATCAGTTTGCCTGATCCTATAGGGCTGTCATGCTTTTACAATCT

GCCTGTTTCTAGAACCCACGCCTGCCCTCACCCACTGAGAGGTCTGGATCTCTGTTCCCA

CCTCCCCACTGAGTGGCTCAGCTCCCTGCCCACTTCTCTAATCACCTCCCCACTCTTGGC

CCGAGCCACCCACCTGCCCTGTTCCTTGCCTCCCTTCACTTTCTGAAGTATTTTCTGCTC

AGACTTCACAGG

*P2rx1* site A ends-in Blasticidin cassette:

TTAACCACTGTCTGGCTGCTTCCACCTCCATGTGGGTCGTGAAAGCAAACATCTGCAGGCCTCTGCCATACACAAGTGTCCTAAGTGGGGTGTGTTGGTGATCTCACAGGAAATAAAAAAAGAAAAAAAGGACTGGTTACCATTCCCACATAGGCAGCTACAGAAGCAGGCACAAG

GTTGACAATTAATCATCGGCATAGTATATCGGCATAGTATAATACGACAAGGTGAGGAACTAAACC

ATGGCCAAGCCTTTGTCTCAAGAAGAATCCACCCTCATTGAAAGAGCAACGGCTACAATCAACAGCATCCCCATCTCTGAAGACTACAGCGTCGCCAGCGCAGCTCTCTCTAGCGACGGCCGCATCTTCACTGGTGTCAATGTATATCATTTTACTGGGGGACCTTGTGCAGAACTCGTGGTGCTGGGCACTGCTGCTGCTGCGGCAGCTGGCAACCTGACTTGTATCGTCGCGATCGGAAATGAGAACAGGGGCATCTTGAGCCCCTGCGGACGGTGTCGACAGGTGCTTCTCGATCTGCATCCTGGGATCAAAGCGATAGTGAAGGACAGTGATGGACAGCCGACGGCAGTTGGGATTCGTGAATTGCTGCCCTCTGGTTATGTGTGGGAGGGCTAA

AAGAGTGGGGAGGTGATTAGAGAAGTGGGCAGGGAGCTGAGCCACTCAGTGGGGAGGTGGGAACAGAGATCCAGACCTCTCAGTGGGTGAGGGCAGGCGTGGGTTCTAGAAACAGGCAGATTGTAAAAGCATGACAGCCCTATAGGATCAGGCAAACTGATGCTTGAACATCCACAG

*P2rx1* site B Gentamicin cassette:

GATCTGCTTTTAATGACCATGGGTATAGGTGATGACCCTGGCCAGGCTTTGAGCCACTGCCATGGCTCTCCTACCATCTCCTTGGGTCGAGGGGAACAAGGTTAGAGTCAAAGCTCAGGTTTACTGACCTGGAGCTGGCTCTCAGGAGAGCCACAGCCCTGGATCACTGGTAGCAGGAATAGTGTGGAATTGTAGGAGGCAGGTTTGACTTTCC

GTTGACAATTAATCATCGGCATAGTATATCGGCATAGTATAATACGACAAGGTGAGGAACTAAACC

ATGTTACGCAGCAGCAACGATGTTACGCAGCAGGGCAGTCGCCCTAAAACAAAGTTAGGTGGCTCAAGTATGGGCATCATTCGCACATGTAGGCTCGGCCCTGACCAAGTCAAATCCATGCGGGCTGCTCTTGATCTTTTCGGTCGTGAGTTCGGAGACGTAGCCACCTACTCCCAACATCAGCCGGACTCCGATTACCTCGGGAACTTGCTCCGTAGTAAGACATTCATCGCGCTTGCTGCCTTCGACCAAGAAGCGGTTGTTGGCGCTCTCGCGGCTTACGTTCTGCCCAGGTTTGAGCAGCCGCGTAGTGAGATCTATATCTATGATCTCGCAGTCTCCGGCGAGCACCGGAGGCAGGGCATTGCCACCGCGCTCATCAATCTCCTCAAGCATGAGGCCAACGCGCTTGGTGCTTATGTGATCTACGTGCAAGCAGATTACGGTGACGATCCCGCAGTGGCTCTCTATACAAAGTTGGGCATACGGGAAGAAGTGATGCACTTTGATATCGACCCAAGTACCGCCACCTAA

CTAGCTGGGCCCCTTCTGTTGACCTGGCTTTCCCCCTGGGCCCTGCCCAACCAGGCTGGG

AAGTTTGACATCATCCCTACTATGACTACCATCGGCTCTGGGATTGGCATCTTTGGAGTG

GTAAGTACTGGGGATACATGTTTGCTGTCTGGAGTTTGGCTGCTACTCTTCTACACACAG

TAGCAGAACCCCAAAATGTGACCATAGCTACCTTTCATGAATGAAACAGC

*P2rx1* site B Zeocin cassette:

GATCTGCTTTTAATGACCATGGGTATAGGTGATGACCCTGGCCAGGCTTTGAGCCACTGCCATGGCTCTCCTACCATCTCCTTGGGTCGAGGGGAACAAGGTTAGAGTCAAAGCTCAGGTTTACTGACCTGGAGCTGGCTCTCAGGAGAGCCACAGCCCTGGATCACTGGTAGCAGGAATAGTGTGGAATTGTAGGAGGCAGGTTTGACTTTCC

GTTGACAATTAATCATCGGCATAGTATATCGGCATAGTATAATACGACAAGGTGAGGAACTAAACC

ATGGCCAAGTTGACCAGTGCCGTTCCGGTGCTCACCGCGCGCGACGTCGCCGGAGCGGTCGAGTTCTGGACCGACCGGCTCGGGTTCTCCCGGGACTTCGTGGAGGACGACTTCGCCGGTGTGGTCCGGGACGACGTGACCCTGTTCATCAGCGCGGTCCAGGACCAGGTGGTGCCGGACAACACCCTGGCCTGGGTGTGGGTGCGCGGCCTGGACGAGCTGTACGCCGAGTGGTCGGAGGTCGTGTCCACGAACTTCCGGGACGCCTCCGGGCCGGCCATGACCGAGATCGGCGAGCAGCCGTGGGGGCGGGAGTTCGCCCTGCGCGACCCGGCCGGCAACTGCGTGCACTTCGTGGCCGAGGAGCAGGACTGA

CTAGCTGGGCCCCTTCTGTTGACCTGGCTTTCCCCCTGGGCCCTGCCCAACCAGGCTGGG

AAGTTTGACATCATCCCTACTATGACTACCATCGGCTCTGGGATTGGCATCTTTGGAGTG

GTAAGTACTGGGGATACATGTTTGCTGTCTGGAGTTTGGCTGCTACTCTTCTACACACAG

TAGCAGAACCCCAAAATGTGACCATAGCTACCTTTCATGAATGAAACAGC

*P2rx1* site B Hygromycin cassette:

GATCTGCTTTTAATGACCATGGGTATAGGTGATGACCCTGGCCAGGCTTTGAGCCACTGCCATGGCTCTCCTACCATCTCCTTGGGTCGAGGGGAACAAGGTTAGAGTCAAAGCTCAGGTTTACTGACCTGGAGCTGGCTCTCAGGAGAGCCACAGCCCTGGATCACTGGTAGCAGGAATAGTGTGGAATTGTAGGAGGCAGGTTTGACTTTCC

GTTGACAATTAATCATCGGCATAGTATATCGGCATAGTATAATACGACAAGGTGAGGAACTAAACC

ATGGAAAAGCCTGAACTCACCGCGACGTCTGTCGAGAAGTTTCTGATCGAAAAGTTCGACAGCGTCTCCGACCTGATGCAGCTCTCGGAGGGCGAAGAATCTCGTGCTTTCAGCTTCGATGTAGGAGGGCGTGGATATGTCCTGCGGGTAAATAGCTGCGCCGATGGTTTCTACAAAGATCGTTATGTTTATCGGCACTTTGCATCGGCCGCGCTCCCGATTCCGGAAGTGCTTGACATTGGGGAGTTTAGCGAGAGCCTGACCTATTGCATCTCCCGCCGTTCACAGGGTGTCACGTTGCAAGACCTGCCTGAAACCGAACTGCCCGCTGTTCTACAACCGGTCGCGGAGGCTATGGATGCGATCGCTGCGGCCGATCTTAGCCAGACGAGCGGGTTCGGCCCATTCGGACCGCAAGGAATCGGTCAATACACTACATGGCGTGATTTCATATGCGCGATTGCTGATCCCCATGTGTATCACTGGCAAACTGTGATGGACGACACCGTCAGTGCGTCCGTCGCGCAGGCTCTCGATGAGCTGATGCTTTGGGCCGAGGACTGCCCCGAAGTCCGGCACCTCGTGCACGCGGATTTCGGCTCCAACAATGTCCTGACGGACAATGGCCGCATAACAGCGGTCATTGACTGGAGCGAGGCGATGTTCGGGGATTCCCAATACGAGGTCGCCAACATCTTCTTCTGGAGGCCGTGGTTGGCTTGTATGGAGCAGCAGACGCGCTACTTCGAGCGGAGGCATCCGGAGCTTGCAGGATCGCCCGCTCCGGGCGTATATGCTCCGCATTGGTCTTGACCAACTCTATCAGAGCTTGGTTGACGGCAATTTCGATGATGCAGCTTGGGCGCAGGGTCGATGCGACGCAATCGTCCGATCCGGAGCCGGGACTGTCGGGCGTACACAAATCGCCCGCAGAAGCGCGGCCGTCTGGACCGATGGCTGTGTAGAAGTACTCGCCGATAGTGGAAACCGACGCCCCAGCACTCGTCCGAGGGCAAAGGAATAG

CTAGCTGGGCCCCTTCTGTTGACCTGGCTTTCCCCCTGGGCCCTGCCCAACCAGGCTGGGAAGTTTGACATCATCCCTACTATGACTACCATCGGCTCTGGGATTGGCATCTTTGGAGTGGTAAGTACTGGGGATACATGTTTGCTGTCTGGAGTTTGGCTGCTACTCTTCTACACACAGTAGCAGAACCCCAAAATGTGACCATAGCTACCTTTCATGAATGAAACAGC

*P2rx1* site B ends-in Zeocin cassette:

GGAAAGTCAAACCTGCCTCCTACAATTCCACACTATTCCTGCTACCAGTGATCCAGGGCTGTGGCTCTCCTGAGAGCCAGCTCCAGGTCAGTAAACCTGAGCTTTGACTCTAACCTTGTTCCCCTCGACCCAAGGAGATGGTAGGAGAGCCATGGCAGTGGCTCAAAGCCTGGCCA

GTTGACAATTAATCATCGGCATAGTATATCGGCATAGTATAATACGACAAGGTGAGGAACTAAACC

ATGGCCAAGTTGACCAGTGCCGTTCCGGTGCTCACCGCGCGCGACGTCGCCGGAGCGGTCGAGTTCTGGACCGACCGGCTCGGGTTCTCCCGGGACTTCGTGGAGGACGACTTCGCCGGTGTGGTCCGGGACGACGTGACCCTGTTCATCAGCGCGGTCCAGGACCAGGTGGTGCCGGACAACACCCTGGCCTGGGTGTGGGTGCGCGGCCTGGACGAGCTGTACGCCGAGTGGTCGGAGGTCGTGTCCACGAACTTCCGGGACGCCTCCGGGCCGGCCATGACCGAGATCGGCGAGCAGCCGTGGGGGCGGGAGTTCGCCCTGCGCGACCCGGCCGGCAACTGCGTGCACTTCGTGGCCGAGGAGCAGGACTGA

TGTGTGTAGAAGAGTAGCAGCCAAACTCCAGACAGCAAACATGTATCCCCAGTACTTACCACTCCAAAGATGCCAATCCCAGAGCCGATGGTAGTCATAGTAGGGATGATGTCAAACTTCCCAGCCTGGTTGGGCAGGGCCCAGGGGGAAAGCCAGGTCAACAGAAGGGGCCCAGCTAG

*P2rx1* site C Neomycin cassette:

AAGAAGTTCAAGTATGCGGAGGACATGGGGCCTGGGGAGGTAGGTGAGCTTCATCTTCTTGAGCTCCCTTCAAAGACCTCAAGCTTGTCTGCCTGTTGGCAGGTGCTGTCCTGCAGGGCTTGGTGCTGCAGGGCAGTGCTTCTGGGGCCTGACTTGCGTTCCCACATTGCATACACAGGGTGAACGTGACCCCGCGGCCACCAGCTCCACTCTGGGCCTGCAGGAGAACATGAGGACCTCC

GTTGACAATTAATCATCGGCATAGTATATCGGCATAGTATAATACGACAAGGTGAGGAACTAAACC

ATGGGATCGGCCATTGAACAAGATGGATTGCACGCAGGTTCTCCGGCCGCTTGGGTGGAGAGGCTATTCGGCTATGACTGGGCACAACAGACAATCGGCTGCTCTGATGCCGCCGTGTTCCGGCTGTCAGCGCAGGGGCGCCCGGTTCTTTTTGTCAAGACCGACCTGTCCGGTGCCCTGAATGAACTGCAGGACGAGGCAGCGCGGCTATCGTGGCTGGCCACGACGGGCGTTCCTTGCGCAGCTGTGCTCGACGTTGTCACTGAAGCGGGAAGGGACTGGCTGCTATTGGGCGAAGTGCCGGGGCAGGATCTCCTGTCATCTCACCTTGCTCCTGCCGAGAAAGTATCCATCATGGCT

GATGCAATGCGGCGGCTGCATACGCTTGATCCGGCTACCTGCCCATTCGACCACCAAGCGAAACATCGCATCGAGCGAGCACGTACTCGGATGGAAGCCGGTCTTGTCGATCAGGATGATCTGGACGAAGAGCATCAGGGGCTCGCGCCAGCCGAACTGTTCGCCAGGCTCAAGGCGCGCATGCCCGACGGCGATGATCTCGTCGTGACCCATGGCGATGCCTGCTTGCCGAATATCATGGTGGAAAATGGCCGCTTTTCTGGATTCATCGACTGTGGCCGGCTGGGTGTGGCGGACCGCTATCAGGACATAGCGTTGGCTACCCGTGATATTGCTGAAGAGCTTGGCGGCGAATGGGCT

GACCGCTTCCTCGTGCTTTACGGTATCGCCGCTCCCGATTCGCAGCGCATCGCCTTCTATCGCCTTCTTGACGAGTTCTTCTGA

CCTTAATCTTGAGCTCCGGACTTGACTCAGTGTGTGGCTTCCAGCAAGGGCTGGTGGCTTTGAGCCAGGGCAGAGGTCATTGCCAGAGGCTTTCCTGCAAGGCAGATACCAGTTGCCCTCTGGTTCAGCCAAGACATTGGAATCCAGAGCTCTGGGTCCAGATCCACACTTCCCTTCCTGAGGGATGGCCCCTCAATTTTTCACTATTCTGGGTCCCTCTGGCTGGGTTCCTCACA

*P2rx1* site C Gentamicin cassette:

AAGAAGTTCAAGTATGCGGAGGACATGGGGCCTGGGGAGGTAGGTGAGCTTCATCTTCTTGAGCTCCCTTCAAAGACCTCAAGCTTGTCTGCCTGTTGGCAGGTGCTGTCCTGCAGGGCTTGGTGCTGCAGGGCAGTGCTTCTGGGGCCTGACTTGCGTTCCCACATTGCATACACAGGGTGAACGTGACCCCGCGGCCACCAGCTCCACTCTGGGCCTGCAGGAGAACATGAGGACCTCC

GTTGACAATTAATCATCGGCATAGTATATCGGCATAGTATAATACGACAAGGTGAGGAACTAAACC

ATGTTACGCAGCAGCAACGATGTTACGCAGCAGGGCAGTCGCCCTAAAACAAAGTTAGGTGGCTCAAGTATGGGCATCATTCGCACATGTAGGCTCGGCCCTGACCAAGTCAAATCCATGCGGGCTGCTCTTGATCTTTTCGGTCGTGAGTTCGGAGACGTAGCCACCTACTCCCAACATCAGCCGGACTCCGATTACCTCGGGAACTTGCTCCGTAGTAAGACATTCATCGCGCTTGCTGCCTTCGACCAAGAAGCGGTTGTTGGCGCTCTCGCGGCTTACGTTCTGCCCAGGTTTGAGCAGCCGCGTAGTGAGATCTATATCTATGATCTCGCAGTCTCCGGCGAGCACCGGAGGCAGGGCATTGCCACCGCGCTCATCAATCTCCTCAAGCATGAGGCCAACGCGCTTGGTGCTTATGTGATCTACGTGCAAGCAGATTACGGTGACGATCCCGCAGTGGCTCTCTATACAAAGTTGGGCATACGGGAAGAAGTGATGCACTTTGATATCGACCCAAGTACCGCCACCTAA

CCTTAATCTTGAGCTCCGGACTTGACTCAGTGTGTGGCTTCCAGCAAGGGCTGGTGGCTTTGAGCCAGGGCAGAGGTCATTGCCAGAGGCTTTCCTGCAAGGCAGATACCAGTTGCCCTCTGGTTCAGCCAAGACATTGGAATCCAGAGCTCTGGGTCCAGATCCACACTTCCCTTCCTGAGGGATGGCCCCTCAATTTTTCACTATTCTGGGTCCCTCTGGCTGGGTTCCTCACA

*P2rx1* site D Gentamicin cassette:

AAATGGCTGTTTTTTGCTTTCCTTTCAAGCCTGTCTGAAGTTCAGTGTGCTTGCCTGTGAGATGCACAGCTGCGCTGAACAGCTGAGATCCCTGTGAGAAGGCTGGTGAGGGGTGCCTGGGCCAAGATGAGCCAGTGAGCCTACAGCAGTCCCTGAGCTCTGTCTCTGCACAGGACAGGGCACCGGTGCTCTTCCCAATAGCAAGGAACAAGTTCTTCTGGC

GTTGACAATTAATCATCGGCATAGTATATCGGCATAGTATAATACGACAAGGTGAGGAACTAAACC

ATGTTACGCAGCAGCAACGATGTTACGCAGCAGGGCAGTCGCCCTAAAACAAAGTTAGGTGGCTCAAGTATGGGCATCATTCGCACATGTAGGCTCGGCCCTGACCAAGTCAAATCCATGCGGGCTGCTCTTGATCTTTTCGGTCGTGAGTTCGGAGACGTAGCCACCTACTCCCAACATCAGCCGGACTCCGATTACCTCGGGAACTTGCTCCGTAGTAAGACATTCATCGCGCTTGCTGCCTTCGACCAAGAAGCGGTTGTTGGCGCTCTCGCGGCTTACGTTCTGCCCAGGTTTGAGCAGCCGCGTAGTGAGATCTATATCTATGATCTCGCAGTCTCCGGCGAGCACCGGAGGCAGGGCATTGCCACCGCGCTCATCAATCTCCTCAAGCATGAGGCCAACGCGCTTGGTGCTTATGTGATCTACGTGCAAGCAGATTACGGTGACGATCCCGCAGTGGCTCTCTATACAAAGTTGGGCATACGGGAAGAAGTGATGCACTTTGATATCGACCCAAGTACCGCCACCTAA

TGTGGGGCAGTTGAGCACACCAGAAGCAGCCTTGAGGGAGCATGGTTGAGGGAGGGGCTTGTTGTCCCAGGGATCTGGGGACACTTCCACGGACATGAGGCTTGCAGCTGGAGAGTAGAGAAGGCCACATAGGCACACCCTCCAGCCTGGGCTCACCTCCAACCAGGGCTGCTAAGTACCTCCACCCCTGCTCTGTCTAGACCA

*P2rx1* Site D Zeocin cassette:

AAATGGCTGTTTTTTGCTTTCCTTTCAAGCCTGTCTGAAGTTCAGTGTGCTTGCCTGTGAGATGCACAGCTGCGCTGAACAGCTGAGATCCCTGTGAGAAGGCTGGTGAGGGGTGCCTGGGCCAAGATGAGCCAGTGAGCCTACAGCAGTCCCTGAGCTCTGTCTCTGCACAGGACAGGGCACCGGTGCTCTTCCCAATAGCAAGGAACAAGTTCTTCTGGC

GTTGACAATTAATCATCGGCATAGTATATCGGCATAGTATAATACGACAAGGTGAGGAACTAAACC

ATGGCCAAGTTGACCAGTGCCGTTCCGGTGCTCACCGCGCGCGACGTCGCCGGAGCGGTCGAGTTCTGGACCGACCGGCTCGGGTTCTCCCGGGACTTCGTGGAGGACGACTTCGCCGGTGTGGTCCGGGACGACGTGACCCTGTTCATCAGCGCGGTCCAGGACCAGGTGGTGCCGGACAACACCCTGGCCTGGGTGTGGGTGCGCGGCCTGGACGAGCTGTACGCCGAGTGGTCGGAGGTCGTGTCCACGAACTTCCGGGACGCCTCCGGGCCGGCCATGACCGAGATCGGCGAGCAGCCGTGGGGGCGGGAGTTCGCCCTGCGCGACCCGGCCGGCAACTGCGTGCACTTCGTGGCCGAGGAGCAGGACTGA

TGTGGGGCAGTTGAGCACACCAGAAGCAGCCTTGAGGGAGCATGGTTGAGGGAGGGGCTTGTTGTCCCAGGGATCTGGGGACACTTCCACGGACATGAGGCTTGCAGCTGGAGAGTAGAGAAGGCCACATAGGCACACCCTCCAGCCTGGGCTCACCTCCAACCAGGGCTGCTAAGTACCTCCACCCCTGCTCTGTCTAGACCA

*P2rx1* site D ends-in Gentamicin cassette:

GCCAGAAGAACTTGTTCCTTGCTATTGGGAAGAGCACCGGTGCCCTGTCCTGTGCAGAGACAGAGCTCAGGGACTGCTGTAGGCTCACTGGCTCATCTTGGCCCAGGCACCCCTCACCAGCCTTCTCACAGGGATCTCAGCTGTTCAGCGCAGCTGTGCATCTCACAGGCAAGCAC

GTTGACAATTAATCATCGGCATAGTATATCGGCATAGTATAATACGACAAGGTGAGGAACTAAACC

ATGTTACGCAGCAGCAACGATGTTACGCAGCAGGGCAGTCGCCCTAAAACAAAGTTAGGTGGCTCAAGTATGGGCATCATTCGCACATGTAGGCTCGGCCCTGACCAAGTCAAATCCATGCGGGCTGCTCTTGATCTTTTCGGTCGTGAGTTCGGAGACGTAGCCACCTACTCCCAACATCAGCCGGACTCCGATTACCTCGGGAACTTGCTCCGTAGTAAGACATTCATCGCGCTTGCTGCCTTCGACCAAGAAGCGGTTGTTGGCGCTCTCGCGGCTTACGTTCTGCCCAGGTTTGAGCAGCCGCGTAGTGAGATCTATATCTATGATCTCGCAGTCTCCGGCGAGCACCGGAGGCAGGGCATTGCCACCGCGCTCATCAATCTCCTCAAGCATGAGGCCAACGCGCTTGGTGCTTATGTGATCTACGTGCAAGCAGATTACGGTGACGATCCCGCAGTGGCTCTCTATACAAAGTTGGGCATACGGGAAGAAGTGATGCACTTTGATATCGACCCAAGTACCGCCACCTAA

CTTAGCAGCCCTGGTTGGAGGTGAGCCCAGGCTGGAGGGTGTGCCTATGTGGCCTTCTCTACTCTCCAGCTGCAAGCCTCATGTCCGTGGAAGTGTCCCCAGATCCCTGGGACAACAAGCCCCTCCCTCAACCATGCTCCCTCAAGGCTGCTTCTGGTGTGCTCAACTGCCCCACA

**Subcloning plasmids:**

Zeocin, Trimethoprim or Chloremphenicol resistance cassette

p15A, R6K or pBeloBAC11 vector sequence including replication origin

Homology regions

NotI site

p15A *zeo* *Chrm1*

TCAGTCCTGCTCCTCGGCCACGAAGTGCACGCAGTTGCCGGCCGGGTCGCGCAGGGCGAACTCCCGCCCCCACGGCTGCTCGCCGATCTCGGTCATGGCCGGCCCGGAGGCGTCCCGGAAGTTCGTGGACACGACCTCCGACCACTCGGCGTACAGCTCGTCCAGGCCGCGCACCCACACCCAGGCCAGGGTGTTGTCCGGCACCACCTGGTCCTGGACCGCGCTGATGAACAGGGTCACGTCGTCCCGGACCACACCGGCGAAGTCGTCCTCCACGAAGTCCCGGGAGAACCCGAGCCGGTCGGTCCAGAACTCGACCGCTCCGGCGACGTCGCGCGCGGTGAGCACCGGAACGGCACT

GGTCAACTTGGCCAT

GGTTTAGTTCCTCACCTTGTCGTATTATACTATGCCGATATACTATGCCGATGATTAATTGTCAAC

CGGTGACCCGGGTCTTAATTAATAAGATGATCTTCTTGAGATCGTTTTGGTCTGCGCGTAATCTCTTGCTCTGAAAACGAAAAAACCGCCTTGCAGGGCGGTTTTTCGAAGGTTCTCTGAGCTACCAACTCTTTGAACCGAGGTAACTGGCTTGGAGGAGCGCAGTCACCAAAACTTGTCCTTTCAGTTTAGCCTTAACCGGCGCATGACTTCAAGACTAACTCCTCTAAATCAATTACCAGTGGCTGCTGCCAGTGGTGCTTTTGCATGTCTTTCCGGGTTGGACTCAAGACGATAGTTACCGGATAAGGCGCAGCGGTCGGACTGAACGGGGGGTTCGTGCATACAGTCCAGCTTGGAGCGAACTGCCTACCCGGAACTGAGTGTCAGGCGTGGAATGAGACAAACGCGGCCATAACAGCGGAATGACACCGGTAAACCGAAAGGCAGGAACAGGAGAGCGCACGAGGGAGCCGCCAGGGGGAAACGCCTGGTATCTTTATAGTCCTGTCGGGTTTCGCCACCACTGATTTGAGCGTCAGATTTCGTGATGCTTGTCAGGGGGGCGGAGCCTATGGAAAAACGGCTTTGCCGCGGCCCTCTCACTTCCCTGTTAAGTATCTTCCTGGCATCTTCCAGGAAATCTCCGCCCCGTTCGTAAGCCATTTCCGCTCGCCGCAGTCGAACGACCGAGCGTAGCGAGTCAGTGAGCGAGGAAGCGGAATATATCCTGTATCACATATTCTGCTGACGCACCGGTGCAGCCTTTTTTCTCCTGCCACATGAAGCACTTCACTGACACCCTCATCAGTGCCAACATAGTAAGCCAGTATACACTCCGCTAGCGCTTAATTAACCTGCAGG

TCTCCAATCAGCCAGAGAGTCTACTTTAGTTCCTTTACAAGTCTTTGCTCAGCTCTGCTGTCCCAAAGCACATGGAGCTGACACAAAGGGGGCTGTACACAGTTTAAGAACAAGGAGACCTTAGTAATGCCTTCTCTCCGATGTTGCTGGGCCTCAGTTATTCCCTCTGGGCAATGGACATTAATCAACCCCAAATTCAG

GCGGCCGC

CTGGCCCTGGTATTTGGTATTTCTGCCTTCCTGGAACCACCATTCACACAGTTCCTCAAGCCAGAGAGCTATAAGTCAACCTAGACTCCTTCGCACTGCCTGTACCCAGTCTACCTCTAAACAATTCACGAACATATCCTGTTTTCTCCATTGCTCTTGCATACCGTGCTAAAACAATCCCTCAATTCAGACTTCAGCAT

p15A *zeo* *Dnttip1*

TCAGTCCTGCTCCTCGGCCACGAAGTGCACGCAGTTGCCGGCCGGGTCGCGCAGGGCGAACTCCCGCCCCCACGGCTGCTCGCCGATCTCGGTCATGGCCGGCCCGGAGGCGTCCCGGAAGTTCGTGGACACGACCTCCGACCACTCGGCGTACAGCTCGTCCAGGCCGCGCACCCACACCCAGGCCAGGGTGTTGTCCGGCACCACCTGGTCCTGGACCGCGCTGATGAACAGGGTCACGTCGTCCCGGACCACACCGGCGAAGTCGTCCTCCACGAAGTCCCGGGAGAACCCGAGCCGGTCGGTCCAGAACTCGACCGCTCCGGCGACGTCGCGCGCGGTGAGCACCGGAACGGCACT

GGTCAACTTGGCCAT

GGTTTAGTTCCTCACCTTGTCGTATTATACTATGCCGATATACTATGCCGATGATTAATTGTCAAC

CGGTGACCCGGGTCTTAATTAATAAGATGATCTTCTTGAGATCGTTTTGGTCTGCGCGTAATCTCTTGCTCTGAAAACGAAAAAACCGCCTTGCAGGGCGGTTTTTCGAAGGTTCTCTGAGCTACCAACTCTTTGAACCGAGGTAACTGGCTTGGAGGAGCGCAGTCACCAAAACTTGTCCTTTCAGTTTAGCCTTAACCGGCGCATGACTTCAAGACTAACTCCTCTAAATCAATTACCAGTGGCTGCTGCCAGTGGTGCTTTTGCATGTCTTTCCGGGTTGGACTCAAGACGATAGTTACCGGATAAGGCGCAGCGGTCGGACTGAACGGGGGGTTCGTGCATACAGTCCAGCTTGGAGCGAACTGCCTACCCGGAACTGAGTGTCAGGCGTGGAATGAGACAAACGCGGCCATAACAGCGGAATGACACCGGTAAACCGAAAGGCAGGAACAGGAGAGCGCACGAGGGAGCCGCCAGGGGGAAACGCCTGGTATCTTTATAGTCCTGTCGGGTTTCGCCACCACTGATTTGAGCGTCAGATTTCGTGATGCTTGTCAGGGGGGCGGAGCCTATGGAAAAACGGCTTTGCCGCGGCCCTCTCACTTCCCTGTTAAGTATCTTCCTGGCATCTTCCAGGAAATCTCCGCCCCGTTCGTAAGCCATTTCCGCTCGCCGCAGTCGAACGACCGAGCGTAGCGAGTCAGTGAGCGAGGAAGCGGAATATATCCTGTATCACATATTCTGCTGACGCACCGGTGCAGCCTTTTTTCTCCTGCCACATGAAGCACTTCACTGACACCCTCATCAGTGCCAACATAGTAAGCCAGTATACACTCCGCTAGCGCTTAATTAACCTGCAGG

CTGCTTATTTCTTACCTTAGCCATCTTTGCCTGTTGCCTTCCTTGACCTCTCTGCATCCAGTCAAGCCCTTACCCTGTGAATGTACTACAGCTCCTGTCCCCTCTTTCTCCTCACTGTTGCCACAGCCTAGGCCTGTCATTGCTTGACATTCTCATTTGTTATAGCAGCCTGTGAGCTACTCTCCTGTCTGCCATCCACA

GCGGCCGC

CACCACCACCCTCATTCACATAACATCCAAGGTTTCTCCATATGTGCACTGTAGCATGCATAAACACACACCACCACCATCATACACATAACATCCAAGGTTTCTACATATGTGCACTGTAGCATGCATAAACACACACCACCACCACCCTCATACACATAACATCCAAGGTTTCTCCATATGTGCACTGTAGCATGCAT

p15A *zeo or dhfrII P2rx1*

TCAGTCCTGCTCCTCGGCCACGAAGTGCACGCAGTTGCCGGCCGGGTCGCGCAGGGCGAACTCCCGCCCCCACGGCTGCTCGCCGATCTCGGTCATGGCCGGCCCGGAGGCGTCCCGGAAGTTCGTGGACACGACCTCCGACCACTCGGCGTACAGCTCGTCCAGGCCGCGCACCCACACCCAGGCCAGGGTGTTGTCCGGCACCACCTGGTCCTGGACCGCGCTGATGAACAGGGTCACGTCGTCCCGGACCACACCGGCGAAGTCGTCCTCCACGAAGTCCCGGGAGAACCCGAGCCGGTCGGTCCAGAACTCGACCGCTCCGGCGACGTCGCGCGCGGTGAGCACCGGAACGGCACT

GGTCAACTTGGCCAT

GGTTTAGTTCCTCACCTTGTCGTATTATACTATGCCGATATACTATGCCGATGATTAATTGTCAAC

Or

GGACTAGTGCAAAAAACCCCTCAAGACCCGTTTAGAGGCCCCAAGGGGTTATGCTAGTTTGAACTCAGTTGATGCGTTCAAGCGCCGCAACAGGATAAATCTGTACTGAGCCTGGGTGAGCCTCAGACTCGACGGCGTAGCCTTCGGGGGTCAAATTTGTGCAGTACCACCCGACAATCTGACCTTGCCAGGCGGCGCCGGATTTCTTGCGCACGCGATCTCCCATACCAAACGTGGCGTTCGATGGGAATACAAAATTGCCAGCAACTGGATTACTGACTTCATTGCTACTTCGTTCCATACTTTTCCTTTTTCAATATTATTGAAGCATTTATCAGGGTTATTGTCTCATCTGTCAAGATCTTC

CGGTGACCCGGGTCTTAATTAATAAGATGATCTTCTTGAGATCGTTTTGGTCTGCGCGTAATCTCTTGCTCTGAAAACGAAAAAACCGCCTTGCAGGGCGGTTTTTCGAAGGTTCTCTGAGCTACCAACTCTTTGAACCGAGGTAACTGGCTTGGAGGAGCGCAGTCACCAAAACTTGTCCTTTCAGTTTAGCCTTAACCGGCGCATGACTTCAAGACTAACTCCTCTAAATCAATTACCAGTGGCTGCTGCCAGTGGTGCTTTTGCATGTCTTTCCGGGTTGGACTCAAGACGATAGTTACCGGATAAGGCGCAGCGGTCGGACTGAACGGGGGGTTCGTGCATACAGTCCAGCTTGGAGCGAACTGCCTACCCGGAACTGAGTGTCAGGCGTGGAATGAGACAAACGCGGCCATAACAGCGGAATGACACCGGTAAACCGAAAGGCAGGAACAGGAGAGCGCACGAGGGAGCCGCCAGGGGGAAACGCCTGGTATCTTTATAGTCCTGTCGGGTTTCGCCACCACTGATTTGAGCGTCAGATTTCGTGATGCTTGTCAGGGGGGCGGAGCCTATGGAAAAACGGCTTTGCCGCGGCCCTCTCACTTCCCTGTTAAGTATCTTCCTGGCATCTTCCAGGAAATCTCCGCCCCGTTCGTAAGCCATTTCCGCTCGCCGCAGTCGAACGACCGAGCGTAGCGAGTCAGTGAGCGAGGAAGCGGAATATATCCTGTATCACATATTCTGCTGACGCACCGGTGCAGCCTTTTTTCTCCTGCCACATGAAGCACTTCACTGACACCCTCATCAGTGCCAACATAGTAAGCCAGTATACACTCCGCTAGCGCTTAATTAACCTGCAGG

AGGATACCAGACCTCAAGTGGCCTTATCAGCAGTGTGTCAGTGAAACTCAAGGGCTTGGCTGTGACCCAGCTCCAGGGCCTGGGACCCCAGGTCTGGGACGTGGCTGACTATGTCTTCCCAGCACATGTAAGCGGCACCATCCTTCCTCCCATAAGCCCTGGCTTAAGGCTCTTTGAGGGACCAGTCCAGCTTCCTGCTCTCTGGGCCTCAGTCTGCCTTACTGTGCGAC

GCGGCCGC

AGGACTGTGTCCCAGTGAGCTCCTCTCTTTGTCTTGGCCAGGTTCAGGGTGTGAGAGAAGCGCACAGCTGTGTCCTTGAAAATCCCAGGGAAAAGGAAGAGGCTGGGTCTTCTGCGCATCTCTACCTCTTCCATCCAACATACGCTGTGGGTCAGACTGTTCTAGACCCTACTACCTGCTCCTTGGTCCCCCACGCCCAGTCCTCAGACCAGGACTGGGGCTTATTTTAAACCTTATCCAAGTGAATTGC

pBeloBAC11 *zeo* or *chl P2rx1*

TCAGTCCTGCTCCTCGGCCACGAAGTGCACGCAGTTGCCGGCCGGGTCGCGCAGGGCGAACTCCCGCCCCCACGGCTGCTCGCCGATCTCGGTCATGGCCGGCCCGGAGGCGTCCCGGAAGTTCGTGGACACGACCTCCGACCACTCGGCGTACAGCTCGTCCAGGCCGCGCACCCACACCCAGGCCAGGGTGTTGTCCGGCACCACCTGGTCCTGGACCGCGCTGATGAACAGGGTCACGTCGTCCCGGACCACACCGGCGAAGTCGTCCTCCACGAAGTCCCGGGAGAACCCGAGCCGGTCGGTCCAGAACTCGACCGCTCCGGCGACGTCGCGCGCGGTGAGCACCGGAACGGCACT

GGTCAACTTGGCCAT

GGTTTAGTTCCTCACCTTGTCGTATTATACTATGCCGATATACTATGCCGATGATTAATTGTCAAC

or

TTACGCCCCGCCCTGCCACTCATCGCAGTACTGTTGTAATTCATTAAGCATTCTGCCGACATGGAAGCCATCACAAACGGCATGATGAACCTGAATCGCCAGCGGCATCAGCACCTTGTCGCCTTGCGTATAATATTTGCCCATGGTGAAAACGGGGGCGAAGAAGTTGTCCATATTGGCCACGTTTAAATCAAAACTGGTGAAACTCACCCAGGGATTGGCTGAGACGAAAAACATATTCTCAATAAACCCTTTAGGGAAATAGGCCAGGTTTTCACCGTAACACGCCACATCTTGCGAATATATGTGTAGAAACTGCCGGAAATCGTCGTGGTATTCACTCCAGAGCGATGAAAACGTTTCAGTTTGCTCATGGAAAACGGTGTAACAAGGGTGAACACTATCCCATATCACCAGCTCACCGTCTTTCATTGCCATACGGAATTCCGGATGAGCATTCATCAGGCGGGCAAGAATGTGAATAAAGGCCGGATAAAACTTGTGCTTATTTTTCTTTACGGTCTTTAAAAAGGCCGTAATATCCAGCTGAACGGTCTGGTTATAGGTACATTGAGCAACTGACTGAAATGCCTCAAAATGTTCTTTACGATGCCATTGGGATATATCAACGGTGGTATATCCAGTGATTTTTTTCTCCAT

TTTAGCTTCCTTAGCTCCTGAAAATCTCGATAACTCAAAAAATACGCCCGGTAGT

TTTAGCTTCCTTAGCTCCTGAAAATCTCGATAACTCAAAAAATACGCCCGGTAGTGATCTTATTTCATTATGGTGAAAGTTGGAACCTCTTACGTGCCGATCAACGTCTCATTTTCGCCAAAAGTTGGCCCAGGGCTTCCCGGTATCAACAGGGACACCAGGATTTATTTATTCTGCGAAGTGATCTTCCGTCACAGGTATTTATTCGCGATAAGCTCATGGAGCGGCGTAACCGTCGCACAGGAAGGACAGAGAAAGCGCGGATCTGGGAAGTGACGGACAGAACGGTCAGGACCTGGATTGGGGAGGCGGTTGCCGCCGCTGCTGCTGACGGTGTGACGTTCTCTGTTCCGGTCACACCACATACGTTCCGCCATTCCTATGCGATGCACATGCTGTATGCCGGTATACCGCTGAAAGTTCTGCAAAGCCTGATGGGACATAAGTCCATCAGTTCAACGGAAGTCTACACGAAGGTTTTTGCGCTGGATGTGGCTGCCCGGCACCGGGTGCAGTTTGCGATGCCGGAGTCTGATGCGGTTGCGATGCTGAAACAATTATCCTGAGAATAAATGCCTTGGCCTTTATATGGAAATGTGGAACTGAGTGGATATGCTGTTTTTGTCTGTTAAACAGAGAAGCTGGCTGTTATCCACTGAGAAGCGAACGAAACAGTCGGGAAAATCTCCCATTATCGTAGAGATCCGCATTATTAATCTCAGGAGCCTGTGTAGCGTTTATAGGAAGTAGTGTTCTGTCATGATGCCTGCAAGCGGTAACGAAAACGATTTGAATATGCCTTCAGGAACAATAGAAATCTTCGTGCGGTGTTACGTTGAAGTGGAGCGGATTATGTCAGCAATGGACAGAACAACCTAATGAACACAGAACCATGATGTGGTCTGTCCTTTTACAGCCAGTAGTGCTCGCCGCAGTCGAGCGACAGGGCGAAGCCCTCGAGTGAGCGAGGAAGCACCAGGGAACAGCACTTATATATTCTGCTTACACACGATGCCTGAAAAAACTTCCCTTGGGGTTATCCACTTATCCACGGGGATATTTTTATAATTATTTTTTTTATAGTTTTTAGATCTTCTTTTTTAGAGCGCCTTGTAGGCCTTTATCCATGCTGGTTCTAGAGAAGGTGTTGTGACAAATTGCCCTTTCAGTGTGACAAATCACCCTCAAATGACAGTCCTGTCTGTGACAAATTGCCCTTAACCCTGTGACAAATTGCCCTCAGAAGAAGCTGTTTTTTCACAAAGTTATCCCTGCTTATTGACTCTTTTTTATTTAGTGTGACAATCTAAAAACTTGTCA

CACTTCACATGGATCTGTCATGGCGGAAACAGCGGTTATCAATCACAAGAAACGTAAAAATAGCCCGCGAATCGTCCAGTCAAACGACCTCACTGAGGCGGCATATAGTCTCTCCCGGGATCAAAAACGTATGCTGTATCTGTTCGTTGACCAGATCAGAAAATCTGATGGCACCCTACAGGAACATGACGGTATCTGCGAGATCCATGTTGCTAAATATGCTGAAATATTCGGATTGACCTCTGCGGAAGCCAGTAAGGATATACGGCAGGCATTGAAGAGTTTCGCGGGGAAGGAAGTGGTTTTTTATCGCCCTGAAGAGGATGCCGGCGATGAAAAAGGCTATGAATCTTTTCCTTGGTTTATCAAACGTGCGCACAGTCCATCCAGAGGGCTTTACAGTGTACATATCAACCCATATCTCATTCCCTTCTTTATCGGGTTACAGAACCGGTTTACGCAGTTTCGGCTTAGTGAAACAAAAGAAATCACCAATCCGTATGCCATGCGTTTATACGAATCCCTGTGTCAGTATCGTAAGCCGGATGGCTCAGGCATCGTCTCTCTGAAAATCGACTGGATCATAGAGCGTTACCAGCTGCCTCAAAGTTACCAGCGTATGCCTGACTTCCGCCGCCGCTTCCTGCAGGTCTGTGTTAATGAGATCAACAGCAGAACTCCAATGCGCCTCTCATACATTGAGAAAAAGAAAGGCCGCCAGACGACTCATATCGTATTTTCCTTCCGCGATATCACTTCCATGACGACAGGATAGTCTGAGGGTTATCTGTCACAGATTTGAGGGTGGTTCGTCACATTTGTTCTGACCTACTGAGGGTAATTTGTCACAGTTTTGCTGTTTCCTTCAGCCTGCATGGATTTTCTCATACTTTTTGAACTGTAATTTTTAAGGAAGCCAAATTTGAGGGCAGTTTGTCACAGTTGATTTCCTTCTCTTTCCCTTCGTCATGTGACCTGATATCGGGGGTTAGTTCGTCATCATTGATGAGGGTTGATTATCACAGTTTATTACTCTGAATTGGCTATCCGCGTGTGTACCTCTACCTGGAGTTTTTCCCACGGTGGATATTTCTTCTTGCGCTGAGCGTAAGAGCTATCTGACAGAACAGTTCTTCTTTGCTTCCTCGCCAGTTCGCTCGCTATGCTCGGTTACACGGCTGCGGCGAGCGCTAGTGATAATAAGTGACTGAGGTATGTGCTCTTCTTATCTCCTTTTGTAGTGTTGCTCTTATTTTAAACAACTTTGCGGTTTTTTGATGACTTTGCGATTTTGTTGTTGCTTTGCAGTAAATTGCAAGATTTAATAAAAAAACGCAAAGCAATGATTAAAGGATGTTCAGAATGAAACTCATGGAAACACTTAACCAGTGCATAAACGCTGGTCATGAAATGACGAAGGCTATCGCCATTGCACAGTTTAATGATGACAGCCCGGAAGCGAGGAAAATAACCCGGCGCTGGAGAATAGGTGAAGCAGCGGATTTAGTTGGGGTTTCTTCTCAGGCTATCAGAGATGCCGAGAAAGCAGGGCGACTACCGCACCCGGATATGGAAATTCGAGGACGGGTTGAGCAACGTGTTGGTTATACAATTGAACAAATTAATCATATGCGTGATGTGTTTGGTACGCGATTGCGACGTGCTGAAGACGTATTTCCACCGGTGATCGGGGTTGCTGCCCATAAAGGTGGCGTTTACAAAACCTCAGTTTCTGTTCATCTTGCTCAGGATCTGGCTCTGAAGGGGCTACGTGTTTTGCTCGTGGAAGGTAACGACCCCCAGGGAACAGCCTCAATGTATCACGGATGGGTACCAGATCTTCATATTCATGCAGAAGACACTCTCCTGCCTTTCTATCTTGGGGAAAAGGACGATGTCACTTATGCAATAAAGCCCACTTGCTGGCCGGGGCTTGACATTATTCCTTCCTGTCTGGCTCTGCACCGTATTGAAACTGAGTTAATGGGCAAATTTGATGAAGGTAAACTGCCCACCGATCCACACCTGATGCTCCGACTGGCCATTGAAACTGTTGCTCATGACTATGATGTCATAGTTATTGACAGCGCGCCTAACCTGGGTATCGGCACGATTAATGTCGTATGTGCTGCTGATGTGCTGATTGTTCCCACGCCTGCTGAGTTGTTTGACTACACCTCCGCACTGCAGTTTTTCGATATGCTTCGTGATCTGCTCAAGAACGTTGATCTTAAAGGGTTCGAGCCTGATGTACGTATTTTGCTTACCAAATACAGCAATAGTAATGGCTCTCAGTCCCCGTGGATGGAGGAGCAAATTCGGGATGCCTGGGGAAGCATGGTTCTAAAAAATGTTGTACGTGAAACGGATGAAGTTGGTAAAGGTCAGATCCGGATGAGAACTGTTTTTGAACAGGCCATTGATCAACGCTCTTCAACTGGTGCCTGGAGAAATGCTCTTTCTATTTGGGAACCTGTCTGCAATGAAATTTTCGATCGTCTGATTAAACCACGCTGGGAGATTAGATAATGAAGCGTGCGCCTGTTATTCCAAAACATACGCTCAATACTCAACCGGTTGAAGATACTTCGTTATCGACACCAGCTGCCCCGATGGTGGATTCGTTAATTGCGCGCGTAGGAGTAATGGCTCGCGGTAATGCCATTACTTTGCCTGTATGTGGTCGGGATGTGAAGTTTACTCTTGAAGTGCTCCGGGGTGATAGTGTTGAGAAGACCTCTCGGGTATGGTCAGGTAATGAACGTGACCAGGAGCTGCTTACTGAGGACGCACTGGATGATCTCATCCCTTCTTTTCTACTGACTGGTCAACAGACACCGGCGTTCGGTCGAAGAGTATCTGGTGTCATAGAAATTGCCGATGGGAGTCGCCGTCGTAAAGCTGCTGCACTTACCGAAAGTGATTATCGTGTTCTGGTTGGCGAGCTGGATGATGAGCAGATGGCTGCATTATCCAGATTGGGTAACGATTATCGCCCAACAAGTGCTTATGAACGTGGTCAGCGTTATGCAAGCCGATTGCAGAATGAATTTGCTGGAAATATTTCTGCGCTGGCTGATGCGGAAAATATTTCACGTAAGATTATTACCCGCTGTATCAACACCGCCAAATTGCCTAAATCAGTTGTTGCTCTTTTTTCTCACCCCGGTGAACTATCTGCCCGGTCAGGTGATGCACTTCAAAAAGCCTTTACAGATAAAGAGGAATTACTTAAGCAGCAGGCATCTAACCTTCATGAGCAGAAAAAAGCTGGGGTGATATTTGAAGCTGAAGAAGTTA

TCACTCTTTTAACTTCTGTGCTTAAAACGTCATCTGCATCAAGAACTAGTTTAAGCTCACGACATCAGTTTGCTCCTGGAGCGACAGTATTGTATAAGGGCGATAAAATGGTGCTTAACCTGGACAGGTCTCGTGTTCCAACTGAGTGTATAGAGAAAATTGAGGCCATTCTTAAGGAACTTGAAAAGCCAGCACCCTGATGCGACCACGTTTTAGTCTACGTTTATCTGTCTTTACTTAATGTCCTTTGTTACAGGCCAGAAAGCATAACTGGCCTGAATATTCTCTCTGGGCCCACTGTTCCACTTGTATCGTCGGTCTGATAATCAGACTGGGACCACGGTCCCACTCGTATCGTCGGTCTGATTATTAGTC

AGGATACCAGACCTCAAGTGGCCTTATCAGCAGTGTGTCAGTGAAACTCAAGGGCTTGGCTGTGACCCAGCTCCAGGGCCTGGGACCCCAGGTCTGGGACGTGGCTGACTATGTCTTCCCAGCACATGTAAGCGGCACCATCCTTCCTCCCATAAGCCCTGGCTTAAGGCTCTTTGAGGGACCAGTCCAGCTTCCTGCTCTCTGGGCCTCAGTCTGCCTTACTGTGCGAC

GCGGCCGC

AGGACTGTGTCCCAGTGAGCTCCTCTCTTTGTCTTGGCCAGGTTCAGGGTGTGAGAGAAGCGCACAGCTGTGTCCTTGAAAATCCCAGGGAAAAGGAAGAGGCTGGGTCTTCTGCGCATCTCTACCTCTTCCATCCAACATACGCTGTGGGTCAGACTGTTCTAGACCCTACTACCTGCTCCTTGGTCCCCCACGCCCAGTCCTCAGACCAGGACTGGGGCTTATTTTAAACCTTATCCAAGTGAATTGC

p15A *zeo* *Hdac1*

TCAGTCCTGCTCCTCGGCCACGAAGTGCACGCAGTTGCCGGCCGGGTCGCGCAGGGCGAACTCCCGCCCCCACGGCTGCTCGCCGATCTCGGTCATGGCCGGCCCGGAGGCGTCCCGGAAGTTCGTGGACACGACCTCCGACCACTCGGCGTACAGCTCGTCCAGGCCGCGCACCCACACCCAGGCCAGGGTGTTGTCCGGCACCACCTGGTCCTGGACCGCGCTGATGAACAGGGTCACGTCGTCCCGGACCACACCGGCGAAGTCGTCCTCCACGAAGTCCCGGGAGAACCCGAGCCGGTCGGTCCAGAACTCGACCGCTCCGGCGACGTCGCGCGCGGTGAGCACCGGAACGGCACT

GGTCAACTTGGCCAT

GGTTTAGTTCCTCACCTTGTCGTATTATACTATGCCGATATACTATGCCGATGATTAATTGTCAAC

CGGTGACCCGGGTCTTAATTAATAAGATGATCTTCTTGAGATCGTTTTGGTCTGCGCGTAATCTCTTGCTCTGAAAACGAAAAAACCGCCTTGCAGGGCGGTTTTTCGAAGGTTCTCTGAGCTACCAACTCTTTGAACCGAGGTAACTGGCTTGGAGGAGCGCAGTCACCAAAACTTGTCCTTTCAGTTTAGCCTTAACCGGCGCATGACTTCAAGACTAACTCCTCTAAATCAATTACCAGTGGCTGCTGCCAGTGGTGCTTTTGCATGTCTTTCCGGGTTGGACTCAAGACGATAGTTACCGGATAAGGCGCAGCGGTCGGACTGAACGGGGGGTTCGTGCATACAGTCCAGCTTGGAGCGAACTGCCTACCCGGAACTGAGTGTCAGGCGTGGAATGAGACAAACGCGGCCATAACAGCGGAATGACACCGGTAAACCGAAAGGCAGGAACAGGAGAGCGCACGAGGGAGCCGCCAGGGGGAAACGCCTGGTATCTTTATAGTCCTGTCGGGTTTCGCCACCACTGATTTGAGCGTCAGATTTCGTGATGCTTGTCAGGGGGGCGGAGCCTATGGAAAAACGGCTTTGCCGCGGCCCTCTCACTTCCCTGTTAAGTATCTTCCTGGCATCTTCCAGGAAATCTCCGCCCCGTTCGTAAGCCATTTCCGCTCGCCGCAGTCGAACGACCGAGCGTAGCGAGTCAGTGAGCGAGGAAGCGGAATATATCCTGTATCACATATTCTGCTGACGCACCGGTGCAGCCTTTTTTCTCCTGCCACATGAAGCACTTCACTGACACCCTCATCAGTGCCAACATAGTAAGCCAGTATACACTCCGCTAGCGCTTAATTAACCTGCAGG

CAGCGCCTGGCAGCACTGTGGGTCTCTTATTGTGGCTGAGACAGAGAGCAGGTCAGAACAACCATCCCTTAGTGGTAACAGGTAGAGAGAGCTGCTGTTCTGTGAGTGACCTGCAGTGGGAACACTGTGACCTTCCCTGTCCCCAAATAATTCTCTTCTTGCTTCTCACCCTCATCTGACAGCCCCATTTCTCAATAGGA

GCGGCCGC

AGGAGGCTATACCATCTACCTTTGTCCAAACTGTATACGAACACATTTCTCAGAATGTGTCTCCAGCCTTAACCCATGAGTGGCTGCATTTGGTAGGATTTGGGGCCGTTCTAAGGTATCTCAGAGTTACAGTCAAATGACCTGGGAGGAATATTTATGACTTCAGAAGTTAATGACCTGACTCTTTCATAAGCGATCGC

p15A *zeo* neo*

TCAGTCCTGCTCCTCGGCCACGAAGTGCACGCAGTTGCCGGCCGGGTCGCGCAGGGCGAACTCCCGCCCCCACGGCTGCTCGCCGATCTCGGTCATGGCCGGCCCGGAGGCGTCCCGGAAGTTCGTGGACACGACCTCCGACCACTCGGCGTACAGCTCGTCCAGGCCGCGCACCCACACCCAGGCCAGGGTGTTGTCCGGCACCACCTGGTCCTGGACCGCGCTGATGAACAGGGTCACGTCGTCCCGGACCACACCGGCGAAGTCGTCCTCCACGAAGTCCCGGGAGAACCCGAGCCGGTCGGTCCAGAACTCGACCGCTCCGGCGACGTCGCGCGCGGTGAGCACCGGAACGGCACT

GGTCAACTTGGCCAT

GGTTTAGTTCCTCACCTTGTCGTATTATACTATGCCGATATACTATGCCGATGATTAATTGTCAAC

CGGTGACCCGGGTCTTAATTAATAAGATGATCTTCTTGAGATCGTTTTGGTCTGCGCGTAATCTCTTGCTCTGAAAACGAAAAAACCGCCTTGCAGGGCGGTTTTTCGAAGGTTCTCTGAGCTACCAACTCTTTGAACCGAGGTAACTGGCTTGGAGGAGCGCAGTCACCAAAACTTGTCCTTTCAGTTTAGCCTTAACCGGCGCATGACTTCAAGACTAACTCCTCTAAATCAATTACCAGTGGCTGCTGCCAGTGGTGCTTTTGCATGTCTTTCCGGGTTGGACTCAAGACGATAGTTACCGGATAAGGCGCAGCGGTCGGACTGAACGGGGGGTTCGTGCATACAGTCCAGCTTGGAGCGAACTGCCTACCCGGAACTGAGTGTCAGGCGTGGAATGAGACAAACGCGGCCATAACAGCGGAATGACACCGGTAAACCGAAAGGCAGGAACAGGAGAGCGCACGAGGGAGCCGCCAGGGGGAAACGCCTGGTATCTTTATAGTCCTGTCGGGTTTCGCCACCACTGATTTGAGCGTCAGATTTCGTGATGCTTGTCAGGGGGGCGGAGCCTATGGAAAAACGGCTTTGCCGCGGCCCTCTCACTTCCCTGTTAAGTATCTTCCTGGCATCTTCCAGGAAATCTCCGCCCCGTTCGTAAGCCATTTCCGCTCGCCGCAGTCGAACGACCGAGCGTAGCGAGTCAGTGAGCGAGGAAGCGGAATATATCCTGTATCACATATTCTGCTGACGCACCGGTGCAGCCTTTTTTCTCCTGCCACATGAAGCACTTCACTGACACCCTCATCAGTGCCAACATAGTAAGCCAGTATACACT CCGCTAGCGCTTAATTAACCTGCAGG

ATCAGCGTGAGACTACGATTCCATCAATGCCTGTCAAGGGCAAGTATTGACATGTCGTCGTAACCTGTAGAACGGAGTAACCTCGGTGTGCGGTTGTATGCCTGCTGTGGATTGCTGCTGGACAGCAAGCGAACCGGAATTGCCAGCTGGGGCGTCCTCTGGTAAGGTTGGGAAGCCCTGCAAAGTAAACTGGATGGCTT

GCGGCCGC

AGCGTCTCCGACCTGATGCAGCTCTCGGAGGGCGAAGAATCTCGTGCTTTCAGCTTCGATGTAGGAGGGCGTGGATATGTCCTGCGGGTAAATAGCTGCGCCGATGGTTTCTACAAAGATCGTTATGTTTATCGGCACTTTGCATCGGCCGCGCTCCCGATTCCGGAAGTGCTTGACATT

R6Kγ *zeo* *neo**

TCAGTCCTGCTCCTCGGCCACGAAGTGCACGCAGTTGCCGGCCGGGTCGCGCAGGGCGAACTCCCGCCCCCACGGCTGCTCGCCGATCTCGGTCATGGCCGGCCCGGAGGCGTCCCGGAAGTTCGTGGACACGACCTCCGACCACTCGGCGTACAGCTCGTCCAGGCCGCGCACCCACACCCAGGCCAGGGTGTTGTCCGGCACCACCTGGTCCTGGACCGCGCTGATGAACAGGGTCACGTCGTCCCGGACCACACCGGCGAAGTCGTCCTCCACGAAGTCCCGGGAGAACCCGAGCCGGTCGGTCCAGAACTCGACCGCTCCGGCGACGTCGCGCGCGGTGAGCACCGGAACGGCACT

GGTCAACTTGGCCAT

GGTTTAGTTCCTCACCTTGTCGTATTATACTATGCCGATATACTATGCCGATGATTAATTGTCAAC

TAATGACCCCGAAGCAGGGTTATGCAGCGGAAAACGGCCACGATGCGTCCGGCGTAGAGGATCTGAAGATCAGCAGTTCAACCTGTTGATAGTACGTACTAAGCTCTCATGTTTCACGTACTAAGCTCTCATGTTTAACGTACTAAGCTCTCATGTTTAACGAACTAAACCCTCATGGCTAACGTACTAAGCTCTCATGGCTAACGTACTAAGCTCTCATGTTTCACGTACTAAGCTCTCATGTTTGAACAATAAAATTAATATAAATCAGCAACTTAAATAGCCTCTAAGGTTTTAAGTTTTATAAGAAAAAAAAGAATATATAAGGCTTTTAAAGCTTTTAAGGTTTAACGGTTGTGGACAACAAGCCAGGGATGTAACGCACTGAGAAGCCCTTAGAGCCTCTCAAAGCAATTTTGAGTGACACAGGAACACTTAACGGCTGACATGGGAATTAGCTTCACGCTGCCGCAAGCACTCAGGGCGCAAGGGCTGCTAAAGGAAGCGGATCTCGAGCCATGGGC

ATCAGCGTGAGACTACGATTCCATCAATGCCTGTCAAGGGCAAGTATTGACATGTCGTCGTAACCTGTAGAACGGAGTAACCTCGGTGTGCGGTTGTATGCCTGCTGTGGATTGCTGCTGGACAGCAAGCGAACCGGAATTGCCAGCTGGGGCGTCCTCTGGTAAGGTTGGGAAGCCCTGCAAAGTAAACTGGATGGCTT

GCGGCCGC

AGCGTCTCCGACCTGATGCAGCTCTCGGAGGGCGAAGAATCTCGTGCTTTCAGCTTCGATGTAGGAGGGCGTGGATATGTCCTGCGGGTAAATAGCTGCGCCGATGGTTTCTACAAAGATCGTTATGTTTATCGGCACTTTGCATCGGCCGCGCTCCCGATTCCGGAAGTGCTTGACATT

Sequence of the Neo gap repair insert

ATCAGCGTGAGACTACGATTCCATCAATGCCTGTCAAGGGCAAGTATTGACATGTCGTCGTAACCTGTAGAACGGAGTAACCTCGGTGTGCGGTTGTATGCCTGCTGTGGATTGCTGCTGGACAGCAAGCGAACCGGAATTGCCAGCTGGGGCGTCCTCTGGTAAGGTTGGGAAGCCCTGCAAAGTAAACTGGATGGCTTTCTTGCCGCCAAGGATCTGATGGCGCAGGGGATCAAGATCTGATCAAGAGACAGGATGAGGATCGTTTCGCATGATTGAACAAGATGGATTGCACGCAGGTTCTCCGGCCGCTTGGGTGGAGAGGCTATTCGGCTATGACTGGGCACAACAGACAATCGG

CTGCTCTGATGCCGCCGTGTTCCGGCTGTCAGCGCAGGGGCGCCCGGTTCTTTTTGTCAAGACCGACCTGTCCGGTGCCCTGAATGAACTGCAGGACGAGGCAGCGCGGCTATCGTGGCTGGCCACGACGGGCGTTCCTTGCGCAGCTGTGCTCGACGTTGTCACTGAAGCGGGAAGGGACTGGCTGCTATTGGGCGAAGTGCCGGGGCAGGATCTCCTGTCATCTCACCTTGCTCCTGCCGAGAAAGTATCCATCATGGCTGATGCAATGCGGCGGCTGCATACGCTTGATCCGGCTACCTGCCCATTCGACCACCAAGCGAAACATCGCATCGAGCGAGCACGTACTCGGATGGAAGCCGGTCTTGTCGATCAGGATGATCTGGACGAAGGCATCAGGGGCTCGCGCCAGCCGAACTGTTCGCCAGGCTCAAGGCGCGCATGCCCGACGGCGAGGATCTCGTCGTGACCCATGGCGATGCCTGCTTGCCGAATATCATGGTGGAAAATGGCCGCTTTTCTGGATTCATCGACTGTGGCCGGCTGGGTGTGGCGGACCGCTATCAGGACATAGCGTTGGCTACCCGTGATATTGCTGAAGAGCTTGGCGGCGAATGGGCTGACCGCTTCCTCGTGCTTTACGGTATCGCCGCTCCCGATTCGCAGCGCATCGCCTTCTATCGCCTTCTTGACGAGTTCTTCTGAAGGAGGAAATCATCATGAAAAAGCCTGAACTCACCGCGTCGTCTGTCGAGAAGTTTCTGATCGAAAAGTTCGACAGCGTCTCCGACCTGATGCAGCTCTCGGAGGGCGAAGAATCTCGTGCTTTCAGCTTCGATGTAGGAGGGCGTGGATATGTCCTGCGGGTAAATAGCTGCGCCGATGGTTTCTACAAAGATCGTTATGTTTATCGGCACTTTGCATCGGCCGCGCTCCCGATTCCGGAAGTGCTTGACATT

| **Linear PCR cassettes** | **5' Primer** | | | **3' primer** |
| --- | --- | --- | --- | --- |
| **Insertion cassettes** |  | | |  |
| *P2rx1* site A *genta* or *bsd* | CACCTGCTAGTTAGCATTGC | | | CCTGTGAAGTCTGAGCAGAA |
| *P2rx1* site A ends-out *bsd* | CTTGTGCCTGCTTCTGTAGC | | | AAGAGTGGGGAGGTGATTAG |
| *P2rx1* site A ends-in *bsd* | TTAACCACTGTCTGGCTGCT | | | CTGTGGATGTTCAAGCATCA |
| *P2rx1* site B *genta* or *zeo* or *hyg* | GATCTGCTTTTAATGACCATGGGTA | | | GCTGTTTCATTCATGAAAGGTAGC |
| *P2rx1* site B ends-out *zeo* | TGGCCAGGCTTTGAGCCACT | | | TGTGTGTAGAAGAGTAGCAG |
| *P2rx1* site B ends-in *zeo* | GGAAAGTCAAACCTGCCTCC | | | CTAGCTGGGCCCCTTCTGTT |
| *P2rx1* site C *genta* or *neo* | AAGAAGTTCAAGTATGCGGAGGAC | | | TGTGAGGAACCCAGCCAGAG |
| *P2rx1* site D *genta* or *zeo* | AAATGGCTGTTTTTTGCTTTCC | | | TGGTCTAGACAGAGCAGGGGT |
| *P2rx1* site D ends-out *genta* | GTGCTTGCCTGTGAGATGCA | | | CTTAGCAGCCCTGGTTGGAG |
| *P2rx1* site D *genta* 50 | ACAGGGCACCGGTGCTCTTC | | | CTCAACCATGCTCCCTCAAGGC |
| *P2rx1* site D ends-in *genta* | GCCAGAAGAACTTGTTCCTT | | | TGTGGGGCAGTTGAGCACAC |
|  |  | | |  |
| **Subcloning plasmids** |  | | |  |
| p15A *Chrm1* | CTGGCCCTGGTATTTGGTAT | | | CTGAATTTGGGGTTGATTAA |
| p15A *Dnttip1* | CACCACCACCCTCATTCACA | | | TGTGGATGGCAGACAGGAGA |
| p15A, pBeloBAC11 *P2rx1* | AGGACTGTGTCCCAGTGAGC | | | GTCGCACAGTAAGGCAGACT |
| p15A *P2rx1* 50 | TCCTCAGACCAGGACTGGGG | | | TGAGTTTCACTGACACACTG |
| p15A *Hdac1* | AGGAGGCTATACCATCTAC | | | TCCTATTGAGAAATGGGGCT |
| p15A Neo* or R6K Neo* | AGCGTCTCCGACCTGATGCA | | | AAGCCATCCAGTTTACTTTGC |
|  |  | | |  |
| **PCR genotyping** |  | | |  |
|  |  | | |  |
| **Long range PCR oligos** |  | | | **Expected PCR fragment size (bp)** |
| Site A |  | | |  |
| *P2rx1* site A flank F | CCAATGTGGGAGCCATGAGGA | | | 1, 100, *bsd* cassette insertion;  1, 235, *genta* cassette insertion; |
| *P2rx1* site A flank R | GTCCAAGGGGACAGAACCACC | | |  |
| Site B |  | | |  |
| *P2rx1* site B flank F | AGGCACCTTCCTGCTTTGGA | | | 1, 176, *genta* cassette insertion;  1, 017, *zeo* cassette insertion;  1, 666, *hyg* cassette insertion |
| *P2rx1* site B flank R | TCCCCAGGTACACCGTTTCT | | |  |
|  |  |  |  |  |
| Site C |  | | |  |
| *P2rx1* site C flank F  *P2rx1* site C flank R | TGCCCCAGATTCCCATTCTCC | | | 1, 221, *genta* cassette insertion;  1, 491, *neo* cassette insertion |
|  | AGCCTGAGCCATAAGCACGA | | |  |
| Site D |  | | |  |
| *P2rx1* site D flank F | CCCTGATGAATCTTGGCTCCTTGCC | | | 1, 228, *genta* cassette insertion;  1, 069, *zeo* cassette insertion |
| *P2rx1* site D flank R | GGCCCTCACTGGCTACCCTT | | |  |
| **Insertion genotyping oligos** | | | |  |
|  | | |  |  |
| **Ends-out recombination** | | |  |  |
| Site A | | |  |  |
| *P2rx1* site A F | | | CCAATGTGGGAGCCATGAGGA | 415 |
| *P2rx1* *bsd* R | | | GGGATGCTGTTGATTGTAGCCGT |  |
| Site B | | |  |  |
| *P2rx1* site B F | | | GGCACCTTCCTGCTTTGGATGT | 399 |
| *P2rx1* *zeo* R | | | CCGGAACGGCACTGGTCAA |  |
| Site D | | |  |  |
| *P2rx1* site D F | | | TGAGGGCTGAGGAGGCAAGG | 581 |
| *P2rx1* *genta* R | | | GAAGGCAGCAAGCGCGATGA |  |
|  | | |  |  |
| **Ends-in recombination** | | |  |  |
| Site A | | |  |  |
| *P2rx1* site A *bsd* R | | | GGGATGCTGTTGATTGTAGCCGT | 738 |
| *P2rx1* site A flank R | | | GTCCAAGGGGACAGAACCACC |  |
| Site B | | |  |  |
| *P2rx1* site B *zeo* R | | | CCGGAACGGCACTGGTCAA | 577 |
| *P2rx1* site B flank R | | | TCCCCAGGTACACCGTTTCT |  |
| Site D | | |  |  |
| *P2rx1* site D *genta* R | | | GAAGGCAGCAAGCGCGATGA | 821 |
| *P2rx1* site D flank R | | | GGCCCTCACTGGCTACCCTT |  |
|  | | |  |  |
| **Site specific insertion and SPI test.**  The PCRs used the same common *genta* reverse primer (CTATGCCGATGATTAATTGTCAAC). | | | | |
|  | |  | |  |
| Site A | |  | |  |
| *P2rx1* site A F | | CCAATGTGGGAGCCATGAGGA | | 303 |
| Site B | |  | |  |
| *P2rx1* site B F | | GGCACCTTCCTGCTTTGGATGT | | 329 |
| Site C | |  | |  |
| *P2rx1* site C F | | TGCCCCAGATTCCCATTCTCC | | 372 |
| Site D | |  | |  |
| *P2rx1* site D F | | TGAGGGCTGAGGAGGCAAGG | | 293 |
|  | |  | |  |
| **Different loci gap repair test** | |  | |  |
|  | |  | |  |
| pBeloBAC11 *P2rx1* 5' F | | CCATCAATGCCTGTCAAGGGCAAGT | | 298 |
| pBeloBAC11 *P2rx1* 5' R | | ACACCCAGCAGACACCGCATC | |  |
|  | |  | |  |
| p15A *zeo P2rx1* 3' F | | TGTCAGGGAAGGGGTGTGTGT | | 353^a^ |
| p15A *zeo P2rx1* 3' R | | ACTTCGTGGCCGAGGAGCAG | |  |
|  | |  | |  |
| p15A *dhfrII P2rx1* 3' F | | TGTCAGGGAAGGGGTGTGTGT | | 373 |
| p15A *dhfrII P2rx1* 3' R | | CCTTGGGGCCTCTAAACGGG | |  |
|  | |  | |  |
| p15A *zeo* *Chrm1* 3' F | | CTCCTGGGGCCTTTGCTTTCA | | 456 |
| p15A *zeo* *Chrm1* 3' R | | GAGCTGTACGCCGAGTGGTC | |  |
|  | |  | |  |
| p15A *zeo Dnttip1* 3' F | | TGTGTCTGTCTTGGGAGGCTGT | | 628 |
| p15A *zeo Dnttip1* 3' R | | ACTTCGTGGCCGAGGAGCA | |  |
|  | |  | |  |
| p15A *zeo Hdac1* 3' F | | GGGCTGGAAACCAAACCCTTGA | | 480 |
| p15A *zeo Hdac1* 3' R | | ACTTCGTGGCCGAGGAGCA | |  |

^a^Aberrant recombinants contained a portion of the 3’end of the *P2rx1* subcloned region and produced two bands with this PCR assay (S9A Fig.). RE digests confirmed that these aberrant recombinants lacked the full length *P2rx1* subcloned region (S9B Fig.).
